# Comprehensive molecular characterization of mitochondrial genomes in human cancers

**DOI:** 10.1038/s41588-019-0557-x

**Published:** 2020-02-05

**Authors:** Yuan Yuan, Young Seok Ju, Youngwook Kim, Jun Li, Yumeng Wang, Christopher J. Yoon, Yang Yang, Inigo Martincorena, Chad J. Creighton, John N. Weinstein, Yanxun Xu, Leng Han, Hyung-Lae Kim, Hidewaki Nakagawa, Keunchil Park, Peter J. Campbell, Han Liang, PCAWG Consortium, Lauri A. Aaltonen, Federico Abascal, Adam Abeshouse, Hiroyuki Aburatani, David J. Adams, Nishant Agrawal, Keun Soo Ahn, Sung-Min Ahn, Hiroshi Aikata, Rehan Akbani, Kadir C. Akdemir, Hikmat Al-Ahmadie, Sultan T. Al-Sedairy, Fatima Al-Shahrour, Malik Alawi, Monique Albert, Kenneth Aldape, Ludmil B. Alexandrov, Adrian Ally, Kathryn Alsop, Eva G. Alvarez, Fernanda Amary, Samirkumar B. Amin, Brice Aminou, Ole Ammerpohl, Matthew J. Anderson, Yeng Ang, Davide Antonello, Pavana Anur, Samuel Aparicio, Elizabeth L. Appelbaum, Yasuhito Arai, Axel Aretz, Koji Arihiro, Shun-ichi Ariizumi, Joshua Armenia, Laurent Arnould, Sylvia Asa, Yassen Assenov, Gurnit Atwal, Sietse Aukema, J. Todd Auman, Miriam R. R. Aure, Philip Awadalla, Marta Aymerich, Gary D. Bader, Adrian Baez-Ortega, Matthew H. Bailey, Peter J. Bailey, Miruna Balasundaram, Saianand Balu, Pratiti Bandopadhayay, Rosamonde E. Banks, Stefano Barbi, Andrew P. Barbour, Jonathan Barenboim, Jill Barnholtz-Sloan, Hugh Barr, Elisabet Barrera, John Bartlett, Javier Bartolome, Claudio Bassi, Oliver F. Bathe, Daniel Baumhoer, Prashant Bavi, Stephen B. Baylin, Wojciech Bazant, Duncan Beardsmore, Timothy A. Beck, Sam Behjati, Andreas Behren, Beifang Niu, Cindy Bell, Sergi Beltran, Christopher Benz, Andrew Berchuck, Anke K. Bergmann, Erik N. Bergstrom, Benjamin P. Berman, Daniel M. Berney, Stephan H. Bernhart, Rameen Beroukhim, Mario Berrios, Samantha Bersani, Johanna Bertl, Miguel Betancourt, Vinayak Bhandari, Shriram G. Bhosle, Andrew V. Biankin, Matthias Bieg, Darell Bigner, Hans Binder, Ewan Birney, Michael Birrer, Nidhan K. Biswas, Bodil Bjerkehagen, Tom Bodenheimer, Lori Boice, Giada Bonizzato, Johann S. De Bono, Arnoud Boot, Moiz S. Bootwalla, Ake Borg, Arndt Borkhardt, Keith A. Boroevich, Ivan Borozan, Christoph Borst, Marcus Bosenberg, Mattia Bosio, Jacqueline Boultwood, Guillaume Bourque, Paul C. Boutros, G. Steven Bova, David T. Bowen, Reanne Bowlby, David D. L. Bowtell, Sandrine Boyault, Rich Boyce, Jeffrey Boyd, Alvis Brazma, Paul Brennan, Daniel S. Brewer, Arie B. Brinkman, Robert G. Bristow, Russell R. Broaddus, Jane E. Brock, Malcolm Brock, Annegien Broeks, Angela N. Brooks, Denise Brooks, Benedikt Brors, Søren Brunak, Timothy J. C. Bruxner, Alicia L. Bruzos, Alex Buchanan, Ivo Buchhalter, Christiane Buchholz, Susan Bullman, Hazel Burke, Birgit Burkhardt, Kathleen H. Burns, John Busanovich, Carlos D. Bustamante, Adam P. Butler, Atul J. Butte, Niall J. Byrne, Anne-Lise Børresen-Dale, Samantha J. Caesar-Johnson, Andy Cafferkey, Declan Cahill, Claudia Calabrese, Carlos Caldas, Fabien Calvo, Niedzica Camacho, Peter J. Campbell, Elias Campo, Cinzia Cantù, Shaolong Cao, Thomas E. Carey, Joana Carlevaro-Fita, Rebecca Carlsen, Ivana Cataldo, Mario Cazzola, Jonathan Cebon, Robert Cerfolio, Dianne E. Chadwick, Dimple Chakravarty, Don Chalmers, Calvin Wing Yiu Chan, Kin Chan, Michelle Chan-Seng-Yue, Vishal S. Chandan, David K. Chang, Stephen J. Chanock, Lorraine A. Chantrill, Aurélien Chateigner, Nilanjan Chatterjee, Kazuaki Chayama, Hsiao-Wei Chen, Jieming Chen, Ken Chen, Yiwen Chen, Zhaohong Chen, Andrew D. Cherniack, Jeremy Chien, Yoke-Eng Chiew, Suet-Feung Chin, Juok Cho, Sunghoon Cho, Jung Kyoon Choi, Wan Choi, Christine Chomienne, Zechen Chong, Su Pin Choo, Angela Chou, Angelika N. Christ, Elizabeth L. Christie, Eric Chuah, Carrie Cibulskis, Kristian Cibulskis, Sara Cingarlini, Peter Clapham, Alexander Claviez, Sean Cleary, Nicole Cloonan, Marek Cmero, Colin C. Collins, Ashton A. Connor, Susanna L. Cooke, Colin S. Cooper, Leslie Cope, Vincenzo Corbo, Matthew G. Cordes, Stephen M. Cordner, Isidro Cortés-Ciriano, Kyle Covington, Prue A. Cowin, Brian Craft, David Craft, Chad J. Creighton, Yupeng Cun, Erin Curley, Ioana Cutcutache, Karolina Czajka, Bogdan Czerniak, Rebecca A. Dagg, Ludmila Danilova, Maria Vittoria Davi, Natalie R. Davidson, Helen Davies, Ian J. Davis, Brandi N. Davis-Dusenbery, Kevin J. Dawson, Francisco M. De La Vega, Ricardo De Paoli-Iseppi, Timothy Defreitas, Angelo P. Dei Tos, Olivier Delaneau, John A. Demchok, Jonas Demeulemeester, German M. Demidov, Deniz Demircioğlu, Nening M. Dennis, Robert E. Denroche, Stefan C. Dentro, Nikita Desai, Vikram Deshpande, Amit G. Deshwar, Christine Desmedt, Jordi Deu-Pons, Noreen Dhalla, Neesha C. Dhani, Priyanka Dhingra, Rajiv Dhir, Anthony DiBiase, Klev Diamanti, Li Ding, Shuai Ding, Huy Q. Dinh, Luc Dirix, HarshaVardhan Doddapaneni, Nilgun Donmez, Michelle T. Dow, Ronny Drapkin, Oliver Drechsel, Ruben M. Drews, Serge Serge, Tim Dudderidge, Ana Dueso-Barroso, Andrew J. Dunford, Michael Dunn, Lewis Jonathan Dursi, Fraser R. Duthie, Ken Dutton-Regester, Jenna Eagles, Douglas F. Easton, Stuart Edmonds, Paul A. Edwards, Sandra E. Edwards, Rosalind A. Eeles, Anna Ehinger, Juergen Eils, Roland Eils, Adel El-Naggar, Matthew Eldridge, Kyle Ellrott, Serap Erkek, Georgia Escaramis, Shadrielle M. G. Espiritu, Xavier Estivill, Dariush Etemadmoghadam, Jorunn E. Eyfjord, Bishoy M. Faltas, Daiming Fan, Yu Fan, William C. Faquin, Claudiu Farcas, Matteo Fassan, Aquila Fatima, Francesco Favero, Nodirjon Fayzullaev, Ina Felau, Sian Fereday, Martin L. Ferguson, Vincent Ferretti, Lars Feuerbach, Matthew A. Field, J. Lynn Fink, Gaetano Finocchiaro, Cyril Fisher, Matthew W. Fittall, Anna Fitzgerald, Rebecca C. Fitzgerald, Adrienne M. Flanagan, Neil E. Fleshner, Paul Flicek, John A. Foekens, Kwun M. Fong, Nuno A. Fonseca, Christopher S. Foster, Natalie S. Fox, Michael Fraser, Scott Frazer, Milana Frenkel-Morgenstern, William Friedman, Joan Frigola, Catrina C. Fronick, Akihiro Fujimoto, Masashi Fujita, Masashi Fukayama, Lucinda A. Fulton, Robert S. Fulton, Mayuko Furuta, P. Andrew Futreal, Anja Füllgrabe, Stacey B. Gabriel, Steven Gallinger, Carlo Gambacorti-Passerini, Jianjiong Gao, Shengjie Gao, Levi Garraway, Øystein Garred, Erik Garrison, Dale W. Garsed, Nils Gehlenborg, Josep L. L. Gelpi, Joshy George, Daniela S. Gerhard, Clarissa Gerhauser, Jeffrey E. Gershenwald, Mark Gerstein, Moritz Gerstung, Gad Getz, Mohammed Ghori, Ronald Ghossein, Nasra H. Giama, Richard A. Gibbs, Bob Gibson, Anthony J. Gill, Pelvender Gill, Dilip D. Giri, Dominik Glodzik, Vincent J. Gnanapragasam, Maria Elisabeth Goebler, Mary J. Goldman, Carmen Gomez, Santiago Gonzalez, Abel Gonzalez-Perez, Dmitry A. Gordenin, James Gossage, Kunihito Gotoh, Ramaswamy Govindan, Dorthe Grabau, Janet S. Graham, Robert C. Grant, Anthony R. Green, Eric Green, Liliana Greger, Nicola Grehan, Sonia Grimaldi, Sean M. Grimmond, Robert L. Grossman, Adam Grundhoff, Gunes Gundem, Qianyun Guo, Manaswi Gupta, Shailja Gupta, Ivo G. Gut, Marta Gut, Jonathan Göke, Gavin Ha, Andrea Haake, David Haan, Siegfried Haas, Kerstin Haase, James E. Haber, Nina Habermann, Faraz Hach, Syed Haider, Natsuko Hama, Freddie C. Hamdy, Anne Hamilton, Mark P. Hamilton, Leng Han, George B. Hanna, Martin Hansmann, Nicholas J. Haradhvala, Olivier Harismendy, Ivon Harliwong, Arif O. Harmanci, Eoghan Harrington, Takanori Hasegawa, David Haussler, Steve Hawkins, Shinya Hayami, Shuto Hayashi, D. Neil Hayes, Stephen J. Hayes, Nicholas K. Hayward, Steven Hazell, Yao He, Allison P. Heath, Simon C. Heath, David Hedley, Apurva M. Hegde, David I. Heiman, Michael C. Heinold, Zachary Heins, Lawrence E. Heisler, Eva Hellstrom-Lindberg, Mohamed Helmy, Seong Gu Heo, Austin J. Hepperla, José María Heredia-Genestar, Carl Herrmann, Peter Hersey, Julian M. Hess, Holmfridur Hilmarsdottir, Jonathan Hinton, Satoshi Hirano, Nobuyoshi Hiraoka, Katherine A. Hoadley, Asger Hobolth, Ermin Hodzic, Jessica I. Hoell, Steve Hoffmann, Oliver Hofmann, Andrea Holbrook, Aliaksei Z. Holik, Michael A. Hollingsworth, Oliver Holmes, Robert A. Holt, Chen Hong, Eun Pyo Hong, Jongwhi H. Hong, Gerrit K. Hooijer, Henrik Hornshøj, Fumie Hosoda, Yong Hou, Volker Hovestadt, William Howat, Alan P. Hoyle, Ralph H. Hruban, Jianhong Hu, Taobo Hu, Xing Hua, Kuan-lin Huang, Mei Huang, Mi Ni Huang, Vincent Huang, Yi Huang, Wolfgang Huber, Thomas J. Hudson, Michael Hummel, Jillian A. Hung, David Huntsman, Ted R. Hupp, Jason Huse, Matthew R. Huska, Barbara Hutter, Carolyn M. Hutter, Daniel Hübschmann, Christine A. Iacobuzio-Donahue, Charles David Imbusch, Marcin Imielinski, Seiya Imoto, William B. Isaacs, Keren Isaev, Shumpei Ishikawa, Murat Iskar, S. M. Ashiqul Islam, Michael Ittmann, Sinisa Ivkovic, Jose M. G. Izarzugaza, Jocelyne Jacquemier, Valerie Jakrot, Nigel B. Jamieson, Gun Ho Jang, Se Jin Jang, Joy C. Jayaseelan, Reyka Jayasinghe, Stuart R. Jefferys, Karine Jegalian, Jennifer L. Jennings, Seung-Hyup Jeon, Lara Jerman, Yuan Ji, Wei Jiao, Peter A. Johansson, Amber L. Johns, Jeremy Johns, Rory Johnson, Todd A. Johnson, Clemency Jolly, Yann Joly, Jon G. Jonasson, Corbin D. Jones, David R. Jones, David T. W. Jones, Nic Jones, Steven J. M. Jones, Jos Jonkers, Young Seok Ju, Hartmut Juhl, Jongsun Jung, Malene Juul, Randi Istrup Juul, Sissel Juul, Natalie Jäger, Rolf Kabbe, Andre Kahles, Abdullah Kahraman, Vera B. Kaiser, Hojabr Kakavand, Sangeetha Kalimuthu, Christof von Kalle, Koo Jeong Kang, Katalin Karaszi, Beth Karlan, Rosa Karlić, Dennis Karsch, Katayoon Kasaian, Karin S. Kassahn, Hitoshi Katai, Mamoru Kato, Hiroto Katoh, Yoshiiku Kawakami, Jonathan D. Kay, Stephen H. Kazakoff, Marat D. Kazanov, Maria Keays, Electron Kebebew, Richard F. Kefford, Manolis Kellis, James G. Kench, Catherine J. Kennedy, Jules N. A. Kerssemakers, David Khoo, Vincent Khoo, Narong Khuntikeo, Ekta Khurana, Helena Kilpinen, Hark Kyun Kim, Hyung-Lae Kim, Hyung-Yong Kim, Hyunghwan Kim, Jaegil Kim, Jihoon Kim, Jong K. Kim, Youngwook Kim, Tari A. King, Wolfram Klapper, Kortine Kleinheinz, Leszek J. Klimczak, Stian Knappskog, Michael Kneba, Bartha M. Knoppers, Youngil Koh, Jan Komorowski, Daisuke Komura, Mitsuhiro Komura, Gu Kong, Marcel Kool, Jan O. Korbel, Viktoriya Korchina, Andrey Korshunov, Michael Koscher, Roelof Koster, Zsofia Kote-Jarai, Antonios Koures, Milena Kovacevic, Barbara Kremeyer, Helene Kretzmer, Markus Kreuz, Savitri Krishnamurthy, Dieter Kube, Kiran Kumar, Pardeep Kumar, Sushant Kumar, Yogesh Kumar, Ritika Kundra, Kirsten Kübler, Ralf Küppers, Jesper Lagergren, Phillip H. Lai, Peter W. Laird, Sunil R. Lakhani, Christopher M. Lalansingh, Emilie Lalonde, Fabien C. Lamaze, Adam Lambert, Eric Lander, Pablo Landgraf, Luca Landoni, Anita Langerød, Andrés Lanzós, Denis Larsimont, Erik Larsson, Mark Lathrop, Loretta M. S. Lau, Chris Lawerenz, Rita T. Lawlor, Michael S. Lawrence, Alexander J. Lazar, Ana Mijalkovic Lazic, Xuan Le, Darlene Lee, Donghoon Lee, Eunjung Alice Lee, Hee Jin Lee, Jake June-Koo Lee, Jeong-Yeon Lee, Juhee Lee, Ming Ta Michael Lee, Henry Lee-Six, Kjong-Van Lehmann, Hans Lehrach, Dido Lenze, Conrad R. Leonard, Daniel A. Leongamornlert, Ignaty Leshchiner, Louis Letourneau, Ivica Letunic, Douglas A. Levine, Lora Lewis, Tim Ley, Chang Li, Constance H. Li, Haiyan Irene Li, Jun Li, Lin Li, Shantao Li, Siliang Li, Xiaobo Li, Xiaotong Li, Xinyue Li, Yilong Li, Han Liang, Sheng-Ben Liang, Peter Lichter, Pei Lin, Ziao Lin, W. M. Linehan, Ole Christian Lingjærde, Dongbing Liu, Eric Minwei Liu, Fei-Fei Fei Liu, Fenglin Liu, Jia Liu, Xingmin Liu, Julie Livingstone, Dimitri Livitz, Naomi Livni, Lucas Lochovsky, Markus Loeffler, Georgina V. Long, Armando Lopez-Guillermo, Shaoke Lou, David N. Louis, Laurence B. Lovat, Yiling Lu, Yong-Jie Lu, Youyong Lu, Claudio Luchini, Ilinca Lungu, Xuemei Luo, Hayley J. Luxton, Andy G. Lynch, Lisa Lype, Cristina López, Carlos López-Otín, Eric Z. Ma, Yussanne Ma, Gaetan MacGrogan, Shona MacRae, Geoff Macintyre, Tobias Madsen, Kazuhiro Maejima, Andrea Mafficini, Dennis T. Maglinte, Arindam Maitra, Partha P. Majumder, Luca Malcovati, Salem Malikic, Giuseppe Malleo, Graham J. Mann, Luisa Mantovani-Löffler, Kathleen Marchal, Giovanni Marchegiani, Elaine R. Mardis, Adam A. Margolin, Maximillian G. Marin, Florian Markowetz, Julia Markowski, Jeffrey Marks, Tomas Marques-Bonet, Marco A. Marra, Luke Marsden, John W. M. Martens, Sancha Martin, Jose I. Martin-Subero, Iñigo Martincorena, Alexander Martinez-Fundichely, Yosef E. Maruvka, R. Jay Mashl, Charlie E. Massie, Thomas J. Matthew, Lucy Matthews, Erik Mayer, Simon Mayes, Michael Mayo, Faridah Mbabaali, Karen McCune, Ultan McDermott, Patrick D. McGillivray, Michael D. McLellan, John D. McPherson, John R. McPherson, Treasa A. McPherson, Samuel R. Meier, Alice Meng, Shaowu Meng, Andrew Menzies, Neil D. Merrett, Sue Merson, Matthew Meyerson, William Meyerson, Piotr A. Mieczkowski, George L. Mihaiescu, Sanja Mijalkovic, Tom Mikkelsen, Michele Milella, Linda Mileshkin, Christopher A. Miller, David K. Miller, Jessica K. Miller, Gordon B. Mills, Ana Milovanovic, Sarah Minner, Marco Miotto, Gisela Mir Arnau, Lisa Mirabello, Chris Mitchell, Thomas J. Mitchell, Satoru Miyano, Naoki Miyoshi, Shinichi Mizuno, Fruzsina Molnár-Gábor, Malcolm J. Moore, Richard A. Moore, Sandro Morganella, Quaid D. Morris, Carl Morrison, Lisle E. Mose, Catherine D. Moser, Ferran Muiños, Loris Mularoni, Andrew J. Mungall, Karen Mungall, Elizabeth A. Musgrove, Ville Mustonen, David Mutch, Francesc Muyas, Donna M. Muzny, Alfonso Muñoz, Jerome Myers, Ola Myklebost, Peter Möller, Genta Nagae, Adnan M. Nagrial, Hardeep K. Nahal-Bose, Hitoshi Nakagama, Hidewaki Nakagawa, Hiromi Nakamura, Toru Nakamura, Kaoru Nakano, Tannistha Nandi, Jyoti Nangalia, Mia Nastic, Arcadi Navarro, Fabio C. P. Navarro, David E. Neal, Gerd Nettekoven, Felicity Newell, Steven J. Newhouse, Yulia Newton, Alvin Wei Tian Ng, Anthony Ng, Jonathan Nicholson, David Nicol, Yongzhan Nie, G. Petur Nielsen, Morten Muhlig Nielsen, Serena Nik-Zainal, Michael S. Noble, Katia Nones, Paul A. Northcott, Faiyaz Notta, Brian D. O’Connor, Peter O’Donnell, Maria O’Donovan, Sarah O’Meara, Brian Patrick O’Neill, J. Robert O’Neill, David Ocana, Angelica Ochoa, Layla Oesper, Christopher Ogden, Hideki Ohdan, Kazuhiro Ohi, Lucila Ohno-Machado, Karin A. Oien, Akinyemi I. Ojesina, Hidenori Ojima, Takuji Okusaka, Larsson Omberg, Choon Kiat Ong, Stephan Ossowski, German Ott, B. F. Francis Ouellette, Christine P’ng, Marta Paczkowska, Salvatore Paiella, Chawalit Pairojkul, Marina Pajic, Qiang Pan-Hammarström, Elli Papaemmanuil, Irene Papatheodorou, Nagarajan Paramasivam, Ji Wan Park, Joong-Won Park, Keunchil Park, Kiejung Park, Peter J. Park, Joel S. Parker, Simon L. Parsons, Harvey Pass, Danielle Pasternack, Alessandro Pastore, Ann-Marie Patch, Iris Pauporté, Antonio Pea, John V. Pearson, Chandra Sekhar Pedamallu, Jakob Skou Pedersen, Paolo Pederzoli, Martin Peifer, Nathan A. Pennell, Charles M. Perou, Marc D. Perry, Gloria M. Petersen, Myron Peto, Nicholas Petrelli, Robert Petryszak, Stefan M. Pfister, Mark Phillips, Oriol Pich, Hilda A. Pickett, Todd D. Pihl, Nischalan Pillay, Sarah Pinder, Mark Pinese, Andreia V. Pinho, Esa Pitkänen, Xavier Pivot, Elena Piñeiro-Yáñez, Laura Planko, Christoph Plass, Paz Polak, Tirso Pons, Irinel Popescu, Olga Potapova, Aparna Prasad, Shaun R. Preston, Manuel Prinz, Antonia L. Pritchard, Stephenie D. Prokopec, Elena Provenzano, Xose S. Puente, Sonia Puig, Montserrat Puiggròs, Sergio Pulido-Tamayo, Gulietta M. Pupo, Colin A. Purdie, Michael C. Quinn, Raquel Rabionet, Janet S. Rader, Bernhard Radlwimmer, Petar Radovic, Benjamin Raeder, Keiran M. Raine, Manasa Ramakrishna, Kamna Ramakrishnan, Suresh Ramalingam, Benjamin J. Raphael, W. Kimryn Rathmell, Tobias Rausch, Guido Reifenberger, Jüri Reimand, Jorge Reis-Filho, Victor Reuter, Iker Reyes-Salazar, Matthew A. Reyna, Sheila M. Reynolds, Esther Rheinbay, Yasser Riazalhosseini, Andrea L. Richardson, Julia Richter, Matthew Ringel, Markus Ringnér, Yasushi Rino, Karsten Rippe, Jeffrey Roach, Lewis R. Roberts, Nicola D. Roberts, Steven A. Roberts, A. Gordon Robertson, Alan J. Robertson, Javier Bartolomé Rodriguez, Bernardo Rodriguez-Martin, F. Germán Rodríguez-González, Michael H. A. Roehrl, Marius Rohde, Hirofumi Rokutan, Gilles Romieu, Ilse Rooman, Tom Roques, Daniel Rosebrock, Mara Rosenberg, Philip C. Rosenstiel, Andreas Rosenwald, Edward W. Rowe, Romina Royo, Steven G. Rozen, Yulia Rubanova, Mark A. Rubin, Carlota Rubio-Perez, Vasilisa A. Rudneva, Borislav C. Rusev, Andrea Ruzzenente, Gunnar Rätsch, Radhakrishnan Sabarinathan, Veronica Y. Sabelnykova, Sara Sadeghi, S. Cenk Sahinalp, Natalie Saini, Mihoko Saito-Adachi, Gordon Saksena, Adriana Salcedo, Roberto Salgado, Leonidas Salichos, Richard Sallari, Charles Saller, Roberto Salvia, Michelle Sam, Jaswinder S. Samra, Francisco Sanchez-Vega, Chris Sander, Grant Sanders, Rajiv Sarin, Iman Sarrafi, Aya Sasaki-Oku, Torill Sauer, Guido Sauter, Robyn P. M. Saw, Maria Scardoni, Christopher J. Scarlett, Aldo Scarpa, Ghislaine Scelo, Dirk Schadendorf, Jacqueline E. Schein, Markus B. Schilhabel, Matthias Schlesner, Thorsten Schlomm, Heather K. Schmidt, Sarah-Jane Schramm, Stefan Schreiber, Nikolaus Schultz, Steven E. Schumacher, Roland F. Schwarz, Richard A. Scolyer, David Scott, Ralph Scully, Raja Seethala, Ayellet V. Segre, Iris Selander, Colin A. Semple, Yasin Senbabaoglu, Subhajit Sengupta, Elisabetta Sereni, Stefano Serra, Dennis C. Sgroi, Mark Shackleton, Nimish C. Shah, Sagedeh Shahabi, Catherine A. Shang, Ping Shang, Ofer Shapira, Troy Shelton, Ciyue Shen, Hui Shen, Rebecca Shepherd, Ruian Shi, Yan Shi, Yu-Jia Shiah, Tatsuhiro Shibata, Juliann Shih, Eigo Shimizu, Kiyo Shimizu, Seung Jun Shin, Yuichi Shiraishi, Tal Shmaya, Ilya Shmulevich, Solomon I. Shorser, Charles Short, Raunak Shrestha, Suyash S. Shringarpure, Craig Shriver, Shimin Shuai, Nikos Sidiropoulos, Reiner Siebert, Anieta M. Sieuwerts, Lina Sieverling, Sabina Signoretti, Katarzyna O. Sikora, Michele Simbolo, Ronald Simon, Janae V. Simons, Jared T. Simpson, Peter T. Simpson, Samuel Singer, Nasa Sinnott-Armstrong, Payal Sipahimalani, Tara J. Skelly, Marcel Smid, Jaclyn Smith, Karen Smith-McCune, Nicholas D. Socci, Heidi J. Sofia, Matthew G. Soloway, Lei Song, Anil K. Sood, Sharmila Sothi, Christos Sotiriou, Cameron M. Soulette, Paul N. Span, Paul T. Spellman, Nicola Sperandio, Andrew J. Spillane, Oliver Spiro, Jonathan Spring, Johan Staaf, Peter F. Stadler, Peter Staib, Stefan G. Stark, Lucy Stebbings, Ólafur Andri Stefánsson, Oliver Stegle, Lincoln D. Stein, Alasdair Stenhouse, Chip Stewart, Stephan Stilgenbauer, Miranda D. Stobbe, Michael R. Stratton, Jonathan R. Stretch, Adam J. Struck, Joshua M. Stuart, Henk G. Stunnenberg, Hong Su, Xiaoping Su, Ren X. Sun, Stephanie Sungalee, Hana Susak, Akihiro Suzuki, Fred Sweep, Monika Szczepanowski, Holger Sültmann, Takashi Yugawa, Angela Tam, David Tamborero, Benita Kiat Tee Tan, Donghui Tan, Patrick Tan, Hiroko Tanaka, Hirokazu Taniguchi, Tomas J. Tanskanen, Maxime Tarabichi, Roy Tarnuzzer, Patrick Tarpey, Morgan L. Taschuk, Kenji Tatsuno, Simon Tavaré, Darrin F. Taylor, Amaro Taylor-Weiner, Jon W. Teague, Bin Tean Teh, Varsha Tembe, Javier Temes, Kevin Thai, Sarah P. Thayer, Nina Thiessen, Gilles Thomas, Sarah Thomas, Alan Thompson, Alastair M. Thompson, John F. F. Thompson, R. Houston Thompson, Heather Thorne, Leigh B. Thorne, Adrian Thorogood, Grace Tiao, Nebojsa Tijanic, Lee E. Timms, Roberto Tirabosco, Marta Tojo, Stefania Tommasi, Christopher W. Toon, Umut H. Toprak, David Torrents, Giampaolo Tortora, Jörg Tost, Yasushi Totoki, David Townend, Nadia Traficante, Isabelle Treilleux, Jean-Rémi Trotta, Lorenz H. P. Trümper, Ming Tsao, Tatsuhiko Tsunoda, Jose M. C. Tubio, Olga Tucker, Richard Turkington, Daniel J. Turner, Andrew Tutt, Masaki Ueno, Naoto T. Ueno, Christopher Umbricht, Husen M. Umer, Timothy J. Underwood, Lara Urban, Tomoko Urushidate, Tetsuo Ushiku, Liis Uusküla-Reimand, Alfonso Valencia, David J. Van Den Berg, Steven Van Laere, Peter Van Loo, Erwin G. Van Meir, Gert G. Van den Eynden, Theodorus Van der Kwast, Naveen Vasudev, Miguel Vazquez, Ravikiran Vedururu, Umadevi Veluvolu, Shankar Vembu, Lieven P. C. Verbeke, Peter Vermeulen, Clare Verrill, Alain Viari, David Vicente, Caterina Vicentini, K. VijayRaghavan, Juris Viksna, Ricardo E. Vilain, Izar Villasante, Anne Vincent-Salomon, Tapio Visakorpi, Douglas Voet, Paresh Vyas, Ignacio Vázquez-García, Nick M. Waddell, Nicola Waddell, Claes Wadelius, Lina Wadi, Rabea Wagener, Jeremiah A. Wala, Jian Wang, Jiayin Wang, Linghua Wang, Qi Wang, Wenyi Wang, Yumeng Wang, Zhining Wang, Paul M. Waring, Hans-Jörg Warnatz, Jonathan Warrell, Anne Y. Warren, Sebastian M. Waszak, David C. Wedge, Dieter Weichenhan, Paul Weinberger, John N. Weinstein, Joachim Weischenfeldt, Daniel J. Weisenberger, Ian Welch, Michael C. Wendl, Johannes Werner, Justin P. Whalley, David A. Wheeler, Hayley C. Whitaker, Dennis Wigle, Matthew D. Wilkerson, Ashley Williams, James S. Wilmott, Gavin W. Wilson, Julie M. Wilson, Richard K. Wilson, Boris Winterhoff, Jeffrey A. Wintersinger, Maciej Wiznerowicz, Stephan Wolf, Bernice H. Wong, Tina Wong, Winghing Wong, Youngchoon Woo, Scott Wood, Bradly G. Wouters, Adam J. Wright, Derek W. Wright, Mark H. Wright, Chin-Lee Wu, Dai-Ying Wu, Guanming Wu, Jianmin Wu, Kui Wu, Yang Wu, Zhenggang Wu, Liu Xi, Tian Xia, Qian Xiang, Xiao Xiao, Rui Xing, Heng Xiong, Qinying Xu, Yanxun Xu, Hong Xue, Shinichi Yachida, Sergei Yakneen, Rui Yamaguchi, Takafumi N. Yamaguchi, Masakazu Yamamoto, Shogo Yamamoto, Hiroki Yamaue, Fan Yang, Huanming Yang, Jean Y. Yang, Liming Yang, Lixing Yang, Shanlin Yang, Tsun-Po Yang, Yang Yang, Xiaotong Yao, Marie-Laure Yaspo, Lucy Yates, Christina Yau, Chen Ye, Kai Ye, Venkata D. Yellapantula, Christopher J. Yoon, Sung-Soo Yoon, Fouad Yousif, Jun Yu, Kaixian Yu, Willie Yu, Yingyan Yu, Ke Yuan, Yuan Yuan, Denis Yuen, Christina K. Yung, Olga Zaikova, Jorge Zamora, Marc Zapatka, Jean C. Zenklusen, Thorsten Zenz, Nikolajs Zeps, Cheng-Zhong Zhang, Fan Zhang, Hailei Zhang, Hongwei Zhang, Hongxin Zhang, Jiashan Zhang, Jing Zhang, Junjun Zhang, Xiuqing Zhang, Xuanping Zhang, Yan Zhang, Zemin Zhang, Zhongming Zhao, Liangtao Zheng, Xiuqing Zheng, Wanding Zhou, Yong Zhou, Bin Zhu, Hongtu Zhu, Jingchun Zhu, Shida Zhu, Lihua Zou, Xueqing Zou, Anna deFazio, Nicholas van As, Carolien H. M. van Deurzen, Marc J. van de Vijver, L. van’t Veer, Christian von Mering

**Affiliations:** 1 Department of Bioinformatics and Computational Biology, The University of Texas MD Anderson Cancer Center, Houston, TX USA; 2 Cancer Genome Project, Wellcome Trust Sanger Institute, Hinxton, UK; 3 Graduate School of Medical Science and Engineering, Korea Advanced Institute of Science and Technology, Daejeon, Korea; 4 Department of Health Science and Technology, Samsung Advanced Institute for Health Science and Technology, Sungkyunkwan University School of Medicine, Seoul, Korea; 5 Samsung Genome Institute, Samsung Medical Center, Seoul, Korea; 6 Quantitative and Computational Biosciences Graduate Program, Baylor College of Medicine, Houston, TX USA; 7 Division of Biostatistics, The University of Texas Health Science Center at Houston School of Public Health, Houston, TX USA; 8 Department of Medicine and Dan L. Duncan Cancer Center Division of Biostatistics, Baylor College of Medicine, Houston, TX USA; 9 Department of Systems Biology, The University of Texas MD Anderson Cancer Center, Houston, TX USA; 10 Department of Applied Mathematics and Statistics, Johns Hopkins University, Baltimore, MD USA; 11 Department of Biochemistry and Molecular Biology, The University of Texas Health Science Center at Houston McGovern Medical School, Houston, TX USA; 12 Department of Biochemistry, Ewha Womans University School of Medicine, Seoul, Korea; 13 Laboratory for Cancer Genomics, RIKEN Center for Integrative Medical Sciences, Yokohama, Japan; 14 Division of Hematology/Oncology, Samsung Medical Center, Sungkyunkwan University School of Medicine, Seoul, Korea; 15 Samsung Advanced Institute for Health Sciences and Technology, Sungkyunkwan University School of Medicine, Seoul, South Korea; 16 Department of Haematology, University of Cambridge, Cambridge, UK; 200 Applied Tumor Genomics Research Program, Research Programs Unit, University of Helsinki, Helsinki, Finland; 201 Wellcome Sanger Institute, Wellcome Genome Campus, Hinxton, UK; 202 Memorial Sloan Kettering Cancer Center, New York, NY USA; 203 Genome Science Division, Research Center for Advanced Science and Technology, University of Tokyo, Tokyo, Japan; 204 Department of Surgery, University of Chicago, Chicago, IL USA; 205 Department of Surgery, Division of Hepatobiliary and Pancreatic Surgery, School of Medicine, Keimyung University Dongsan Medical Center, Daegu, South Korea; 206 Department of Oncology, Gil Medical Center, Gachon University, Incheon, South Korea; 207 Hiroshima University, Hiroshima, Japan; 208 Department of Bioinformatics and Computational Biology, The University of Texas MD Anderson Cancer Center, Houston, TX USA; 209 University of Texas MD Anderson Cancer Center, Houston, TX USA; 210 King Faisal Specialist Hospital and Research Centre, Al Maather, Riyadh, Saudi Arabia; 211 Bioinformatics Unit, Spanish National Cancer Research Centre (CNIO), Madrid, Spain; 212 Bioinformatics Core Facility, University Medical Center Hamburg, Hamburg, Germany; 213 Heinrich Pette Institute, Leibniz Institute for Experimental Virology, Hamburg, Germany; 214 Ontario Tumour Bank, Ontario Institute for Cancer Research, Toronto, ON Canada; 215 Department of Pathology, The University of Texas MD Anderson Cancer Center, Houston, TX USA; 216 Laboratory of Pathology, Center for Cancer Research, National Cancer Institute, Bethesda, MD USA; 217 Department of Cellular and Molecular Medicine and Department of Bioengineering, University of California San Diego, La Jolla, CA USA; 218 UC San Diego Moores Cancer Center, San Diego, CA USA; 219 Canada’s Michael Smith Genome Sciences Centre, BC Cancer, Vancouver, BC Canada; 220 Sir Peter MacCallum Department of Oncology, Peter MacCallum Cancer Centre, University of Melbourne, Melbourne, VIC Australia; 221 Centre for Research in Molecular Medicine and Chronic Diseases (CiMUS), Universidade de Santiago de Compostela, Santiago de Compostela, Spain; 222 Department of Zoology, Genetics and Physical Anthropology, (CiMUS), Universidade de Santiago de Compostela, Santiago de Compostela, Spain; 223 The Biomedical Research Centre (CINBIO), Universidade de Vigo, Vigo, Spain; 224 Royal National Orthopaedic Hospital - Bolsover, London, UK; 225 Department of Genomic Medicine, The University of Texas MD Anderson Cancer Center, Houston, TX USA; 226 Quantitative and Computational Biosciences Graduate Program, Baylor College of Medicine, Houston, TX USA; 227 The Jackson Laboratory for Genomic Medicine, Farmington, CT USA; 228 Genome Informatics Program, Ontario Institute for Cancer Research, Toronto, ON Canada; 229 Institute of Human Genetics, Christian-Albrechts-University, Kiel, Germany; 230 Institute of Human Genetics, Ulm University and Ulm University Medical Center, Ulm, Germany; 231 Queensland Centre for Medical Genomics, Institute for Molecular Bioscience, University of Queensland, St. Lucia, Brisbane, QLD Australia; 232 Salford Royal NHS Foundation Trust, Salford, UK; 233 Department of Surgery, Pancreas Institute, University and Hospital Trust of Verona, Verona, Italy; 234 Molecular and Medical Genetics, OHSU Knight Cancer Institute, Oregon Health and Science University, Portland, OR USA; 235 Department of Molecular Oncology, BC Cancer Research Centre, Vancouver, BC Canada; 236 The McDonnell Genome Institute at Washington University, St. Louis, MO USA; 237 University College London, London, UK; 238 Division of Cancer Genomics, National Cancer Center Research Institute, National Cancer Center, Tokyo, Japan; 239 DLR Project Management Agency, Bonn, Germany; 240 Tokyo Women’s Medical University, Tokyo, Japan; 241 Center for Molecular Oncology, Memorial Sloan Kettering Cancer Center, New York, NY USA; 242 Los Alamos National Laboratory, Los Alamos, NM USA; 243 Department of Pathology, University Health Network, Toronto General Hospital, Toronto, ON Canada; 244 Nottingham University Hospitals NHS Trust, Nottingham, UK; 245 Epigenomics and Cancer Risk Factors, German Cancer Research Center (DKFZ), Heidelberg, Germany; 246 Computational Biology Program, Ontario Institute for Cancer Research, Toronto, ON Canada; 247 Department of Molecular Genetics, University of Toronto, Toronto, ON Canada; 248 Vector Institute, Toronto, ON Canada; 249 Hematopathology Section, Institute of Pathology, Christian-Albrechts-University, Kiel, Germany; 250 Department of Pathology and Laboratory Medicine, School of Medicine, University of North Carolina at Chapel Hill, Chapel Hill, NC USA; 251 Department of Cancer Genetics, Institute for Cancer Research, Oslo University Hospital, The Norwegian Radium Hospital, Oslo, Norway; 252 Pathology, Hospital Clinic, Institut d’Investigacions Biomèdiques August Pi i Sunyer (IDIBAPS), University of Barcelona, Barcelona, Spain; 253 Department of Veterinary Medicine, Transmissible Cancer Group, University of Cambridge, Cambridge, UK; 254 Alvin J. Siteman Cancer Center, Washington University School of Medicine, St. Louis, MO USA; 255 Wolfson Wohl Cancer Research Centre, Institute of Cancer Sciences, University of Glasgow, Glasgow, UK; 256 Lineberger Comprehensive Cancer Center, University of North Carolina at Chapel Hill, Chapel Hill, NC USA; 257 Broad Institute of MIT and Harvard, Cambridge, MA USA; 258 Dana-Farber/Boston Children’s Cancer and Blood Disorders Center, Boston, MA USA; 259 Department of Pediatrics, Harvard Medical School, Boston, MA USA; 260 Leeds Institute of Medical Research @ St. James’s, University of Leeds, St. James’s University Hospital, Leeds, UK; 261 Department of Pathology and Diagnostics, University and Hospital Trust of Verona, Verona, Italy; 262 Department of Surgery, Princess Alexandra Hospital, Brisbane, QLD Australia; 263 Surgical Oncology Group, Diamantina Institute, University of Queensland, Brisbane, QLD Australia; 264 Department of Population and Quantitative Health Sciences, Case Western Reserve University School of Medicine, Cleveland, OH USA; 265 Research Health Analytics and Informatics, University Hospitals Cleveland Medical Center, Cleveland, OH USA; 266 Gloucester Royal Hospital, Gloucester, UK; 267 European Molecular Biology Laboratory, European Bioinformatics Institute (EMBL-EBI), Cambridge, UK; 268 Diagnostic Development, Ontario Institute for Cancer Research, Toronto, ON Canada; 269 Barcelona Supercomputing Center (BSC), Barcelona, Spain; 270 Arnie Charbonneau Cancer Institute, University of Calgary, Calgary, AB Canada; 271 Departments of Surgery and Oncology, University of Calgary, Calgary, AB Canada; 272 Department of Pathology, Oslo University Hospital, The Norwegian Radium Hospital, Oslo, Norway; 273 PanCuRx Translational Research Initiative, Ontario Institute for Cancer Research, Toronto, ON Canada; 274 Department of Oncology, Sidney Kimmel Comprehensive Cancer Center at Johns Hopkins University School of Medicine, Baltimore, MD USA; 275 University Hospital Southampton NHS Foundation Trust, Southampton, UK; 276 Royal Stoke University Hospital, Stoke-on-Trent, UK; 277 Genome Sequence Informatics, Ontario Institute for Cancer Research, Toronto, ON Canada; 278 Human Longevity Inc, San Diego, CA USA; 279 Olivia Newton-John Cancer Research Institute, La Trobe University, Heidelberg, VIC Australia; 280 Computer Network Information Center, Chinese Academy of Sciences, Beijing, China; 281 Genome Canada, Ottawa, ON Canada; 282 CNAG-CRG, Centre for Genomic Regulation (CRG), Barcelona Institute of Science and Technology (BIST), Barcelona, Spain; 283 Universitat Pompeu Fabra (UPF), Barcelona, Spain; 284 Buck Institute for Research on Aging, Novato, CA USA; 285 Duke University Medical Center, Durham, NC USA; 286 Department of Human Genetics, Hannover Medical School, Hannover, Germany; 287 Center for Bioinformatics and Functional Genomics, Cedars-Sinai Medical Center, Los Angeles, CA USA; 288 Department of Biomedical Sciences, Cedars-Sinai Medical Center, Los Angeles, CA USA; 289 The Hebrew University Faculty of Medicine, Jerusalem, Israel; 290 Barts Cancer Institute, Barts and the London School of Medicine and Dentistry, Queen Mary University of London, London, UK; 291 Department of Computer Science, Bioinformatics Group, University of Leipzig, Leipzig, Germany; 292 Interdisciplinary Center for Bioinformatics, University of Leipzig, Leipzig, Germany; 293 Transcriptome Bioinformatics, LIFE Research Center for Civilization Diseases, University of Leipzig, Leipzig, Germany; 294 Department of Medical Oncology, Dana-Farber Cancer Institute, Boston, MA USA; 295 Department of Cancer Biology, Dana-Farber Cancer Institute, Boston, MA USA; 296 Harvard Medical School, Boston, MA USA; 297 USC Norris Comprehensive Cancer Center, University of Southern California, Los Angeles, CA USA; 298 Department of Diagnostics and Public Health, University and Hospital Trust of Verona, Verona, Italy; 299 Department of Mathematics, Aarhus University, Aarhus, Denmark; 300 Department of Molecular Medicine (MOMA), Aarhus University Hospital, Aarhus N, Denmark; 301 Instituto Carlos Slim de la Salud, Mexico City, Mexico; 302 Department of Medical Biophysics, University of Toronto, Toronto, ON Canada; 303 Cancer Division, Garvan Institute of Medical Research, Kinghorn Cancer Centre, University of New South Wales (UNSW Sydney), Sydney, NSW Australia; 304 South Western Sydney Clinical School, Faculty of Medicine, University of New South Wales (UNSW Sydney), Liverpool, NSW Australia; 305 West of Scotland Pancreatic Unit, Glasgow Royal Infirmary, Glasgow, UK; 306 Center for Digital Health, Berlin Institute of Health and Charitè - Universitätsmedizin Berlin, Berlin, Germany; 307 Heidelberg Center for Personalized Oncology (DKFZ-HIPO), German Cancer Research Center (DKFZ), Heidelberg, Germany; 308 The Preston Robert Tisch Brain Tumor Center, Duke University Medical Center, Durham, NC USA; 309 Massachusetts General Hospital, Boston, MA USA; 310 National Institute of Biomedical Genomics, Kalyani, West Bengal India; 311 Institute of Clinical Medicine and Institute of Oral Biology, University of Oslo, Oslo, Norway; 312 University of North Carolina at Chapel Hill, Chapel Hill, NC USA; 313 ARC-Net Centre for Applied Research on Cancer, University and Hospital Trust of Verona, Verona, Italy; 314 The Institute of Cancer Research, London, UK; 315 Centre for Computational Biology, Duke-NUS Medical School, Singapore, Singapore; 316 Programme in Cancer and Stem Cell Biology, Duke-NUS Medical School, Singapore, Singapore; 317 Division of Oncology and Pathology, Department of Clinical Sciences Lund, Lund University, Lund, Sweden; 318 Department of Pediatric Oncology, Hematology and Clinical Immunology, Heinrich-Heine-University, Düsseldorf, Germany; 319 Laboratory for Medical Science Mathematics, RIKEN Center for Integrative Medical Sciences, Yokohama, Japan; 320 RIKEN Center for Integrative Medical Sciences, Yokohama, Japan; 321 Department of Internal Medicine/Hematology, Friedrich-Ebert-Hospital, Neumünster, Germany; 322 Departments of Dermatology and Pathology, Yale University, New Haven, CT USA; 323 Centre for Genomic Regulation (CRG), The Barcelona Institute of Science and Technology, Barcelona, Spain; 324 Radcliffe Department of Medicine, University of Oxford, Oxford, UK; 325 Canadian Center for Computational Genomics, McGill University, Montreal, QC Canada; 326 Department of Human Genetics, McGill University, Montreal, QC Canada; 327 Department of Human Genetics, University of California Los Angeles, Los Angeles, CA USA; 328 Department of Pharmacology, University of Toronto, Toronto, ON Canada; 329 Faculty of Medicine and Health Technology, Tampere University and Tays Cancer Center, Tampere University Hospital, Tampere, Finland; 330 Haematology, Leeds Teaching Hospitals NHS Trust, Leeds, UK; 331 Translational Research and Innovation, Centre Léon Bérard, Lyon, France; 332 Fox Chase Cancer Center, Philadelphia, PA USA; 333 International Agency for Research on Cancer, World Health Organization, Lyon, France; 334 Earlham Institute, Norwich, UK; 335 Norwich Medical School, University of East Anglia, Norwich, UK; 336 Department of Molecular Biology, Faculty of Science, Radboud Institute for Molecular Life Sciences, Radboud University, Nijmegen, HB The Netherlands; 337 CRUK Manchester Institute and Centre, Manchester, UK; 338 Department of Radiation Oncology, University of Toronto, Toronto, ON Canada; 339 Division of Cancer Sciences, Manchester Cancer Research Centre, University of Manchester, Manchester, UK; 340 Radiation Medicine Program, Princess Margaret Cancer Centre, Toronto, ON Canada; 341 Department of Pathology, Brigham and Women’s Hospital, Harvard Medical School, Boston, MA USA; 342 Department of Surgery, Division of Thoracic Surgery, The Johns Hopkins University School of Medicine, Baltimore, MD USA; 343 Division of Molecular Pathology, The Netherlands Cancer Institute, Oncode Institute, Amsterdam, CX The Netherlands; 344 Department of Biomolecular Engineering, University of California Santa Cruz, Santa Cruz, CA USA; 345 UC Santa Cruz Genomics Institute, University of California Santa Cruz, Santa Cruz, CA USA; 346 Division of Applied Bioinformatics, German Cancer Research Center (DKFZ), Heidelberg, Germany; 347 German Cancer Consortium (DKTK), German Cancer Research Center (DKFZ), Heidelberg, Germany; 348 National Center for Tumor Diseases (NCT) Heidelberg, Heidelberg, Germany; 349 Center for Biological Sequence Analysis, Department of Bio and Health Informatics, Technical University of Denmark, Lyngby, Denmark; 350 Novo Nordisk Foundation Center for Protein Research, University of Copenhagen, Copenhagen, Denmark; 351 Institute for Molecular Bioscience, University of Queensland, St. Lucia, Brisbane, QLD Australia; 352 Biomedical Engineering, Oregon Health and Science University, Portland, OR USA; 353 Division of Theoretical Bioinformatics, German Cancer Research Center (DKFZ), Heidelberg, Germany; 354 Institute of Pharmacy and Molecular Biotechnology and BioQuant, Heidelberg University, Heidelberg, Germany; 355 Federal Ministry of Education and Research, Berlin, Germany; 356 Melanoma Institute Australia, University of Sydney, Sydney, NSW Australia; 357 Pediatric Hematology and Oncology, University Hospital Muenster, Muenster, Germany; 358 Department of Pathology, Johns Hopkins University School of Medicine, Baltimore, MD USA; 359 McKusick-Nathans Institute of Genetic Medicine, Sidney Kimmel Comprehensive Cancer Center at Johns Hopkins University School of Medicine, Baltimore, MD USA; 360 Foundation Medicine, Inc, Cambridge, MA USA; 361 Department of Biomedical Data Science, Stanford University School of Medicine, Stanford, CA USA; 362 Department of Genetics, Stanford University School of Medicine, Stanford, CA USA; 363 Bakar Computational Health Sciences Institute and Department of Pediatrics, University of California, San Francisco, CA USA; 364 Institute of Clinical Medicine, Faculty of Medicine, University of Oslo, Oslo, Norway; 365 National Cancer Institute, National Institutes of Health, Bethesda, MD USA; 366 Royal Marsden NHS Foundation Trust, London and Sutton, UK; 367 Genome Biology Unit, European Molecular Biology Laboratory (EMBL), Heidelberg, Germany; 368 Department of Oncology, University of Cambridge, Cambridge, UK; 369 Li Ka Shing Centre, Cancer Research UK Cambridge Institute, University of Cambridge, Cambridge, UK; 370 Institut Gustave Roussy, Villejuif, France; 371 Cambridge University Hospitals NHS Foundation Trust, Cambridge, UK; 372 Department of Haematology, University of Cambridge, Cambridge, UK; 373 Anatomia Patológica, Hospital Clinic, Institut d’Investigacions Biomèdiques August Pi i Sunyer (IDIBAPS), University of Barcelona, Barcelona, Spain; 374 Spanish Ministry of Science and Innovation, Madrid, Spain; 375 University of Michigan Comprehensive Cancer Center, Ann Arbor, MI USA; 376 Department for BioMedical Research, University of Bern, Bern, Switzerland; 377 Department of Medical Oncology, Inselspital, University Hospital and University of Bern, Bern, Switzerland; 378 Graduate School for Cellular and Biomedical Sciences, University of Bern, Bern, Switzerland; 379 University of Pavia, Pavia, Italy; 380 University of Alabama at Birmingham, Birmingham, AL USA; 381 UHN Program in BioSpecimen Sciences, Toronto General Hospital, Toronto, ON Canada; 382 Department of Urology, Icahn School of Medicine at Mount Sinai, New York, NY USA; 383 Centre for Law and Genetics, University of Tasmania, Sandy Bay Campus, Hobart, TAS Australia; 384 Faculty of Biosciences, Heidelberg University, Heidelberg, Germany; 385 Department of Biochemistry, Microbiology and Immunology, Faculty of Medicine, University of Ottawa, Ottawa, ON Canada; 386 Division of Anatomic Pathology, Mayo Clinic, Rochester, MN USA; 387 Division of Cancer Epidemiology and Genetics, National Cancer Institute, National Institutes of Health, Bethesda, MD USA; 388 Illawarra Shoalhaven Local Health District L3 Illawarra Cancer Care Centre, Wollongong Hospital, Wollongong, NSW Australia; 389 BioForA, French National Institute for Agriculture, Food, and Environment (INRAE), ONF, Orléans, France; 390 Department of Biostatistics, Bloomberg School of Public Health, Johns Hopkins University, Baltimore, MD USA; 391 University of California San Diego, San Diego, CA USA; 392 Division of Experimental Pathology, Mayo Clinic, Rochester, MN USA; 393 Centre for Cancer Research, The Westmead Institute for Medical Research, University of Sydney, Sydney, NSW Australia; 394 Department of Gynaecological Oncology, Westmead Hospital, Sydney, NSW Australia; 395 PDXen Biosystems Inc, Seoul, South Korea; 396 Korea Advanced Institute of Science and Technology, Daejeon, South Korea; 397 Electronics and Telecommunications Research Institute, Daejeon, South Korea; 398 Institut National du Cancer (INCA), Boulogne-Billancourt, France; 399 Department of Genetics, Informatics Institute, University of Alabama at Birmingham, Birmingham, AL USA; 400 Division of Medical Oncology, National Cancer Centre, Singapore, Singapore; 401 Medical Oncology, University and Hospital Trust of Verona, Verona, Italy; 402 Department of Pediatrics, University Hospital Schleswig-Holstein, Kiel, Germany; 403 Hepatobiliary/Pancreatic Surgical Oncology Program, University Health Network, Toronto, ON Canada; 404 School of Biological Sciences, University of Auckland, Auckland, New Zealand; 405 Department of Surgery, University of Melbourne, Parkville, VIC Australia; 406 The Murdoch Children’s Research Institute, Royal Children’s Hospital, Parkville, VIC Australia; 407 Walter and Eliza Hall Institute, Parkville, VIC Australia; 408 Vancouver Prostate Centre, Vancouver, Canada; 409 Lunenfeld-Tanenbaum Research Institute, Mount Sinai Hospital, Toronto, ON Canada; 410 University of East Anglia, Norwich, UK; 411 Norfolk and Norwich University Hospital NHS Trust, Norwich, UK; 412 Victorian Institute of Forensic Medicine, Southbank, VIC Australia; 413 Department of Biomedical Informatics, Harvard Medical School, Boston, MA USA; 414 Department of Chemistry, Centre for Molecular Science Informatics, University of Cambridge, Cambridge, UK; 415 Ludwig Center at Harvard Medical School, Boston, MA USA; 416 Human Genome Sequencing Center, Baylor College of Medicine, Houston, TX USA; 417 Peter MacCallum Cancer Centre, University of Melbourne, Melbourne, VIC Australia; 418 Physics Division, Optimization and Systems Biology Lab, Massachusetts General Hospital, Boston, MA USA; 419 Department of Medicine, Baylor College of Medicine, Houston, TX USA; 420 University of Cologne, Cologne, Germany; 421 International Genomics Consortium, Phoenix, AZ USA; 422 Genomics Research Program, Ontario Institute for Cancer Research, Toronto, ON Canada; 423 Barking Havering and Redbridge University Hospitals NHS Trust, Romford, UK; 424 Children’s Hospital at Westmead, University of Sydney, Sydney, NSW Australia; 425 Department of Medicine, Section of Endocrinology, University and Hospital Trust of Verona, Verona, Italy; 426 Computational Biology Center, Memorial Sloan Kettering Cancer Center, New York, NY USA; 427 Department of Biology, ETH Zurich, Zürich, Switzerland; 428 Department of Computer Science, ETH Zurich, Zurich, Switzerland; 429 SIB Swiss Institute of Bioinformatics, Lausanne, Switzerland; 430 Weill Cornell Medical College, New York, NY USA; 431 Academic Department of Medical Genetics, University of Cambridge, Addenbrooke’s Hospital, Cambridge, UK; 432 MRC Cancer Unit, University of Cambridge, Cambridge, UK; 433 Departments of Pediatrics and Genetics, University of North Carolina at Chapel Hill, Chapel Hill, NC USA; 434 Seven Bridges Genomics, Charlestown, MA USA; 435 Annai Systems, Inc, Carlsbad, CA USA; 436 Department of Pathology, General Hospital of Treviso, Department of Medicine, University of Padua, Treviso, Italy; 437 Department of Computational Biology, University of Lausanne, Lausanne, Switzerland; 438 Department of Genetic Medicine and Development, University of Geneva Medical School, Geneva, CH Switzerland; 439 Swiss Institute of Bioinformatics, University of Geneva, Geneva, CH Switzerland; 440 The Francis Crick Institute, London, UK; 441 University of Leuven, Leuven, Belgium; 442 Institute of Medical Genetics and Applied Genomics, University of Tübingen, Tübingen, Germany; 443 Computational and Systems Biology, Genome Institute of Singapore, Singapore, Singapore; 444 School of Computing, National University of Singapore, Singapore, Singapore; 445 Big Data Institute, Li Ka Shing Centre, University of Oxford, Oxford, UK; 446 Biomedical Data Science Laboratory, Francis Crick Institute, London, UK; 447 Bioinformatics Group, Department of Computer Science, University College London, London, UK; 448 The Edward S. Rogers Sr. Department of Electrical and Computer Engineering, University of Toronto, Toronto, ON Canada; 449 Breast Cancer Translational Research Laboratory JC Heuson, Institut Jules Bordet, Brussels, Belgium; 450 Department of Oncology, Laboratory for Translational Breast Cancer Research, KU Leuven, Leuven, Belgium; 451 Institute for Research in Biomedicine (IRB Barcelona), The Barcelona Institute of Science and Technology, Barcelona, Spain; 452 Research Program on Biomedical Informatics, Universitat Pompeu Fabra, Barcelona, Spain; 453 Division of Medical Oncology, Princess Margaret Cancer Centre, Toronto, ON Canada; 454 Department of Physiology and Biophysics, Weill Cornell Medicine, New York, NY USA; 455 Institute for Computational Biomedicine, Weill Cornell Medicine, New York, NY USA; 456 Department of Pathology, UPMC Shadyside, Pittsburgh, PA USA; 457 Independent Consultant, Wellesley, USA; 458 Department of Cell and Molecular Biology, Science for Life Laboratory, Uppsala University, Uppsala, Sweden; 459 Department of Medicine and Department of Genetics, Washington University School of Medicine, St. Louis, St. Louis, MO USA; 460 Hefei University of Technology, Anhui, China; 461 Translational Cancer Research Unit, GZA Hospitals St.-Augustinus, Center for Oncological Research, Faculty of Medicine and Health Sciences, University of Antwerp, Antwerp, Belgium; 462 Simon Fraser University, Burnaby, BC Canada; 463 University of Pennsylvania, Philadelphia, PA USA; 464 Faculty of Science and Technology, University of Vic—Central University of Catalonia (UVic-UCC), Vic, Spain; 465 The Wellcome Trust, London, UK; 466 The Hospital for Sick Children, Toronto, ON Canada; 467 Department of Pathology, Queen Elizabeth University Hospital, Glasgow, UK; 468 Department of Genetics and Computational Biology, QIMR Berghofer Medical Research Institute, Brisbane, QLD Australia; 469 Department of Oncology, Centre for Cancer Genetic Epidemiology, University of Cambridge, Cambridge, UK; 470 Department of Public Health and Primary Care, Centre for Cancer Genetic Epidemiology, University of Cambridge, Cambridge, UK; 471 Prostate Cancer Canada, Toronto, ON Canada; 472 University of Cambridge, Cambridge, UK; 473 Department of Laboratory Medicine, Translational Cancer Research, Lund University Cancer Center at Medicon Village, Lund University, Lund, Sweden; 474 Heidelberg University, Heidelberg, Germany; 475 New BIH Digital Health Center, Berlin Institute of Health (BIH) and Charité - Universitätsmedizin Berlin, Berlin, Germany; 476 CIBER Epidemiología y Salud Pública (CIBERESP), Madrid, Spain; 477 Research Group on Statistics, Econometrics and Health (GRECS), UdG, Barcelona, Spain; 478 Quantitative Genomics Laboratories (qGenomics), Barcelona, Spain; 479 Icelandic Cancer Registry, Icelandic Cancer Society, Reykjavik, Iceland; 480 State Key Laboratory of Cancer Biology, and Xijing Hospital of Digestive Diseases, Fourth Military Medical University, Shaanxi, China; 481 Department of Medicine (DIMED), Surgical Pathology Unit, University of Padua, Padua, Italy; 482 Rigshospitalet, Copenhagen, Denmark; 483 Center for Cancer Genomics, National Cancer Institute, National Institutes of Health, Bethesda, MD USA; 484 Department of Biochemistry and Molecular Medicine, University of Montreal, Montreal, QC Canada; 485 Australian Institute of Tropical Health and Medicine, James Cook University, Douglas, QLD Australia; 486 Department of Neuro-Oncology, Istituto Neurologico Besta, Milano, Italy; 487 Bioplatforms Australia, North Ryde, NSW Australia; 488 Department of Pathology (Research), University College London Cancer Institute, London, UK; 489 Department of Surgical Oncology, Princess Margaret Cancer Centre, Toronto, ON Canada; 490 Department of Medical Oncology, Josephine Nefkens Institute and Cancer Genomics Centre, Erasmus Medical Center, Rotterdam, CN The Netherlands; 491 The University of Queensland Thoracic Research Centre, The Prince Charles Hospital, Brisbane, QLD Australia; 492 CIBIO/InBIO - Research Center in Biodiversity and Genetic Resources, Universidade do Porto, Vairão, Portugal; 493 HCA Laboratories, London, UK; 494 University of Liverpool, Liverpool, UK; 495 The Azrieli Faculty of Medicine, Bar-Ilan University, Safed, Israel; 496 Department of Neurosurgery, University of Florida, Gainesville, FL USA; 497 Department of Pathology, Graduate School of Medicine, University of Tokyo, Tokyo, Japan; 498 University of Milano Bicocca, Monza, Italy; 499 BGI-Shenzhen, Shenzhen, China; 500 Department of Pathology, Oslo University Hospital Ulleval, Oslo, Norway; 501 Center for Biomedical Informatics, Harvard Medical School, Boston, MA USA; 502 Department Biochemistry and Molecular Biomedicine, University of Barcelona, Barcelona, Spain; 503 Office of Cancer Genomics, National Cancer Institute, National Institutes of Health, Bethesda, MD USA; 504 Cancer Epigenomics, German Cancer Research Center (DKFZ), Heidelberg, Germany; 505 Department of Cancer Biology, The University of Texas MD Anderson Cancer Center, Houston, TX USA; 506 Department of Surgical Oncology, The University of Texas MD Anderson Cancer Center, Houston, TX USA; 507 Department of Computer Science, Yale University, New Haven, CT USA; 508 Department of Molecular Biophysics and Biochemistry, Yale University, New Haven, CT USA; 509 Program in Computational Biology and Bioinformatics, Yale University, New Haven, CT USA; 510 Center for Cancer Research, Massachusetts General Hospital, Boston, MA USA; 511 Department of Pathology, Massachusetts General Hospital, Boston, MA USA; 512 Department of Pathology, Memorial Sloan Kettering Cancer Center, New York, NY USA; 513 Division of Gastroenterology and Hepatology, Mayo Clinic, Rochester, MN USA; 514 University of Sydney, Sydney, NSW Australia; 515 University of Oxford, Oxford, UK; 516 Department of Surgery, Academic Urology Group, University of Cambridge, Cambridge, UK; 517 Department of Medicine II, University of Würzburg, Wuerzburg, Germany; 518 Sylvester Comprehensive Cancer Center, University of Miami, Miami, FL USA; 519 Institut Hospital del Mar d’Investigacions Mèdiques (IMIM), Barcelona, Spain; 520 Genome Integrity and Structural Biology Laboratory, National Institute of Environmental Health Sciences (NIEHS), Durham, NC USA; 521 St. Thomas’s Hospital, London, UK; 522 Osaka International Cancer Center, Osaka, Japan; 523 Department of Pathology, Skåne University Hospital, Lund University, Lund, Sweden; 524 Department of Medical Oncology, Beatson West of Scotland Cancer Centre, Glasgow, UK; 525 National Human Genome Research Institute, National Institutes of Health, Bethesda, MD USA; 526 Centre for Cancer Research, Victorian Comprehensive Cancer Centre, University of Melbourne, Melbourne, VIC Australia; 527 Department of Medicine, Section of Hematology/Oncology, University of Chicago, Chicago, IL USA; 528 German Center for Infection Research (DZIF), Partner Site Hamburg-Borstel-Lübeck-Riems, Hamburg, Germany; 529 Bioinformatics Research Centre (BiRC), Aarhus University, Aarhus, Denmark; 530 Department of Biotechnology, Ministry of Science and Technology, Government of India, New Delhi, Delhi India; 531 National Cancer Centre Singapore, Singapore, Singapore; 532 Brandeis University, Waltham, MA USA; 533 Department of Urologic Sciences, University of British Columbia, Vancouver, BC Canada; 534 Department of Internal Medicine, Stanford University, Stanford, CA USA; 535 The University of Texas Health Science Center at Houston, Houston, TX USA; 536 Imperial College NHS Trust, Imperial College, London, INY UK; 537 Senckenberg Institute of Pathology, University of Frankfurt Medical School, Frankfurt, Germany; 538 Department of Medicine, Division of Biomedical Informatics, UC San Diego School of Medicine, San Diego, CA USA; 539 Center for Precision Health, School of Biomedical Informatics, The University of Texas Health Science Center, Houston, TX USA; 540 Oxford Nanopore Technologies, New York, NY USA; 541 Institute of Medical Science, University of Tokyo, Tokyo, Japan; 542 Howard Hughes Medical Institute, University of California Santa Cruz, Santa Cruz, CA USA; 543 Wakayama Medical University, Wakayama, Japan; 544 Department of Internal Medicine, Division of Medical Oncology, Lineberger Comprehensive Cancer Center, University of North Carolina at Chapel Hill, Chapel Hill, NC USA; 545 University of Tennessee Health Science Center for Cancer Research, Memphis, TN USA; 546 Department of Histopathology, Salford Royal NHS Foundation Trust, Salford, UK; 547 Faculty of Biology, Medicine and Health, University of Manchester, Manchester, UK; 548 BIOPIC, ICG and College of Life Sciences, Peking University, Beijing, China; 549 Peking-Tsinghua Center for Life Sciences, Peking University, Beijing, China; 550 Children’s Hospital of Philadelphia, Philadelphia, PA USA; 551 Department of Bioinformatics and Computational Biology and Department of Systems Biology, The University of Texas MD Anderson Cancer Center, Houston, TX USA; 552 Karolinska Institute, Stockholm, Sweden; 553 The Donnelly Centre, University of Toronto, Toronto, ON Canada; 554 Department of Medical Genetics, College of Medicine, Hallym University, Chuncheon, South Korea; 555 Department of Experimental and Health Sciences, Institute of Evolutionary Biology (UPF-CSIC), Universitat Pompeu Fabra, Barcelona, Spain; 556 Health Data Science Unit, University Clinics, Heidelberg, Germany; 557 Massachusetts General Hospital Center for Cancer Research, Charlestown, MA USA; 558 Hokkaido University, Sapporo, Japan; 559 Department of Pathology and Clinical Laboratory, National Cancer Center Hospital, Tokyo, Japan; 560 Department of Genetics, University of North Carolina at Chapel Hill, Chapel Hill, NC USA; 561 Computational Biology, Leibniz Institute on Aging - Fritz Lipmann Institute (FLI), Jena, Germany; 562 University of Melbourne Centre for Cancer Research, Melbourne, VIC Australia; 563 University of Nebraska Medical Center, Omaha, NE USA; 564 Syntekabio Inc, Daejeon, South Korea; 565 Department of Pathology, Academic Medical Center, Amsterdam, AZ The Netherlands; 566 China National GeneBank-Shenzhen, Shenzhen, China; 567 Division of Molecular Genetics, German Cancer Research Center (DKFZ), Heidelberg, Germany; 568 Division of Life Science and Applied Genomics Center, Hong Kong University of Science and Technology, Clear Water Bay, Hong Kong, China; 569 Icahn School of Medicine at Mount Sinai, New York, NY USA; 570 Geneplus-Shenzhen, Shenzhen, China; 571 School of Computer Science and Technology, Xi’an Jiaotong University, Xi’an, China; 572 AbbVie, North Chicago, IL USA; 573 Institute of Pathology, Charité – University Medicine Berlin, Berlin, Germany; 574 Centre for Translational and Applied Genomics, British Columbia Cancer Agency, Vancouver, BC Canada; 575 Edinburgh Royal Infirmary, Edinburgh, UK; 576 Berlin Institute for Medical Systems Biology, Max Delbrück Center for Molecular Medicine, Berlin, Germany; 577 Department of Pediatric Immunology, Hematology and Oncology, University Hospital, Heidelberg, Germany; 578 German Cancer Research Center (DKFZ), Heidelberg, Germany; 579 Heidelberg Institute for Stem Cell Technology and Experimental Medicine (HI-STEM), Heidelberg, Germany; 580 Institute for Computational Biomedicine, Weill Cornell Medical College, New York, NY USA; 581 New York Genome Center, New York, NY USA; 582 Department of Urology, James Buchanan Brady Urological Institute, Johns Hopkins University School of Medicine, Baltimore, MD USA; 583 Department of Preventive Medicine, Graduate School of Medicine, The University of Tokyo, Tokyo, Japan; 584 Department of Molecular and Cellular Biology, Baylor College of Medicine, Houston, TX USA; 585 Department of Pathology and Immunology, Baylor College of Medicine, Houston, TX USA; 586 Michael E. DeBakey Veterans Affairs Medical Center, Houston, TX USA; 587 Technical University of Denmark, Lyngby, Denmark; 588 Department of Pathology, College of Medicine, Hanyang University, Seoul, South Korea; 589 Academic Unit of Surgery, School of Medicine, College of Medical, Veterinary and Life Sciences, University of Glasgow, Glasgow Royal Infirmary, Glasgow, UK; 590 Department of Pathology, Asan Medical Center, College of Medicine, Ulsan University, Songpa-gu, Seoul South Korea; 591 Science Writer, Garrett Park, MD USA; 592 International Cancer Genome Consortium (ICGC)/ICGC Accelerating Research in Genomic Oncology (ARGO) Secretariat, Ontario Institute for Cancer Research, Toronto, ON Canada; 593 University of Ljubljana, Ljubljana, Slovenia; 594 Department of Public Health Sciences, University of Chicago, Chicago, IL USA; 595 Research Institute, NorthShore University HealthSystem, Evanston, IL USA; 596 Department for Biomedical Research, University of Bern, Bern, Switzerland; 597 Centre of Genomics and Policy, McGill University and Génome Québec Innovation Centre, Montreal, QC Canada; 598 Carolina Center for Genome Sciences, University of North Carolina at Chapel Hill, Chapel Hill, NC USA; 599 Hopp Children’s Cancer Center (KiTZ), Heidelberg, Germany; 600 Pediatric Glioma Research Group, German Cancer Research Center (DKFZ), Heidelberg, Germany; 601 Cancer Research UK, London, UK; 602 Indivumed GmbH, Hamburg, Germany; 603 Genome Integration Data Center, Syntekabio, Inc, Daejeon, South Korea; 604 University Hospital Zurich, Zurich, Switzerland; 605 Clinical Bioinformatics, Swiss Institute of Bioinformatics, Geneva, Switzerland; 606 Institute for Pathology and Molecular Pathology, University Hospital Zurich, Zurich, Switzerland; 607 Institute of Molecular Life Sciences, University of Zurich, Zurich, Switzerland; 608 MRC Human Genetics Unit, MRC IGMM, University of Edinburgh, Edinburgh, UK; 609 Women’s Cancer Program at the Samuel Oschin Comprehensive Cancer Institute, Cedars-Sinai Medical Center, Los Angeles, CA USA; 610 Department of Biology, Bioinformatics Group, Division of Molecular Biology, Faculty of Science, University of Zagreb, Zagreb, Croatia; 611 Department for Internal Medicine II, University Hospital Schleswig-Holstein, Kiel, Germany; 612 Genetics and Molecular Pathology, SA Pathology, Adelaide, SA Australia; 613 Department of Gastric Surgery, National Cancer Center Hospital, Tokyo, Japan; 614 Department of Bioinformatics, Division of Cancer Genomics, National Cancer Center Research Institute, Tokyo, Japan; 615 A.A. Kharkevich Institute of Information Transmission Problems, Moscow, Russia; 616 Oncology and Immunology, Dmitry Rogachev National Research Center of Pediatric Hematology, Moscow, Russia; 617 Skolkovo Institute of Science and Technology, Moscow, Russia; 618 Department of Surgery, The George Washington University, School of Medicine and Health Science, Washington, DC USA; 619 Endocrine Oncology Branch, Center for Cancer Research, National Cancer Institute, National Institutes of Health, Bethesda, MD USA; 620 Melanoma Institute Australia, Macquarie University, Sydney, NSW Australia; 621 MIT Computer Science and Artificial Intelligence Laboratory, Massachusetts Institute of Technology, Cambridge, MA USA; 622 Tissue Pathology and Diagnostic Oncology, Royal Prince Alfred Hospital, Sydney, NSW Australia; 623 Cholangiocarcinoma Screening and Care Program and Liver Fluke and Cholangiocarcinoma Research Centre, Faculty of Medicine, Khon Kaen University, Khon Kaen, Thailand; 624 Controlled Department and Institution, New York, NY USA; 625 Englander Institute for Precision Medicine, Weill Cornell Medicine, New York, NY USA; 626 National Cancer Center, Gyeonggi, South Korea; 627 Department of Biochemistry, College of Medicine, Ewha Womans University, Seoul, South Korea; 628 Health Sciences Department of Biomedical Informatics, University of California San Diego, La Jolla, CA USA; 629 Research Core Center, National Cancer Centre Korea, Goyang-si, South Korea; 630 Department of Health Sciences and Technology, Sungkyunkwan University School of Medicine, Seoul, South Korea; 631 Samsung Genome Institute, Seoul, South Korea; 632 Breast Oncology Program, Dana-Farber/Brigham and Women’s Cancer Center, Boston, MA USA; 633 Department of Surgery, Memorial Sloan Kettering Cancer Center, New York, NY USA; 634 Division of Breast Surgery, Brigham and Women’s Hospital, Boston, MA USA; 635 Integrative Bioinformatics Support Group, National Institute of Environmental Health Sciences (NIEHS), Durham, NC USA; 636 Department of Clinical Science, University of Bergen, Bergen, Norway; 637 Center For Medical Innovation, Seoul National University Hospital, Seoul, South Korea; 638 Department of Internal Medicine, Seoul National University Hospital, Seoul, South Korea; 639 Institute of Computer Science, Polish Academy of Sciences, Warsawa, Poland; 640 Functional and Structural Genomics, German Cancer Research Center (DKFZ), Heidelberg, Germany; 641 Laboratory of Translational Genomics, Division of Cancer Epidemiology and Genetics, National Cancer Institute, , National Institutes of Health, Bethesda, MD USA; 642 Institute for Medical Informatics Statistics and Epidemiology, University of Leipzig, Leipzig, Germany; 643 Morgan Welch Inflammatory Breast Cancer Research Program and Clinic, The University of Texas MD Anderson Cancer Center, Houston, TX USA; 644 Department of Hematology and Oncology, Georg-Augusts-University of Göttingen, Göttingen, Germany; 645 Institute of Cell Biology (Cancer Research), University of Duisburg-Essen, Essen, Germany; 646 King’s College London and Guy’s and St. Thomas’ NHS Foundation Trust, London, UK; 647 Center for Epigenetics, Van Andel Research Institute, Grand Rapids, MI USA; 648 The University of Queensland Centre for Clinical Research, Royal Brisbane and Women’s Hospital, Herston, QLD Australia; 649 Department of Pediatric Oncology and Hematology, University of Cologne, Cologne, Germany; 650 University of Düsseldorf, Düsseldorf, Germany; 651 Department of Pathology, Institut Jules Bordet, Brussels, Belgium; 652 Institute of Biomedicine, Sahlgrenska Academy at University of Gothenburg, Gothenburg, Sweden; 653 Children’s Medical Research Institute, Sydney, NSW Australia; 654 ILSbio, LLC Biobank, Chestertown, MD USA; 655 Division of Genetics and Genomics, Boston Children’s Hospital, Harvard Medical School, Boston, MA USA; 656 Institute for Bioengineering and Biopharmaceutical Research (IBBR), Hanyang University, Seoul, South Korea; 657 Department of Statistics, University of California Santa Cruz, Santa Cruz, CA USA; 658 National Genotyping Center, Institute of Biomedical Sciences, Academia Sinica, Taipei, Taiwan; 659 Department of Vertebrate Genomics/Otto Warburg Laboratory Gene Regulation and Systems Biology of Cancer, Max Planck Institute for Molecular Genetics, Berlin, Germany; 660 McGill University and Genome Quebec Innovation Centre, Montreal, QC Canada; 661 biobyte solutions GmbH, Heidelberg, Germany; 662 Gynecologic Oncology, NYU Laura and Isaac Perlmutter Cancer Center, New York University, New York, NY USA; 663 Division of Oncology, Stem Cell Biology Section, Washington University School of Medicine, St. Louis, MO USA; 664 Department of Systems Biology, The University of Texas MD Anderson Cancer Center, Houston, TX USA; 665 Harvard University, Cambridge, MA USA; 666 Urologic Oncology Branch, Center for Cancer Research, National Cancer Institute, National Institutes of Health, Bethesda, MD USA; 667 University of Oslo, Oslo, Norway; 668 University of Toronto, Toronto, ON Canada; 669 Peking University, Beijing, China; 670 School of Life Sciences, Peking University, Beijing, China; 671 Leidos Biomedical Research, Inc, McLean, VA USA; 672 Hematology, Hospital Clinic, Institut d’Investigacions Biomèdiques August Pi i Sunyer (IDIBAPS), University of Barcelona, Barcelona, Spain; 673 Second Military Medical University, Shanghai, China; 674 Chinese Cancer Genome Consortium, Shenzhen, China; 675 Department of Medical Oncology, Beijing Hospital, Beijing, China; 676 Laboratory of Molecular Oncology, Key Laboratory of Carcinogenesis and Translational Research (Ministry of Education), Peking University Cancer Hospital and Institute, Beijing, China; 677 School of Medicine/School of Mathematics and Statistics, University of St. Andrews, St, Andrews, Fife UK; 678 Institute for Systems Biology, Seattle, WA USA; 679 Department of Biochemistry and Molecular Biology, Faculty of Medicine, University Institute of Oncology-IUOPA, Oviedo, Spain; 680 Institut Bergonié, Bordeaux, France; 681 Cancer Unit, MRC University of Cambridge, Cambridge, UK; 682 Department of Pathology and Laboratory Medicine, Center for Personalized Medicine, Children’s Hospital Los Angeles, Los Angeles, CA USA; 683 John Curtin School of Medical Research, Canberra, ACT Australia; 684 MVZ Department of Oncology, PraxisClinic am Johannisplatz, Leipzig, Germany; 685 Department of Information Technology, Ghent University, Ghent, Belgium; 686 Department of Plant Biotechnology and Bioinformatics, Ghent University, Ghent, Belgium; 687 Institute for Genomic Medicine, Nationwide Children’s Hospital, Columbus, OH USA; 688 Computational Biology Program, School of Medicine, Oregon Health and Science University, Portland, OR USA; 689 Department of Surgery, Duke University, Durham, NC USA; 690 Institució Catalana de Recerca i Estudis Avançats (ICREA), Barcelona, Spain; 691 Institut Català de Paleontologia Miquel Crusafont, Universitat Autònoma de Barcelona, Barcelona, Spain; 692 University of Glasgow, Glasgow, UK; 693 Institut d’Investigacions Biomèdiques August Pi i Sunyer (IDIBAPS), Barcelona, Spain; 694 Division of Oncology, Washington University School of Medicine, St. Louis, MO USA; 695 Department of Surgery and Cancer, Imperial College, London, INY UK; 696 Applications Department, Oxford Nanopore Technologies, Oxford, UK; 697 Department of Obstetrics, Gynecology and Reproductive Services, University of California San Francisco, San Francisco, CA USA; 698 Department of Biochemistry and Molecular Medicine, University California at Davis, Sacramento, CA USA; 699 STTARR Innovation Facility, Princess Margaret Cancer Centre, Toronto, ON Canada; 700 Discipline of Surgery, Western Sydney University, Penrith, NSW Australia; 701 Yale School of Medicine, Yale University, New Haven, CT USA; 702 Department of Genetics, Lineberger Comprehensive Cancer Center, University of North Carolina at Chapel Hill, Chapel Hill, NC USA; 703 Departments of Neurology and Neurosurgery, Henry Ford Hospital, Detroit, MI USA; 704 Precision Oncology, OHSU Knight Cancer Institute, Oregon Health and Science University, Portland, OR USA; 705 Institute of Pathology, University Medical Center Hamburg-Eppendorf, Hamburg, Germany; 706 Department of Health Sciences, Faculty of Medical Sciences, Kyushu University, Fukuoka, Japan; 707 Heidelberg Academy of Sciences and Humanities, Heidelberg, Germany; 708 Department of Clinical Pathology, University of Melbourne, Melbourne, VIC, Australia; 709 Department of Pathology, Roswell Park Cancer Institute, Buffalo, NY USA; 710 Department of Computer Science, University of Helsinki, Helsinki, Finland; 711 Institute of Biotechnology, University of Helsinki, Helsinki, Finland; 712 Organismal and Evolutionary Biology Research Programme, University of Helsinki, Helsinki, Finland; 713 Department of Obstetrics and Gynecology, Division of Gynecologic Oncology, Washington University School of Medicine, St. Louis, MO USA; 714 Penrose St. Francis Health Services, Colorado Springs, CO USA; 715 Institute of Pathology, Ulm University and University Hospital of Ulm, Ulm, Germany; 716 National Cancer Center, Tokyo, Japan; 717 Genome Institute of Singapore, Singapore, Singapore; 718 32Program in Computational Biology and Bioinformatics, Yale University, New Haven, CT USA; 719 German Cancer Aid, Bonn, Germany; 720 Programme in Cancer and Stem Cell Biology, Centre for Computational Biology, Duke-NUS Medical School, Singapore, Singapore; 721 The Chinese University of Hong Kong, Shatin, NT, Hong Kong China; 722 Fourth Military Medical University, Shaanxi, China; 723 The University of Cambridge School of Clinical Medicine, Cambridge, UK; 724 St. Jude Children’s Research Hospital, Memphis, TN USA; 725 University Health Network, Princess Margaret Cancer Centre, Toronto, ON Canada; 726 Center for Biomolecular Science and Engineering, University of California Santa Cruz, Santa Cruz, CA USA; 727 Department of Medicine, University of Chicago, Chicago, IL USA; 728 Department of Neurology, Mayo Clinic, Rochester, MN USA; 729 Cambridge Oesophagogastric Centre, Cambridge University Hospitals NHS Foundation Trust, Cambridge, UK; 730 Department of Computer Science, Carleton College, Northfield, MN USA; 731 Institute of Cancer Sciences, College of Medical Veterinary and Life Sciences, University of Glasgow, Glasgow, UK; 732 Department of Epidemiology, University of Alabama at Birmingham, Birmingham, AL USA; 733 HudsonAlpha Institute for Biotechnology, Huntsville, AL USA; 734 O’Neal Comprehensive Cancer Center, University of Alabama at Birmingham, Birmingham, AL USA; 735 Department of Pathology, Keio University School of Medicine, Tokyo, Japan; 736 Department of Hepatobiliary and Pancreatic Oncology, National Cancer Center Hospital, Tokyo, Japan; 737 Sage Bionetworks, Seattle, WA USA; 738 Lymphoma Genomic Translational Research Laboratory, National Cancer Centre, Singapore, Singapore; 739 Department of Clinical Pathology, Robert-Bosch-Hospital, Stuttgart, Germany; 740 Department of Cell and Systems Biology, University of Toronto, Toronto, ON Canada; 741 Department of Biosciences and Nutrition, Karolinska Institutet, Stockholm, Sweden; 742 Center for Liver Cancer, Research Institute and Hospital, National Cancer Center, Gyeonggi, South Korea; 743 Division of Hematology-Oncology, Samsung Medical Center, Sungkyunkwan University School of Medicine, Seoul, South Korea; 744 Samsung Advanced Institute for Health Sciences and Technology, Sungkyunkwan University School of Medicine, Seoul, South Korea; 745 Cheonan Industry-Academic Collaboration Foundation, Sangmyung University, Cheonan, South Korea; 746 NYU Langone Medical Center, New York, NY USA; 747 Department of Hematology and Medical Oncology, Cleveland Clinic, Cleveland, OH USA; 748 Department of Radiation Oncology, University of California San Francisco, San Francisco, CA USA; 749 Department of Health Sciences Research, Mayo Clinic, Rochester, MN USA; 750 Helen F. Graham Cancer Center at Christiana Care Health Systems, Newark, DE USA; 751 Heidelberg University Hospital, Heidelberg, Germany; 752 CSRA Incorporated, Fairfax, VA USA; 753 Research Department of Pathology, University College London Cancer Institute, London, UK; 754 Department of Research Oncology, Guy’s Hospital, King’s Health Partners AHSC, King’s College London School of Medicine, London, UK; 755 Faculty of Medicine and Health Sciences, Macquarie University, Sydney, NSW Australia; 756 University Hospital of Minjoz, INSERM UMR 1098, Besançon, France; 757 Spanish National Cancer Research Centre, Madrid, Spain; 758 Center of Digestive Diseases and Liver Transplantation, Fundeni Clinical Institute, Bucharest, Romania; 759 Cureline, Inc, South San Francisco, CA USA; 760 St. Luke’s Cancer Centre, Royal Surrey County Hospital NHS Foundation Trust, Guildford, UK; 761 Cambridge Breast Unit, Addenbrooke’s Hospital, Cambridge University Hospital NHS Foundation Trust and NIHR Cambridge Biomedical Research Centre, Cambridge, UK; 762 East of Scotland Breast Service, Ninewells Hospital, Aberdeen, UK; 763 Department of Genetics, Microbiology and Statistics, University of Barcelona, IRSJD, IBUB, Barcelona, Spain; 764 Department of Obstetrics and Gynecology, Medical College of Wisconsin, Milwaukee, WI USA; 765 Hematology and Medical Oncology, Winship Cancer Institute of Emory University, Atlanta, GA USA; 766 Department of Computer Science, Princeton University, Princeton, NJ USA; 767 Vanderbilt Ingram Cancer Center, Vanderbilt University, Nashville, TN USA; 768 Ohio State University College of Medicine and Arthur G. James Comprehensive Cancer Center, Columbus, OH USA; 769 Department of Surgery, Yokohama City University Graduate School of Medicine, Kanagawa, Japan; 770 Division of Chromatin Networks, German Cancer Research Center (DKFZ) and BioQuant, Heidelberg, Germany; 771 Research Computing Center, University of North Carolina at Chapel Hill, Chapel Hill, NC USA; 772 School of Molecular Biosciences and Center for Reproductive Biology, Washington State University, Pullman, WA USA; 773 Finsen Laboratory and Biotech Research and Innovation Centre (BRIC), University of Copenhagen, Copenhagen, Denmark; 774 Department of Laboratory Medicine and Pathobiology, University of Toronto, Toronto, ON Canada; 775 Department of Pathology, Human Oncology and Pathogenesis Program, Memorial Sloan Kettering Cancer Center, New York, NY USA; 776 University Hospital Giessen, Pediatric Hematology and Oncology, Giessen, Germany; 777 Oncologie Sénologie, ICM Institut Régional du Cancer, Montpellier, France; 778 Institute of Clinical Molecular Biology, Christian-Albrechts-University, Kiel, Germany; 779 Institute of Pathology, University of Wuerzburg, Wuerzburg, Germany; 780 Department of Urology, North Bristol NHS Trust, Bristol, UK; 781 SingHealth, Duke-NUS Institute of Precision Medicine, National Heart Centre Singapore, Singapore, Singapore; 782 Department of Computer Science, University of Toronto, Toronto, ON Canada; 783 Bern Center for Precision Medicine, University Hospital of Bern, University of Bern, Bern, Switzerland; 784 Englander Institute for Precision Medicine, Weill Cornell Medicine and New York Presbyterian Hospital, New York, NY USA; 785 Meyer Cancer Center, Weill Cornell Medicine, New York, NY USA; 786 Pathology and Laboratory, Weill Cornell Medical College, New York, NY USA; 787 Vall d’Hebron Institute of Oncology: VHIO, Barcelona, Spain; 788 General and Hepatobiliary-Biliary Surgery, Pancreas Institute, University and Hospital Trust of Verona, Verona, Italy; 789 National Centre for Biological Sciences, Tata Institute of Fundamental Research, Bangalore, India; 790 Indiana University, Bloomington, IN USA; 791 Department of Pathology, GZA-ZNA Hospitals, Antwerp, Belgium; 792 Analytical Biological Services, Inc, Wilmington, DE USA; 793 Sydney Medical School, University of Sydney, Sydney, NSW Australia; 794 cBio Center, Dana-Farber Cancer Institute, Harvard Medical School, Boston, MA USA; 795 Department of Cell Biology, Harvard Medical School, Boston, MA USA; 796 Advanced Centre for Treatment Research and Education in Cancer, Tata Memorial Centre, Navi Mumbai, Maharashtra India; 797 School of Environmental and Life Sciences, Faculty of Science, The University of Newcastle, Ourimbah, NSW Australia; 798 Department of Dermatology, University Hospital of Essen, Essen, Germany; 799 Bioinformatics and Omics Data Analytics, German Cancer Research Center (DKFZ), Heidelberg, Germany; 800 Department of Urology, Charité Universitätsmedizin Berlin, Berlin, Germany; 801 Martini-Clinic, Prostate Cancer Center, University Medical Center Hamburg-Eppendorf, Hamburg, Germany; 802 Department of General Internal Medicine, University of Kiel, Kiel, Germany; 803 German Cancer Consortium (DKTK), Partner site Berlin, Berlin, Germany; 804 Cancer Research Institute, Beth Israel Deaconess Medical Center, Boston, MA USA; 805 University of Pittsburgh, Pittsburgh, PA USA; 806 Department of Ophthalmology and Ocular Genomics Institute, Massachusetts Eye and Ear, Harvard Medical School, Boston, MA USA; 807 Center for Psychiatric Genetics, NorthShore University HealthSystem, Evanston, IL USA; 808 Van Andel Research Institute, Grand Rapids, MI USA; 809 Laboratory of Molecular Medicine, Human Genome Center, Institute of Medical Science, University of Tokyo, Tokyo, Japan; 810 Japan Agency for Medical Research and Development, Tokyo, Japan; 811 Korea University, Seoul, South Korea; 812 Murtha Cancer Center, Walter Reed National Military Medical Center, Bethesda, MD USA; 813 Human Genetics, University of Kiel, Kiel, Germany; 814 Department of Oncologic Pathology, Dana-Farber Cancer Institute, Harvard Medical School, Boston, MA USA; 815 Oregon Health and Science University, Portland, OR USA; 816 Center for RNA Interference and Noncoding RNA, The University of Texas MD Anderson Cancer Center, Houston, TX USA; 817 Department of Experimental Therapeutics, The University of Texas MD Anderson Cancer Center, Houston, TX USA; 818 Department of Gynecologic Oncology and Reproductive Medicine, The University of Texas MD Anderson Cancer Center, Houston, TX USA; 819 University Hospitals Coventry and Warwickshire NHS Trust, Coventry, UK; 820 Department of Radiation Oncology, Radboud University Nijmegen Medical Centre, Nijmegen, GA The Netherlands; 821 Institute for Genomics and Systems Biology, University of Chicago, Chicago, IL USA; 822 Clinic for Hematology and Oncology, St.-Antonius-Hospital, Eschweiler, Germany; 823 Computational and Systems Biology Program, Memorial Sloan Kettering Cancer Center, New York, NY USA; 824 University of Iceland, Reykjavik, Iceland; 825 Division of Computational Genomics and Systems Genetics, German Cancer Research Center (DKFZ), Heidelberg, Germany; 826 Dundee Cancer Centre, Ninewells Hospital, Dundee, UK; 827 Department for Internal Medicine III, University of Ulm and University Hospital of Ulm, Ulm, Germany; 828 Institut Curie, INSERM Unit 830, Paris, France; 829 Department of Gastroenterology and Hepatology, Yokohama City University Graduate School of Medicine, Kanagawa, Japan; 830 Department of Laboratory Medicine, Radboud University Nijmegen Medical Centre, Nijmegen, GA The Netherlands; 831 Division of Cancer Genome Research, German Cancer Research Center (DKFZ), Heidelberg, Germany; 832 Department of General Surgery, Singapore General Hospital, Singapore, Singapore; 833 Cancer Science Institute of Singapore, National University of Singapore, Singapore, Singapore; 834 Department of Medical and Clinical Genetics, Genome-Scale Biology Research Program, University of Helsinki, Helsinki, Finland; 835 East Anglian Medical Genetics Service, Cambridge University Hospitals NHS Foundation Trust, Cambridge, UK; 836 Irving Institute for Cancer Dynamics, Columbia University, New York, NY USA; 837 Institute of Molecular and Cell Biology, Singapore, Singapore; 838 Laboratory of Cancer Epigenome, Division of Medical Science, National Cancer Centre Singapore, Singapore, Singapore; 839 Universite Lyon, INCa-Synergie, Centre Léon Bérard, Lyon, France; 840 Department of Urology, Mayo Clinic, Rochester, MN USA; 841 Royal National Orthopaedic Hospital - Stanmore, Stanmore, Middlesex UK; 842 Department of Biochemistry, Genetics and Immunology, University of Vigo, Vigo, Spain; 843 Giovanni Paolo II / I.R.C.C.S. Cancer Institute, Bari, BA Italy; 844 Neuroblastoma Genomics, German Cancer Research Center (DKFZ), Heidelberg, Germany; 845 Fondazione Policlinico Universitario Gemelli IRCCS, Rome, Italy, Rome, Italy; 846 University of Verona, Verona, Italy; 847 Centre National de Génotypage, CEA - Institute de Génomique, Evry, France; 848 CAPHRI Research School, Maastricht University, Maastricht, ER The Netherlands; 849 Department of Biopathology, Centre Léon Bérard, Lyon, France; 850 Université Claude Bernard Lyon 1, Villeurbanne, France; 851 Core Research for Evolutional Science and Technology (CREST), JST, Tokyo, Japan; 852 Department of Biological Sciences, Laboratory for Medical Science Mathematics, Graduate School of Science, University of Tokyo, Yokohama, Japan; 853 Department of Medical Science Mathematics, Medical Research Institute, Tokyo Medical and Dental University (TMDU), Tokyo, Japan; 854 Cancer Ageing and Somatic Mutation Programme, Wellcome Sanger Institute, Hinxton, UK; 855 University Hospitals Birmingham NHS Foundation Trust, Birmingham, UK; 856 Centre for Cancer Research and Cell Biology, Queen’s University, Belfast, UK; 857 Breast Medical Oncology, The University of Texas MD Anderson Cancer Center, Houston, TX USA; 858 Department of Surgery, Johns Hopkins University School of Medicine, Baltimore, MD USA; 859 Department of Oncology-Pathology, Science for Life Laboratory, Karolinska Institute, Stockholm, Sweden; 860 School of Cancer Sciences, Faculty of Medicine, University of Southampton, Southampton, UK; 861 Department of Gene Technology, Tallinn University of Technology, Tallinn, Estonia; 862 Genetics and Genome Biology Program, SickKids Research Institute, The Hospital for Sick Children, Toronto, ON Canada; 863 Departments of Neurosurgery and Hematology and Medical Oncology, Winship Cancer Institute and School of Medicine, Emory University, Atlanta, GA USA; 864 Department of Clinical and Molecular Medicine, Faculty of Medicine and Health Sciences, Norwegian University of Science and Technology, Trondheim, Norway; 865 Argmix Consulting, North Vancouver, BC Canada; 866 Department of Information Technology, Ghent University, Interuniversitair Micro-Electronica Centrum (IMEC), Ghent, Belgium; 867 Nuffield Department of Surgical Sciences, John Radcliffe Hospital, University of Oxford, Oxford, UK; 868 Institute of Mathematics and Computer Science, University of Latvia, Riga, LV Latvia; 869 Discipline of Pathology, Sydney Medical School, University of Sydney, Sydney, NSW Australia; 870 Department of Applied Mathematics and Theoretical Physics, Centre for Mathematical Sciences, University of Cambridge, Cambridge, UK; 871 Department of Epidemiology and Biostatistics, Memorial Sloan Kettering Cancer Center, New York, NY USA; 872 Department of Statistics, Columbia University, New York, NY USA; 873 Department of Immunology, Genetics and Pathology, Science for Life Laboratory, Uppsala University, Uppsala, Sweden; 874 School of Electronic and Information Engineering, Xi’an Jiaotong University, Xi’an, China; 875 Department of Histopathology, Cambridge University Hospitals NHS Foundation Trust, Cambridge, UK; 876 Oxford NIHR Biomedical Research Centre, University of Oxford, Oxford, UK; 877 Georgia Regents University Cancer Center, Augusta, GA USA; 878 Wythenshawe Hospital, Manchester, UK; 879 Department of Genetics, Washington University School of Medicine, St.Louis, MO USA; 880 Department of Biological Oceanography, Leibniz Institute of Baltic Sea Research, Rostock, Germany; 881 Wellcome Centre for Human Genetics, University of Oxford, Oxford, UK; 882 Department of Molecular and Human Genetics, Baylor College of Medicine, Houston, TX USA; 883 Thoracic Oncology Laboratory, Mayo Clinic, Rochester, MN USA; 884 Institute for Genomic Medicine, Nationwide Children’s Hospital, Columbus, OH USA; 885 Department of Obstetrics and Gynecology, Division of Gynecologic Oncology, Mayo Clinic, Rochester, MN USA; 886 International Institute for Molecular Oncology, Poznań, Poland; 887 Poznan University of Medical Sciences, Poznań, Poland; 888 Genomics and Proteomics Core Facility High Throughput Sequencing Unit, German Cancer Research Center (DKFZ), Heidelberg, Germany; 889 NCCS-VARI Translational Research Laboratory, National Cancer Centre Singapore, Singapore, Singapore; 890 Edison Family Center for Genome Sciences and Systems Biology, Washington University, St. Louis, MO USA; 891 MRC-University of Glasgow Centre for Virus Research, Glasgow, UK; 892 Department of Medical Informatics and Clinical Epidemiology, Division of Bioinformatics and Computational Biology, OHSU Knight Cancer Institute, Oregon Health and Science University, Portland, OR USA; 893 School of Electronic Information and Communications, Huazhong University of Science and Technology, Wuhan, China; 894 Department of Applied Mathematics and Statistics, Johns Hopkins University, Baltimore, MD USA; 895 Department of Cancer Genome Informatics, Graduate School of Medicine, Osaka University, Osaka, Japan; 896 Institute of Computer Science, Heidelberg University, Heidelberg, Germany; 897 School of Mathematics and Statistics, University of Sydney, Sydney, NSW Australia; 898 Ben May Department for Cancer Research, University of Chicago, Chicago, IL USA; 899 Department of Human Genetics, University of Chicago, Chicago, IL USA; 900 Tri-Institutional PhD Program in Computational Biology and Medicine, Weill Cornell Medicine, New York, NY USA; 901 The First Affiliated Hospital, Xi’an Jiaotong University, Xi’an, China; 902 Department of Medicine and Therapeutics, The Chinese University of Hong Kong, Shatin, NT, Hong Kong China; 903 Department of Biostatistics, The University of Texas MD Anderson Cancer Center, Houston, TX USA; 904 Duke-NUS Medical School, Singapore, Singapore; 905 Department of Surgery, Ruijin Hospital, Shanghai Jiaotong University School of Medicine, Shanghai, China; 906 School of Computing Science, University of Glasgow, Glasgow, UK; 907 Division of Orthopaedic Surgery, Oslo University Hospital, Oslo, Norway; 908 Eastern Clinical School, Monash University, Melbourne, VIC Australia; 909 Epworth HealthCare, Richmond, VIC Australia; 910 Department of Biostatistics and Computational Biology, Dana-Farber Cancer Institute and Harvard Medical School, Boston, MA USA; 911 Department of Biomedical Informatics, College of Medicine, The Ohio State University, Columbus, OH USA; 912 The Ohio State University Comprehensive Cancer Center (OSUCCC – James), Columbus, OH USA; 913 The University of Texas School of Biomedical Informatics (SBMI) at Houston, Houston, TX USA; 914 Department of Biostatistics, University of North Carolina at Chapel Hill, Chapel Hill, NC USA; 915 Department of Biochemistry and Molecular Genetics, Feinberg School of Medicine, Northwestern University, Chicago, IL USA; 916 Faculty of Medicine and Health, University of Sydney, Sydney, NSW Australia; 917 Department of Pathology, Erasmus Medical Center Rotterdam, Rotterdam, GD The Netherlands; 918 Division of Molecular Carcinogenesis, The Netherlands Cancer Institute, Amsterdam, CX The Netherlands; 919 Institute of Molecular Life Sciences and Swiss Institute of Bioinformatics, University of Zurich, Zurich, Switzerland

**Keywords:** Cancer, Genomics

## Abstract

Mitochondria are essential cellular organelles that play critical roles in cancer. Here, as part of the International Cancer Genome Consortium/The Cancer Genome Atlas Pan-Cancer Analysis of Whole Genomes Consortium, which aggregated whole-genome sequencing data from 2,658 cancers across 38 tumor types, we performed a multidimensional, integrated characterization of mitochondrial genomes and related RNA sequencing data. Our analysis presents the most definitive mutational landscape of mitochondrial genomes and identifies several hypermutated cases. Truncating mutations are markedly enriched in kidney, colorectal and thyroid cancers, suggesting oncogenic effects with the activation of signaling pathways. We find frequent somatic nuclear transfers of mitochondrial DNA, some of which disrupt therapeutic target genes. Mitochondrial copy number varies greatly within and across cancers and correlates with clinical variables. Co-expression analysis highlights the function of mitochondrial genes in oxidative phosphorylation, DNA repair and the cell cycle, and shows their connections with clinically actionable genes. Our study lays a foundation for translating mitochondrial biology into clinical applications.

## Abstract (web summary)

Analysis of mitochondrial genomes (mtDNA) by using whole-genome sequencing data from 2,658 cancer samples across 38 cancer types identifies hypermutated mtDNA cases, frequent somatic nuclear transfer of mtDNA and high variability of mtDNA copy number in many cancers.

## Main

Mitochondria are crucial cellular organelles in eukaryotes, and there can be several hundred mitochondria in a single human cell^1^. Known as ‘the powerhouses of the cell’, mitochondria play essential roles in generating most of the cell’s energy through oxidative phosphorylation^2^. Despite its small size (16.6 kilobases (kb)), the circular mitochondrial genome encodes 13 proteins that form respiratory chain complexes with other proteins of nuclear origin^3^. The involvement of mitochondria in carcinogenesis has long been suspected^4,5^ because altered energy metabolism is a common feature of cancer^6^. Furthermore, mitochondria play important roles in other tasks, such as biosynthesis, signaling, cellular differentiation, apoptosis, maintaining control of the cell cycle and cell growth, all of which are intrinsically linked to tumorigenesis^5,7^.

In several recent studies, molecular characterization of mitochondria was performed in cancer by using next-generation sequencing data^8–13^, but these studies usually describe one specific dimension of the mitochondrial genome (for example, somatic mutations) based on relatively small sample cohorts. Furthermore, due to the whole-exome sequencing data employed, the relatively low depth of mitochondrial genomes limits the accuracy and scope of these studies. Thus, a comprehensive, multidimensional molecular portrait of mitochondria across a broad range of cancer types has not been achieved. Moreover, previous studies have focused on the patterns of mitochondrial alterations alone, without fully exploring the interplay between the mitochondrial genome and the nuclear genome, as well as the biomedical significance of mitochondrial alterations.

**Fig. 1:**
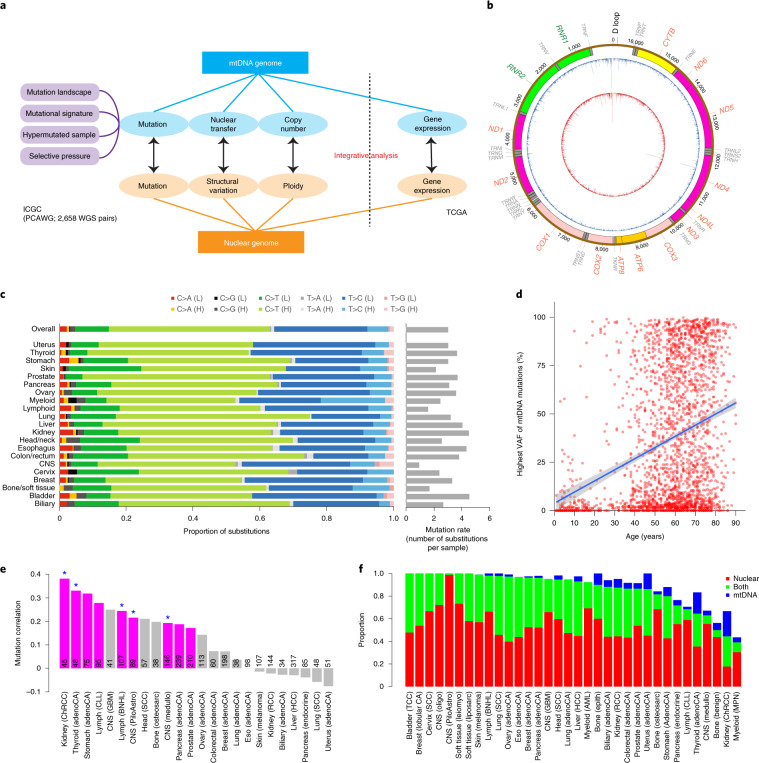
Mutational landscape and process of cancer mitochondrial genomes. **a**, Overview of our multidimensional and integrated mitochondrial genome analyses. **b**, Landscape of mtDNA somatic substitutions. The numbers represent the mitochondrial genome coordinates. The outer (blue) circle shows the density of all variants with VAF > 1%. The inner (red) circle shows the density of variants with VAF > 3%. **c**, Highly consistent mtDNA mutational spectrum across 21 cancer tissue groups. Average numbers of somatic substitutions per sample are also shown (right). **d**, Correlation between the highest VAF of mtDNA mutations in a cancer tissue and patient age at the time of diagnosis. The correlation was based on the 2,414 patients with both age and somatic single-nucleotide variant information available. The shaded region represents the 95% confidence interval for the predictions from a linear model with the highest VAF as the response variable and patient age as the explanatory variable. **e**, Correlations between the numbers of nuclear and mtDNA somatic mutations. The associations were tested among samples with both nuclear and mtDNA somatic mutations available (with sample sizes labeled on plot) using Spearman’s rank correlation. Magenta bars indicate significant positive correlations (*P* < 0.05). Asterisks indicate that both nuclear and mitochondrial somatic mutations were correlated with patient age in that cancer type (*P* < 0.05). **f**, Proportions of tumor samples harboring different categories of somatic alterations: nuclear driver alterations only (red); both nuclear driver alterations and mtDNA mutations (VAF > 10%, green); and mtDNA mutations without known nuclear drivers (blue). AML, acute myeloid leukemia; BNHL, B cell non-Hodgkin lymphoma; CA, carcinoma; ChRCC, chromophobe renal cell carcinoma; CLL, chronic lymphocytic leukemia; CNS, central nervous system; eso, esophageal; GBM, glioblastoma; HCC, hepatocellular carcinoma; leiomyo, leiomyosarcoma; liposarc, liposarcoma; medullo, medulloblastoma; MPN, myeloproliferative neoplasm; oligo, oligometastatic; osteosarc, osteosarcoma; PiloAstro, pilocytic astrocytoma; RCC, renal cell carcinoma; SCC, squamous cell carcinoma; TCC, transitional cell carcinoma; adenoCA, adenocarcinoma; epith, epithelioid.

The Pan-Cancer Analysis of Whole Genomes (PCAWG) Consortium aggregated whole-genome sequencing (WGS) data from 2,658 cancers across 38 tumor types generated by the International Cancer Genome Consortium (ICGC) and The Cancer Genome Atlas (TCGA) projects. These sequencing data were re-analyzed with standardized, high-accuracy pipelines to align to the human genome (reference build hs37d5) and identify germline variants and somatically acquired mutations, as described^14^. Meanwhile, TCGA has generated RNA sequencing (RNA-seq) data from a large number of patient samples, which allow for assessment of the transcriptional activities of mitochondrial genes^15^. These large-scale datasets create a tremendous resource for characterizing cancer mitochondrial genomes at an unprecedented level (Fig. 1a). We first characterized mitochondrial somatic mutations, nuclear transfers and copy numbers, then investigated their interactions with nuclear somatic alterations^16,17^, and finally examined the expression profiles of mitochondrial genes and their connections with clinically relevant nuclear genes.

## Results

### Mutational landscape of cancer mitochondrial genomes

To characterize somatic mutations in mitochondrial genomes across cancer types, we extracted the mitochondrial DNA (mtDNA) mapped reads of 2,658 cancer and matched control sample pairs from the PCAWG Consortium. The samples we surveyed covered 21 cancer tissues and 38 specific cancer types (Supplementary Table 1). On average, the sequencing depth for the mitochondrial genome was 9,959×, which was much higher than that obtained from whole-exome sequencing data, allowing for confident detection of somatic mutations at a very low heteroplasmic level (variant allele fraction (VAF) > 1%; Supplementary Fig. 1). By applying a well-designed computational pipeline that carefully considered various potentially confounding factors (for example, sample cross-contamination, mismapping of reads from nuclear mtDNA-like sequence^18^, and artifactual mutations caused by oxidative DNA damage during library preparation^19^), we identified a total of 7,611 somatic substitutions and 930 small indels in 2,536 high-quality cancer samples (122 samples were excluded in the mutation analysis for the issues mentioned above; Supplementary Fig. 2 and Methods). The high reliability of the mutations was confirmed by long-range PCR-based validation (Supplementary Table 2) and by inspection of the mutational spectrum of the very low-VAF mutation candidates (Supplementary Fig. 3).

Of the 7,611 substitutions, >85% were clearly heteroplasmic, showing VAFs lower than 0.6 (average: 0.2; median: 0.045). Overall, mtDNA mutations located in the transcribed regions were also found in RNA-seq with similar VAFs, except for a fraction of transfer RNA (tRNA) mutations showing much higher VAFs in transcripts due to the accumulation of unprocessed tRNA precursors during the processing of polycistronic mitochondrial transcripts^10^ (Supplementary Fig. 4). Across all of the cancer samples, we observed several mutational hotspots in the regulatory D-loop region and the *ND4* gene (Fig. 1b). Of the 13 protein-coding genes, *ND5* was the most frequently mutated in most cancer types, while *ND4* was most frequently mutated in prostate and lung cancers, and *COX1* was most frequently mutated in breast, cervical and bladder cancers (Supplementary Fig. 5). We identified that cancer type and gene identity were associated with the mutation status of the 13 coding genes (log-linear model, *P*_cancer type_ < 2.2 × 10^−16^; *P*_gene_ < 2.2 × 10^−16^), but the effect of their interaction was not significant (*P*_cancer type × gene_ = 0.12).

In contrast with somatic mutations in nuclear genomes (where cancer type-specific mutational signatures are observed)^20^, mtDNA mutational signatures were very similar across tumor types, with C:G>T:A (58.3%) and T:A>C:G (34.2%) substitutions being the most and second most frequent mutation types, respectively (Fig. 1c and Supplementary Fig. 6). Indeed, the impact of well-known carcinogens (for example, tobacco smoking (C:G>A:T dominant; signature 4), ultraviolet light (C:G>T:A dominant at dipyrimidine contexts; signature 7) and reactive oxygen species (G:C>T:A dominant)) were minimal (Supplementary Fig. 7) even in lung and skin cancers (the latest mutational signatures of nuclear genomes are available from the Catalogue of Somatic Mutations in Cancer database: https://cancer.sanger.ac.uk/cosmic/signatures). Instead, the vast majority of mtDNA mutations were manifesting extreme replicational mtDNA strand bias^9,21,22^; that is, predominant G>A and T>C substitutions and deficient complementary C>T and A>G substitutions on the light (L) strand of the mtDNA genome sequence (+strand of the revised Cambridge Reference Sequence) despite the relative depletion of guanines and thymines on the L strand (Supplementary Fig. 6). These mutational signatures suggest that mitochondria-specific, replication-coupled mutational processes (such as mtDNA polymerase gamma error^9,21,23^ or other replication-coupled DNA damage mechanisms) are dominantly responsible for somatic mtDNA mutations in cancer.

In agreement with its endogenous origin, we observed clock-like properties in mtDNA mutations as nuclear genome mutations^24^. The number of mtDNA mutations in our study was largely proportional to the age of the patient at the time of tissue sampling (Supplementary Fig. 8). In addition, the maximum VAF of somatic mutations in a cancer tissue showed positive correlation with age, on average (Fig. 1d; *P* < 2.2 × 10^−16^). Collectively, these results suggest that the vast majority of mtDNA somatic mutations were: (1) acquired at an earlier age when the cell lineage was phenotypically normal; and (2) overall shifted towards homoplasmy throughout life in the cellular lineage of the neoplastic cells. The spread to homoplasmy can, in theory, be caused by either physiological advantage (selection) or a series of asymmetric segregations during cell divisions (drift)^25^, or both.

To further assess the potential impact of mtDNA mutations, we performed integrative analysis by examining alterations from mitochondrial and nuclear genomes simultaneously^17^. We observed significantly positive correlations between the mutation burdens of mitochondrial and nuclear genomes in several cancer types, with the highest correlations observed in kidney chromophobe and thyroid cancers (magenta bars in Fig. 1e). Some of these correlations may be explained by the age effect, as the mutation numbers in both mitochondrial and nuclear genomes were significantly correlated with patient age in the corresponding cancer types (bars marked with an asterisk in Fig. 1e). In addition, we examined the mtDNA mutation frequency in the context of nuclear drivers. Although nuclear driver alterations exist in the majority of patients in most cancer types, a notable proportion of patients (22.2% with kidney chromophobe cancer and 18.8% with thyroid cancer) bear non-silent mtDNA mutations but no known nuclear drivers, suggesting a potential functional contribution of mtDNA mutations in the absence of nuclear drivers in these cancer types (Fig. 1f).

### Hypermutation process in mitochondrial genomes

**Fig. 2:**
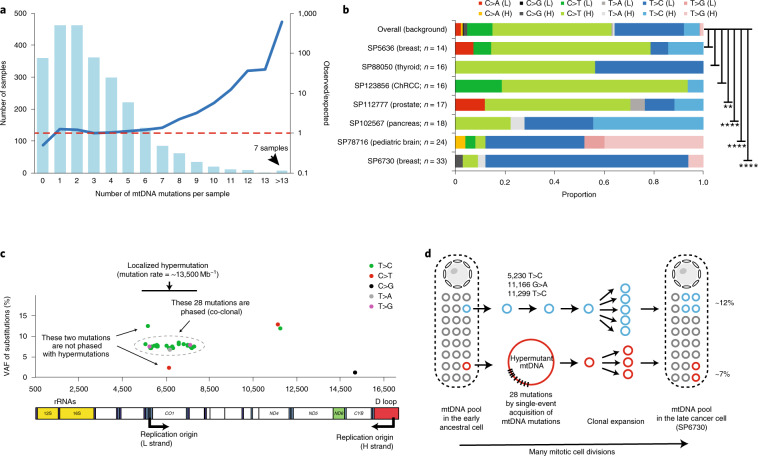
Characterization of hypermutated cancer mitochondrial genomes. **a**, Distribution of mtDNA mutations. The blue curve represents the calculated ratio between observed and expected numbers of samples for each bin (right-hand *y* axis). **b**, Mutational spectrum of the seven hypermutated mitochondrial genomes identified. The *P* values were generated by chi-squared test without multiple comparison adjustment (**P < 0.01; ****P < 0.0001). **c**, Distribution of the 33 somatic mutations in the breast cancer sample of mtDNA hypermutation (sample ID: SP6730). **d**, Proposed model of the mtDNA hypermutation process in SP6730. rRNAs, ribosomal RNAs.

Hypermutation processes have been well established for a small proportion of cancer nuclear genomes (for example, microsatellite instability)^26,27^, but have not been reported for mitochondrial genomes. Of the 2,536 cancer samples surveyed, seven cases showed extremely large numbers of mtDNA somatic substitutions (>13 mutations), which were larger than expected from the background distribution (Fig. 2a; around three somatic substitutions per sample on average, with a standard deviation of 2.6). The mutational spectra in these hypermutated samples were sometimes clearly distinguished from the background L-strand G>A and T>C substitution dominant signature (Fig. 2b), suggesting that the massive numbers of mutations are not the consequence of the gradual accumulation of ordinary mtDNA substitutions.

The most striking case was a breast cancer sample (sample ID: SP6730) harboring 33 mutations, 30 of which were localized in a 2-kb region (Fig. 2c), resulting in a local hypermutational rate (>75× higher than the background mutational rate). The mutations were neither of germline origin (~70% were novel) nor caused by sequencing errors, as confirmed by independent exome and RNA-seq analyses (Supplementary Fig. 9). Interestingly, most of the localized mutations (*n* = 28) were T>C substitutions on the L strand (Fig. 2b,c) and were co-clonal of each other, with highly similar VAFs (~7%) and direct physical phasing by Illumina sequencing reads (Supplementary Fig. 9). Collectively, these lines of evidence strongly suggest that the 28 localized mutations (19 missense, four silent and five tRNA mutations) were acquired by a ‘single-hit’ catastrophic mutational mechanism with strand-specific T>C substitutions as a dominant spectrum, reminiscent of the kataegis phenomenon in the nuclear genome^28^ (Fig. 2c) and/or complex somatic mutations reported in mtDNA^29^. The mutated mtDNA copy is then likely to shift to appreciable VAF (~7% frequency) by a series of replications throughout the cell lineages, despite the low probability of causation of a defective phenotype.

### Cancer type-specific selective pressures on mtDNA mutations

To investigate the functional consequences of mtDNA genes, we examined the dN/dS ratio (a common measure of selective pressure on missense mutations) with consideration of the unique mtDNA mutational signature^9^. We found that dN/dS was overall close to 1 for missense mutations at different VAFs across cancer types, suggesting that overall selection for mtDNA missense mutations is nearly neutral (Supplementary Fig. 10). However, it should not be interpreted that all missense mtDNA mutations are passengers.

**Fig. 3:**
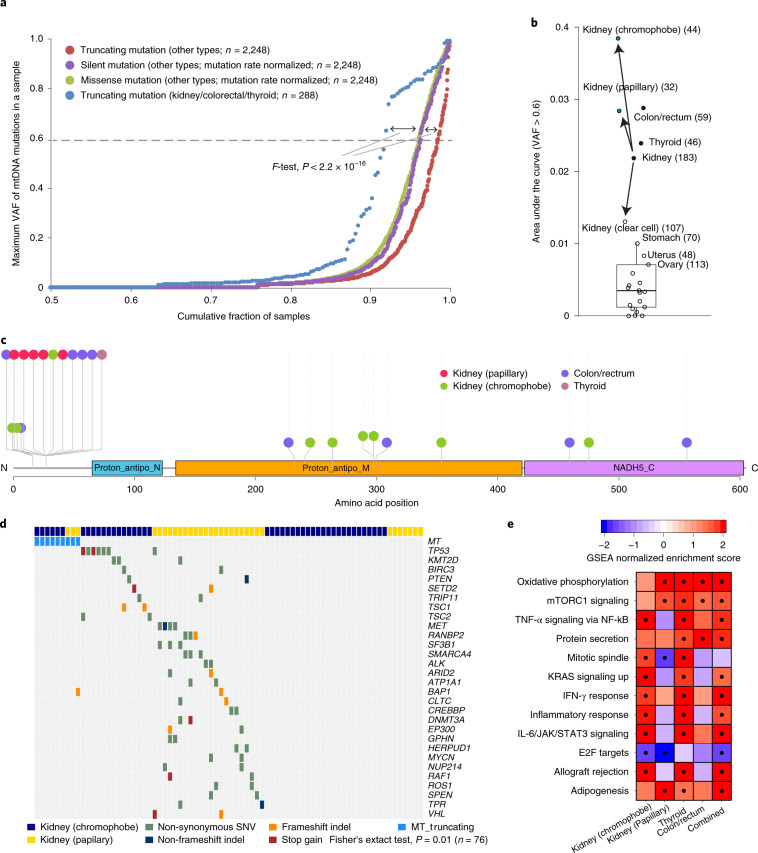
mtDNA truncating mutation patterns. **a**, Distinct VAF accumulation curves of truncating mutations between kidney/colorectal/thyroid cancers and other cancer types. For comparison, similar curves were generated for silent and missense mutations, which are overall functionally neutral, in other types of cancer after normalization of mutation numbers. Generally, fewer truncating mutations were observed at higher allele-frequency levels (red), except for kidney, colorectal and thyroid cancer types (blue). *n* = number of samples. **b**, Kidney chromophobe, kidney papillary, colorectal and thyroid cancers accumulated excessive high-allele-frequency truncating mutations (sample sizes in parentheses). Areas under the curve across cancer types for a VAF interval of 0.6–1.0 (from Supplementary Fig. 11a); were calculated and are shown. Their distribution is shown by a box plot. The boundaries of the box mark the first and third quartile, with the median in the center, and whiskers extending to 1.5× the interquartile range from the boundaries. **c**, Distribution patterns of truncating mutations in *ND5*. C, carboxy; N, amino; Proton_antipo_N, NADH-ubiquinone oxidoreductase (complex I), chain 5 N-terminus; Proton_antipo_M, proton-conducting membrane transporter; NADH5_C, NADH dehydrogenase subunit 5 C terminus. **d**, Heat map of mtDNA truncating mutations with recurrent somatic mutations in cancer genes in kidney chromophobe and kidney papillary cancers. MT_truncating standas for mitochondrial truncating mutations, which include frameshift mutations and stop-gain mutations. Statistical significance of mutual exclusivity between mutations was assessed by Fisher’s exact test. SNV, single-nucleotide variant. **e**, Heat map of the signaling pathways enriched by nuclear genes upregulated in cancer samples with truncating mutations. A dot indicates FDR < 0.05. IFN-γ, interferon-γ; IL-6, interleukin-6; JAK, Janus kinase; mTORC1, mammalian target of rapamycin complex 1; NF-κB, nuclear factor κB; STAT3, signal transducers and activators of transcription 3; TNF-α, tumor necrosis factor-α; KRAS, KRAS proto-oncogene, GTPase.

For truncating mutations on the 13 mtDNA genes, we found evidence of negative selection in most cancer types, suggesting the importance of intact mitochondrial function in cancer cells. For example, the VAFs of mtDNA truncating mutations were notably more suppressed than those of missense or silent mutations (Fig. 3a). Interestingly, kidney, colorectal and thyroid cancers showed the opposite trend, where mtDNA truncating mutations exhibited significantly higher VAFs than the background (*F*-test, *P* < 2.2 × 10^−16^; Fig. 3a). The enrichment of nearly homoplasmic (>60% VAF) truncating mutations was very striking in kidney cancers, especially in chromophobe and papillary types, suggesting that the inactivation of the normal mitochondrial function is an important step in tumorigenesis^30^ (Fig. 3b and Supplementary Fig. 11). The mtDNA truncating mutations were enriched in *ND5*. Compared with kidney chromophobe and colorectal cancers, kidney papillary cancers harbored *ND5* truncation mutations enriched in the amino-terminal region (Fisher’s exact test, *P* = 0.05; Fig. 3c). Integrating with the mutation data of nuclear genes, we found that the high VAF truncating mutations in the two kidney cancer types were mutually exclusive to the mutations of known cancer genes (Fisher’s exact test, *P* = 0.01; Fig. 3d). Moreover, samples with mtDNA truncating mutations showed upregulation of gene expression in cancer-related pathways, such as mammalian target of rapamycin signaling, tumor necrosis factor-α signaling, oxidative phosphorylation and protein secretion (false discovery rate (FDR) < 0.05; Fig. 3e). Collectively, these results strongly suggest functional oncogenic impacts of mitochondrial truncating mutations in the initiation and clonal evolution of the specific cancer types.

### Somatic transfer of mtDNA into the nuclear genome

**Fig. 4:**
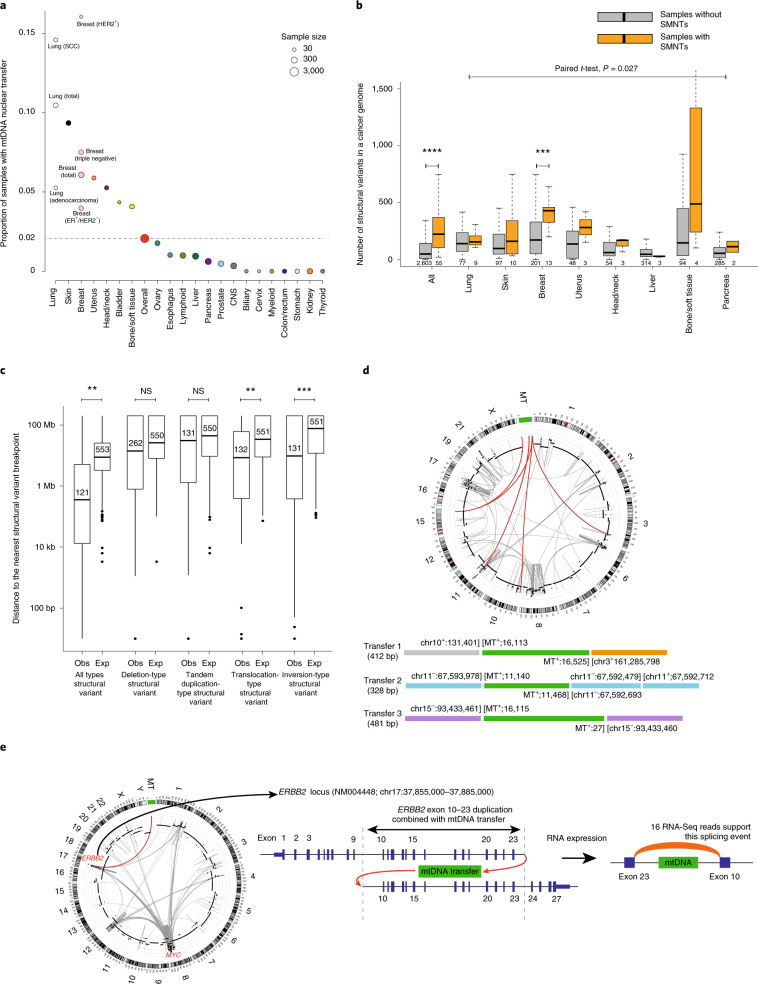
Somatic transfer of mtDNA into the cancer nuclear genome. **a**, Frequency of SMNTs in different cancer tissues. Circle size indicates the sample size of a given cancer type. ER, estrogen receptor. **b**, Numbers of structural variant breakpoints in samples with and without SMNTs. Sample sizes are labeled below the boxes. The *P* values (***P < 0.001; ****P < 0.0001) were generated by two-sided *t*-test without multiple comparison adjustment. **c**, Distances from SMNT breakpoints to the nearest structural variant breakpoints are shorter than random expectation for all and each type of structural variant. Sample sizes are labeled in the centers of the boxes. The *P* values (**P < 0.01; ***P < 0.001) were generated by two-sided *t*-test without multiple comparison adjustment. bp, base pair; exp, expected; Mb, megabase; NS, not significant; obs, observed. **d**, Circos plot of three independent SMNT events in a bladder cancer genome (sample ID: SP953), showing 23 human chromosomes in the outer layer, as well as copy numbers of nuclear cancer genomes (inner layer; black dots); chromosomal rearrangements (gray curves) and SMNTs (red curves). A summary of three SMNTs with genomic coordinates in numbers is depicted below the Circos plot with breakpoints. **e**, An SMNT event found in a HER2^+^ breast cancer genome (sample ID: SP10563), leading to a tandem duplication process of *ERBB2* exons 10–23 and their subsequent expression. The novel exon junction is supported by the RNA reads from the corresponding RNA-seq data. In all boxplots, the boundaries of the boxes mark the first and third quartiles, with the median in the center, and whiskers extending to 1.5× the interquartile range from the boundaries.

The migration of mtDNA into the nuclear genome has been assessed using different technologies^31–33^. Recently, somatic mtDNA nuclear transfers (SMNTs) have been more systematically studied in nucleotide resolution^11^, mostly in breast cancers. In this study, of the 2,658 cancer cases across 21 tissue types, we found 55 positive cases (2.1% overall positive rate) (Methods). The SMNT rate varied according to the cancer tissue type (Fisher’s exact test, *P* < 1 × 10^−5^; Fig. 4a). For example, lung, skin, breast and uterine cancers showed frequencies higher than 5%. In particular, human epidermal growth factor receptor 2-positive (HER2^+^) breast cancers and squamous cell lung cancers showed positive rates of 16.0% (four out of 25 cases) and 14.6% (seven out of 48 cases), respectively, which were significantly higher than the average (Fisher’s exact test, *P* < 0.003 and *P* < 0.001, respectively). In contrast, we did not find any positive cases from blood, kidney, esophagogastric, liver, prostate and colorectal cancers. The samples with SMNTs showed a much higher number of global and local structural variations in the nuclear genome than the control samples^16^ (*P* = 1 × 10^−4^; Fig. 4b and Supplementary Fig. 12). SMNT integration sites (breakpoints) were spatially closer to inversion and translocation breakpoints than expected (Fig. 4c). These results suggest that the integration of mtDNA segments into nuclear DNA is often mechanistically combined with some specific processes underlying structural variations in the nuclear genome.

Despite the overall low SMNT frequency (~2%), some cancer samples showed up to three independent SMNT events (Fig. 4d and Supplementary Fig. 13). Sometimes, somatically transferred mtDNA segments were extensively rearranged (Supplementary Fig. 13b), implying extreme genomic instability at the time of the SMNT events. We observed 42 SMNT events in 35 tumor cases that were integrated in the middle of genes (*n* = 42), mostly in introns (*n* = 37), with a few events in the protein-coding regions (*n* = 3) and in the untranslated regions (*n* = 2) (Supplementary Table 3). Among these, open reading frames of at least 23 genes (23/42 = 55%), including cancer genes such as *ERBB2*, *FOLH1* and *ULK2*, were predicted to be altered by these SMNTs and their combined structural variant events in the vicinity (Supplementary Fig. 14). Of particular interest, one SMNT was involved in transforming focal amplification of the *ERBB2* gene in a HER2^+^ breast cancer genome (Fig. 4e).

### Copy-number and structural variations of mtDNA

Although previous studies have examined mtDNA copy numbers in individual cancer types^34–36^ or from a collection of whole-exome sequencing data^12^, we performed a systematic and accurate analysis of mtDNA copy numbers per cell over the largest sample cohort with WGS data so far, with consideration to confounding factors such as the normal-cell contamination and genome ploidy of tumor cells (Supplementary Fig. 15 and Methods).

**Fig. 5:**
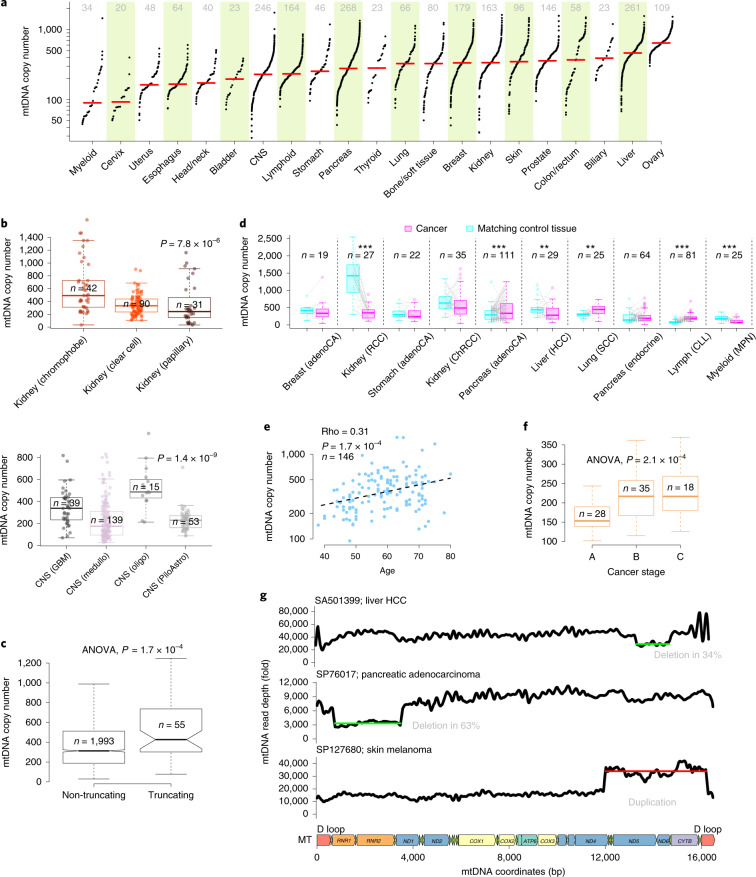
Pan-cancer view of mtDNA copy number. **a**, Distributions of mtDNA copy number by cancer tissue type. Sample numbers with mtDNA copy-number information available are labeled on the top, with the median mtDNA copy numbers marked as red bars. **b**, Distinct mtDNA copy-number distributions for cancer types derived from the kidney (top) and brain (bottom). *n* = number of samples with mtDNA copy number. *P* values were generated by ANOVA. **c**, mtDNA copy numbers with and without truncating mutations in mtDNA genes. *n* = number of samples with both mtDNA copy number and somatic mutation information available. *P* values were based on ANOVA, adjusting for cancer types. **d**, Paired copy-number comparison of tumor and matching normal tissue samples. *n* = number of matching normal tissue and cancer sample pairs. Raw *P* values were determined by two-sided Wilcoxon signed-rank test, then adjusted for FDR (**FDR < 0.01; ***FDR < 0.001). **e**, Correlation of mtDNA copy number with patient age in prostate cancer. *n* = number of samples with cancer mtDNA copy number and patient age information available. Correlations and *P* values are based on Spearman’s rank correlation. **f**, Correlation of mtDNA copy number with cancer stage in chronic lymphocytic leukemia. *n* = number of samples with mtDNA copy number and stage information. **g**, Focal copy gain and loss caused by structural variations in three cancer samples. Green lines represent focal loss, whereas red lines represent tandem duplication. In all boxplots, the boundaries of the boxes mark the first and third quartile, with the median in the center, and whiskers extending to 1.5× the interquartile range from the boundaries.

Based on the 2,157 cancer samples that passed the purity filter, we observed great variation in mtDNA copy numbers across and within cancer types: mtDNAs were most abundant in samples of ovarian cancer (median: 644 copies per cell) and least abundant in myeloid cancer (median: 90 copies per cell) (Fig. 5a). Different cancer subtypes originating from the same tissue sometimes showed distinct mtDNA copy-number distributions (Fig. 5b and Supplementary Fig. 16). For example, the mtDNA copy numbers for kidney chromophobe were significantly higher than those for kidney clear cell and kidney papillary (analysis of variance (ANOVA), *P* < 7.8 × 10^−6^; Fig. 5b). This may be interlinked with the general inadequacy of mitochondrial quality control and resultant increase in the steady-state mtDNA copy number, as seen in renal oncocytoma^37^. Indeed, we found that the mtDNA copy number was significantly higher in the samples with high-allele-frequency truncating mutations (ANOVA with consideration of confounders, *P* < 1.7 × 10^−4^; Fig. 5c), suggesting that the dosage effect of mtDNAs was selected to compensate for the deleterious effect of truncating mutations. For the cancer samples with WGS data from matched normal tissues (*n* = 507), we observed increased mtDNA copy numbers in cancer samples in patients with chronic lymphocytic leukemia, lung squamous cell carcinoma and pancreatic adenocarcinoma, but decreased copy numbers in cancer samples in patients with kidney clear cell carcinoma, hepatocellular carcinoma and myeloproliferative neoplasm (Fig. 5d). At face value, the distinct patterns in different cancer types may be due to cancer-specific oncogenic stimulation, metabolic activity and mitochondrial malfunctions. For example, a recent study^12^ suggested that significantly decreased mtDNA copy number in kidney clear cell cancer may be due to downregulation of peroxisome proliferator-activated receptor-γ coactivator 1α (a central regulator of mitochondrial biogenesis) by hyperactivated hypoxia-inducible factor 1α, which is most frequently mutated and activated in this disease^38^. However, since the available mtDNA copy numbers in normal tissues are average values from mixtures of many heterogeneous cell types with unknown relative contributions, a direct comparison between tumor and adjacent normal tissues should be interpreted cautiously.

To assess the potential biomedical significance of mtDNA copy numbers, we examined their correlations with key clinical variables. We found significant positive correlations between the mtDNA copy number and the patient’s age at diagnosis in prostate (Spearman’s rank, *R*_s_ = 0.31; *P* < 1.7 × 10^−4^; Fig. 5e), colorectal and skin cancers (Supplementary Fig. 17). In contrast, we observed negative correlations of normal blood mtDNA copy number with patient age in most cases (Supplementary Fig. 18). We further observed correlations between mtDNA copy number and tumor stage in multiple cancer types (Fig. 5f and Supplementary Fig. 19).

Using WGS data, we examined the focal copy gain and loss in the mitochondrial genomes that were known to be present in prostate cancers and aged tissues^39^. Of the 2,658 cancer samples, three (0.11%) showed notable structural variants in the mtDNA (Fig. 5g). For example, a pancreatic cancer case (sample ID: SP76017) harbored a ~3.4-kb-long mtDNA loss that truncated ribosomal RNA and *ND1* genes. The VAF of this mutant mtDNA was estimated at 63%. Similarly, a melanoma case (sample ID: SP127680) showed tandem duplication of an mtDNA segment of ~4 kb, with 100% VAF. Thus, our analysis identified structural variants in mtDNA genomes based on WGS.

### Co-expression network analysis of mitochondrial genes

**Fig. 6:**
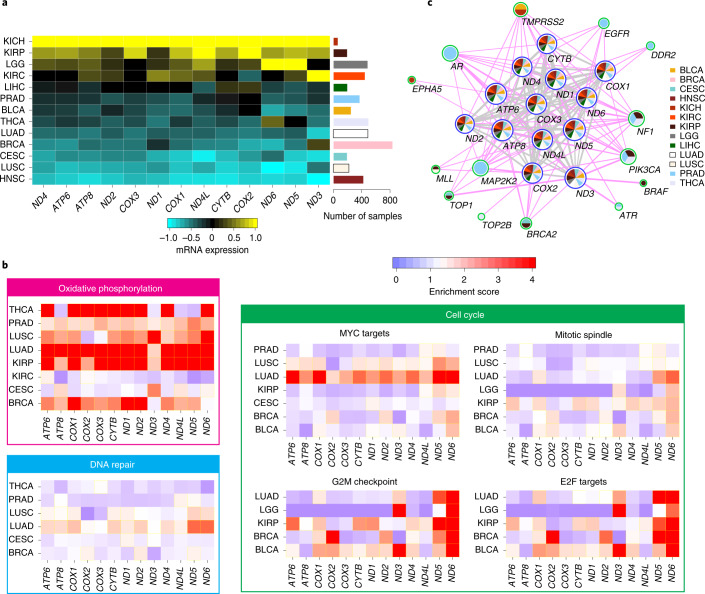
Co-expression patterns of mtDNA genes across different cancer types. **a**, Left: heat map of the expression levels of 13 mtDNA genes of 13 cancer types. Right: bar plot showing the sample sizes for each cancer type. **b**, Commonly enriched pathways identified by co-expression with mtDNA genes in different cancer types. Borders of cells with FDR < 0.05 are highlighted in yellow. **c**, mtDNA gene-centric pan-cancer co-expression network. The pie chart colors at each node indicate occurrence of the node in cancer types of the corresponding colors. Green borders, nuclear genes; blue borders, mitochondrial genes. Node size is proportional to the number of direct neighbors (connectivity) of the node. Thickness of the edge is proportional to the frequency of this edge being observed across all cancer types. Edges are colored according to the connection type (gray: mtDNA gene–mtDNA gene connection; magenta: mtDNA gene–nuclear gene connection). BLCA, bladder urothelial carcinoma; BRCA, breast invasive carcinoma; CESC, cervical squamous cell carcinoma and endocervical adenocarcinoma; HNSC, head and neck squamous cell carcinoma; KICH, kidney chromophobe; KIRC, kidney renal clear cell carcinoma; KIRP, kidney renal papillary cell carcinoma; LGG, brain lower-grade glioma; LIHC, liver hepatocellular carcinoma; LUAD, lung adenocarcinoma; LUSC, lung squamous cell carcinoma; PRAD, prostate adenocarcinoma; THCA, thyroid carcinoma.

To understand the functional impact of 13 mtDNA genes in cancers, we quantified the gene expression levels using RNA-seq data profiled from 4,689 TCGA tumor samples of 13 cancer types (Supplementary Table 4). The correlation between the gene expression levels and the mtDNA copy number varied by cancer type (Supplementary Fig. 20). Among the cancer types, the mtDNA genes were highly expressed in the three types of kidney cancer (chromophobe, papillary and clear cell) but weakly expressed in the three types of squamous cell carcinoma (cervical, lung and head and neck) (Fig. 6a). This observation was partially due to the relative abundance of mtDNA copy number across cancer types and is consistent with a study of normal tissues^40^.

To gain more insight into the functions of mtDNA genes and their related nuclear genes and pathways, for each cancer type, we used the weighted gene co-expression network analysis (WGCNA) package^41^ to build a weighted gene co-expression network that consisted of both nuclear genes and mitochondrial genes (Methods). We then performed gene set enrichment analysis (GSEA)^42^ based on the rank of all nuclear genes by measuring their edge strength to a mitochondrial gene in the co-expression network. We found oxidative phosphorylation to be the top-ranked enriched pathway, and to be enriched in eight out of the 13 cancer types examined (FDR < 0.05), highlighting the essential role of mitochondrial genes in energy generation (Fig. 6b). Pathways related to the cell cycle (MYC targets, mitotic spindle, G2/M checkpoint and E2F targets) and DNA repair were also enriched in multiple cancer types (Fig. 6b), consistent with the established notion that mtDNA plays an important role in these pathways^37,43^.

We also examined the mtDNA-centric co-expression networks (Fig. 6c and Methods). Across cancer types, the mtDNA genes were almost always strongly interconnected, which is expected since they are transcribed as long polycistronic precursor transcripts^44^. Interestingly, several clinically actionable genes were among the neighboring genes that showed strong co-expression patterns with mtDNA genes (Fig. 6c and Supplementary Fig. 21). For example, *AR*, *EGFR*, *DDR2* and *MAP2K*2 were connected with mtDNA genes in prostate cancer, and *TMPRSS2*, *NF1*, *PIK3CA*, *BRCA1* and *TOP1* were the top neighbors of mtDNA genes in multiple cancer types. This correlation-based analysis does not necessarily suggest causality, and further efforts are needed to investigate these relationships. Elucidating the underlying mechanisms may lay a foundation for developing mtDNA-related cancer therapy.

### An open-access Cancer Mitochondrial Atlas data portal

To facilitate mitochondria-related biological discoveries and clinical applications, we developed an open-access, user-friendly data portal, The Cancer Mitochondrial Atlas (TCMA), for fluent exploration of the various types of molecular data characterized in this study (Supplementary Fig. 22). The data portal can be accessed at http://bioinformatics.mdanderson.org/main/TCMA:Overview. There are four modules in TCMA: somatic mutations, nuclear transfer, copy number and gene expression. The first three modules are based on the ICGC WGS data and provide detailed annotations for the corresponding features of each cancer sample. The last module is based on TCGA RNA-seq data and provides an interactive interface through which users can visualize the co-expression network with convenient navigation and zoom features. Not only can users browse and query the molecular data by cancer type, they can also download all of the data for their own analysis.

## Discussion

This work characterizes the cancer mitochondrial genome in a comprehensive manner, including somatic mutations, nuclear transfer, copy number, structural variants and mtDNA gene expression. Because of the ultra-high coverage of mtDNA from the WGS data and the large number of patient samples surveyed, our study provides a definitive landscape of mtDNA somatic mutations and identifies several unique features. First, we report hypermutated mitochondrial cases, highlighting the dynamic mutational processes in this tiny genome. Second, our systemic analysis of mitochondrial genomes has firmly shown that several cancer types are enriched for high-allele-frequency truncating mutations, including previously reported kidney chromophobe^30,45^ as well as newly identified kidney papillary, and thyroid and colorectal cancers. Interestingly, the thyroid and kidney are the most frequent sites of oncocytomas, which are rare, benign tumors characterized by frequent nuclear chromosomal aneuploidy as well as vast accumulation of defective mitochondria^45,46^, further assuring the functional association between mitochondrial inactivation and the pathogenesis of these cancer types. Third, in contrast with the diversified mutational signatures observed in the nuclear genomes of different cancers^20^, mtDNAs show very similar mutational signatures regardless of cancer tissue origins: predominantly G>A and T>C substitutions on the L strand. This monotonous pattern may partially stem from different mutational generators and DNA repair processes between the nucleus and mitochondria^9,47,48^. Due to their large numbers of copies per cell, mitochondria may simply remove mtDNA damaged from external mutagens (for example, ultraviolet radiation, tobacco smoking and reactive oxygen species) through autophagy and other mitochondrial dynamic mechanisms^49^, rather than employing a complex array of repair proteins as in the nucleus.

One unique aspect of our study is the integrative analysis of mitochondrial molecular alterations with those in the nuclear genome that are characterized by the PCAWG Consortium. We found that: (1) high-allele-frequency truncating mtDNA mutations are mutually exclusive to mutated cancer genes in kidney cancer; (2) mtDNA nuclear transfers are associated with increased numbers of structural variants in the nuclear genome; and (3) mtDNA co-expressed nuclear genes are enriched in several processes critical for tumor development. These results indicate that the mitochondrial genome is an essential component in understanding the complex molecular patterns observed in cancer genomes and helping to pinpoint potential cancer driver events. Our results, such as the nuclear transfer of mtDNA into a therapeutic target gene, correlations of mtDNA copy numbers with clinical variables, and the co-expression of mtDNA and clinically actionable genes, underscore the clinical importance of mitochondria.

Taken together, this study has untangled and characterized the full spectrum of molecular alterations of mitochondria in human cancers. Our analyses have provided essentially complete catalogs of somatic mtDNA alterations in cancers, including substitutions, indels, copy-number alterations and structural variants. Furthermore, we have developed a user-friendly web resource to enable the broader biomedical community to capitalize on our results. These efforts lay a foundation for translating mitochondrial biology into clinical investigations.

## Methods

### Data generation and collection

We extracted BAM files of mtDNA sequencing reads from the whole-genome alignment files of 2,658 cancer samples and their matched normal tissue samples generated by the PCAWG Consortium. BWA was used to align the reads to the human reference genome (hs37d5). From the CGHub, we obtained TCGA RNA-seq BAM files of 13 cancer types, all of which employed paired-end sequencing strategies. We used Cufflinks to quantify the messenger RNA expression levels (in fragments per kilobase per million mapped fragments) of the 13 mitochondrial protein-coding genes. We obtained the nuclear somatic mutations and annotated driver mutations of corresponding samples as described^17^.

### Somatic mutation calling

The nuclear genome mutations were called using the Sanger pipeline, provided by the PCAWG. The mitochondrial variants were initially called using VarScan2 (ref. ^50^) and the same parameter setting as previously reported^9^: --strand-filter 1 (mismatches should be reported by both forward and reverse reads), --min-var-freq 0.01 (minimum VAF 1%), --min-avg-qual 20 (minimum base quality 20), --min-coverage × and --min-reads2 ×). We applied a series of downstream bioinformatic filters to further remove false positives as follows (Supplementary Fig. 2a).

First, we filtered germline polymorphisms and false positive calls (for example, frequent mapping errors due to known mtDNA homopolymers, candidates with substantial mapping strand bias and candidates with substantial mutant alleles in the matched normal sample). For analytic simplicity, we removed multi-allelic mtDNA mutations and back mutations from the non-reference to the reference allele. After this filtration step, we obtained 10,083 somatic substitution candidates.

Second, we examined DNA cross-contamination because even minor DNA cross-contamination (that is, contamination level < 3%) would generate many low-VAF false positive calls that are in fact germline polymorphisms from the contaminating sample. We tested whether mtDNA somatic mutations detected from a cancer sample show greater overlap with known mtDNA polymorphisms than expected from the overall average rate (73.5%; 3,922/5,337 substitutions) using the binomial test with a cutoff *P* < 0.01. From this step, we removed 96 samples with evidence of DNA cross-contamination (harboring 935 known mutations out of 1,131 known mutation candidates).

Third, we examined the overall mtDNA substitution signatures in the 96 possible mutation classes. We removed four samples with extremely high proportions of C>G substitutions with strong sequence context bias (at CpCpN>CpApN; most frequently at CpCpG>CpApG; Supplementary Fig. 2b). This spectrum is known to arise from artificial guanine oxidation during sequencing library preparation steps^19^ with low VAF (1–2%). We explicitly removed these samples from further analyses.

Then, we examined the possibility of false positive calls due to mismapping of reads from inherited nuclear mtDNA-like sequences (known as numts) not represented in the human reference genome^18^, especially when the specific numts regions were amplified in the cancer nuclear genome. These mutation candidates showed some specific features: (1) they appeared as highly recurrent mtDNA somatic mutations among multiple samples; (2) VAFs in mitochondria were only slightly higher than our 1% cutoff criteria; and (3) the matched normal samples also had small but substantial numbers of mutation allele counts. To remove these false positive calls, we applied two statistical tests of: (1) whether the VAF of a mutation candidate in the matched normal sequences was within the normal range (<0.0024; the cutoff is determined by the median VAF of all mutation candidates +2× the interquartile range); and (2) whether:

$$\frac{{N_{{\rm{mut}}_{{\rm{nor}}}}/{\rm{RD}}_{{\rm{nor}}}}}{{(N_{{\rm{mut}}_{{\rm{nor}}}}/{\rm{RD}}_{{\rm{nor}}} + N_{{\rm{mut}}_{{\rm{tum}}}}/{\rm{RD}}_{{\rm{tum}}})}}$$

was within the normal range (<0.0357; the cutoff is determined by the median VAF of all mutation candidates +2× the interquartile range), where *N*_mut_ is the mutation allele count, RD is the average read depth for the nuclear genome, and nor and tum are normal and matched tumor tissues, respectively. When a mutation appeared to be an outlier according to both criteria, we removed the candidate from our downstream analyses.

In our previous study^9^, we could not detect mutations under a 3% VAF cutoff because mtDNA was sequenced with a read depth of ~100× from the majority of samples surveyed. Taking advantage of the ultra-high depth (>8,000×) in this study, we used a 1% VAF cutoff to obtain better sensitivity. We found 2,133 more substitutions when the VAF was between 1 and 3%. Because of the ultra-high depth, even 1% VAF mutations were considered to be specific, and were supported by a high number (*n* = ~80) of mutation alleles. We confirmed the high specificity of these mutations using the unique mtDNA mutational signatures robustly observed even from these low-VAF mutations: (1) the mutational spectrum is generally consistent with those from higher heteroplasmic levels of mutations (that is, VAFs from 3–10% and 10–100%); (2) we observed the absolute dominance of C>T and T>C substitutions in the expected trinucleotide contexts (NpCpG for C>T and NpTpC for T>C substitutions); and (3) we also observed extreme replication strand bias (Supplementary Fig. 3). These features would not be observed if contaminations resulted in many false positive calls. To assess the factors affecting the mutation frequency of the 13 coding genes, we performed the sample-level analysis using log-linear modeling: we assigned the binary mutation indicator (1: with mutation; 0: without mutation) to each sample for each gene and then fit this binary response variable to a logistic regression model, including cancer type, gene identity and their interaction as explanatory variables, which were later summarized using ANOVA. In addition, within each cancer type, we used Spearman’s rank correlation to assess the association between the numbers of nuclear and mtDNA somatic mutations, as well as their individual association with patient age.

### Truncating mutation analysis

Taking into account the mtDNA-specific mutational signature, we examined the dN/dS ratio for mtDNA missense substitutions as reported previously^9^. We defined truncating mutations as those that lead to truncated protein products (that is, nonsense mutations and frameshift indels), and accordingly categorized the samples into the truncating group (bearing at least one truncating mutation with VAF ≥ 60%). The ND5 protein domain information was obtained from Pfam (http://pfam.xfam.org/protein/P03915). The cancer gene census list was obtained from http://cancer.sanger.ac.uk/cosmic/download. Cancer census genes with recurrent somatic mutations in kidney chromophobe and kidney papillary cancers were selected for analysis of mutual exclusivity and heat-map representation. One sample with a nuclear DNA hypermutator phenotype was excluded from this analysis. To examine the functional consequences of mtDNA truncating mutations, we performed GSEA based on the ranks of differentially expressed genes between samples with and samples without mtDNA truncating mutations for kidney chromophobe, kidney papillary, colorectal and thyroid cancers and their combination, and identified significantly enriched pathways at FDR = 0.05.

### SMNT analysis

We examined the WGS data from the cancer and matched control tissue samples using a pipeline for the identification of mtDNA translocation to the nuclear genome, as reported previously^11^. The specificity was shown to be 100% in the previous study^11^. Briefly, we extracted and clustered discordant reads from cancer genomes, where one end aligned to nuclear DNA and the other aligned to mtDNA. Then, to determine the nucleotide resolution breakpoints, we searched for split reads near putative breakpoint junctions (1,000 base pairs upstream and downstream), where a fraction of a single read aligned to genomic DNA near the junctions and the rest aligned to mtDNA. All filtering criteria were the same as previously reported, except that we did not use BLAT^51^ for split-read detection because the BWA-MEM alignment tool used to map all pan-cancer samples fundamentally enables split-read mapping. We removed candidate mitochondrion–nuclear DNA junctions that overlapped with clusters from matched and unmatched normal samples and/or known human SMNTs—a combined set from the human reference genome (hg19; *n* = 123) and a published study^52^ (*n* = 766)—because the source of the mtDNA sequence fused to the nuclear genome might be SMNTs rather than real mitochondria in the cytoplasm of cells. We obtained the PCAWG Structural Variation Working Group^16^ and compared the samples with and without SMNTs by *t*-test. To study the relationship of SMNTs and structural variant breakpoints, we randomly chose the same number of structural variant breakpoints from each sample 100 times to estimate the random expectation.

### MtDNA copy-number analysis

To better estimate the mtDNA copy number for cancer samples, we employed the following formula, which incorporates both tumor purity and ploidy information:

$${\rm{CN}}_{{\rm{tumor}}} = \frac{{{\rm{coverage}}\_{\rm{depth}}_{{\rm{mtDNA}}}}}{{{\rm{coverage}}\_{\rm{depth}}_{{\rm{gDNA}}}}}(f \times{\rm{ploidy}}_{{\rm{cancer}}}+(1 - f) \times 2)$$

where *f* is the tumor purity (ranging from 0 to 1, where 1 stands for pure cancer cells and 0 stands for pure normal cells), CN is the mtDNA copy number, coverage_depth_mtDNA_ and coverage_depth_gDNA_ are the mean coverage depths for mtDNA and the nuclear genome in individual WGS BAM files, respectively, and ploidy_cancer_ is the number of sets of chromosomes in tumor cells, while ploidy in the normal cells is 2. Both *f* and ploidy_cancer_ were obtained using allele-specific copy-number analysis of tumors estimation^53^, provided by the PCAWG Consortium. Donors with multiple samples were preselected so that each donor came with one representative primary cancer sample. We excluded cancer samples with low purity (<0.4, estimated by allele-specific copy-number analysis of tumors) for further downstream analyses. We used ANOVA (if there were more than two cancer types) or *t*-test to compare the mtDNA copy number of cancer types derived from the same tissue. Since many of the normal samples were from blood, we focused on the cancer types with at least ten samples from the normal tissue adjacent to the tumor in order to compare the mtDNA copy number of the paired cancer and normal samples. We used the Wilcoxon signed-rank test to compare the mtDNA copy number for each selected cancer type and further adjusted the raw *P* values based on the FDR. To assess the correlation of mtDNA copy number with truncating mutations, we employed ANOVA (with the cancer type included in the model, to account for its potential effect). We assessed the correlations of the mtDNA copy number with the patient’s age, overall survival time and cancer stage using Spearman’s rank correlation, Cox model/log-rank test and ANOVA, respectively. We log_2_-transformed the mtDNA copy-number values when using ANOVA and the *t*-test, to conform to the normality assumption.

### mtDNA structural variation analysis

To investigate large deletions or duplications in the mtDNA genome, we sought the read-depth change of tumor mtDNA sequences using normal mtDNA sequences as a reference. To this end, we calculated the normalized depth of mtDNA loci in 100-base pair-sized bins from all of the normal samples. Then, we calculated the deviation of mtDNA read depth in each tumor sample. When ten bins were consecutively increased or decreased in the relative depth sufficiently (*z* score > 3), we considered the region as a structural variation candidate. From all of the candidates, we sought discordant paired-end reads, or breakpoint-spanning reads, which strongly support structural variations^11^.

### Co-expression analysis

For each cancer type, we used the WGCNA package^41^ to build a weighted gene co-expression network that contains ~20,000 nodes (including both nuclear genes and mitochondrial genes). The key parameter, *β*, for a weighted network construction was optimized to maintain both the scale-free topology and sufficient node connectivity, as recommended in the manual. In such a network, any two genes were connected and the edge weight was determined by the topology overlap measure provided in WGCNA. This measure considered not only the expression correlation between two partner genes, but also how many ‘friends’ the two genes shared. The weights ranged from 0 to 1, which reflected the strength of the connection between the two genes. To identify mitochondria-related pathways, we performed GSEA^42^ on the basis of the full set of nuclear protein-coding genes, ranked on the basis of the weights of the edge connecting the mitochondrial genes, and detected significant pathways at FDR = 0.05. To construct the mitochondria-centric network, we focused on the top 500 neighboring genes that showed the strongest connections with the mitochondrial genes, with a minimum weight of 0.05. Among these neighboring genes, we detected the clinically actionable genes (defined as FDA-approved therapeutic targets and their relevant predictive markers^54^) in at least one of the cancer types we surveyed. We examined the correlations of mtDNA gene expression levels with mtDNA copy numbers using Spearman’s rank correlations.

### TCMA data portal construction

We stored the precalculated mtDNA molecular data (including mtDNA mutation, nuclear transfer, copy number and expression) in a database of CouchDB. The Web interface was implemented by JavaScript, tables were visualized by DataTables, and the co-expression network visualization was implemented by Cytoscape Web.

### Reporting Summary

Further information on research design is available in the Nature Research Reporting Summary linked to this article.

## Online content

Any methods, additional references, Nature Research reporting summaries, source data, extended data, supplementary information, acknowledgements, peer review information; details of author contributions and competing interests; and statements of data and code availability are available at 10.1038/s41588-019-0557-x.

## Supplementary information

Supplementary Information Supplementary Figs. 1–22 and Tables 1–4

Reporting Summary

## Data Availability

Somatic and germline variant calls, mutational signatures, subclonal reconstructions, transcript abundance, splice calls and other core data generated by the ICGC/TCGA PCAWG Consortium are described here^14^ and are available for download at https://dcc.icgc.org/releases/PCAWG. Additional information on accessing the data, including raw read files, can be found at https://docs.icgc.org/pcawg/data/. In accordance with the data access policies of the ICGC and TCGA projects, most molecular, clinical and specimen data are in an open tier that does not require access approval. To access information with the potential to identify individuals, such as germline alleles and underlying sequencing data, researchers will need to apply to the TCGA Data Access Committee via the Database of Genotypes and Phenotypes (https://dbgap.ncbi.nlm.nih.gov/aa/wga.cgi?page=login) for access to the TCGA portion of the dataset, and to the ICGC Data Access Compliance Office (http://icgc.org/daco) for the ICGC portion. In addition, to access somatic single-nucleotide variants derived from TCGA donors, researchers will also need to obtain Database of Genotypes and Phenotypes authorization. Derived datasets described specifically in this study are available from the TCMA data portal (http://bioinformatics.mdanderson.org/main/TCMA:Overview).

## Code availability

The core computational pipelines used by the PCAWG Consortium for alignment, quality control and variant calling are available to the public at https://dockstore.org/search?search=pcawg under GNU General Public License version 3.0, which allows for reuse and distribution.

## References

[1] Schon EA, DiMauro S, Hirano M (2012). Human mitochondrial DNA: roles of inherited and somatic mutations. Nat. Rev. Genet..

[2] Smeitink J, van den Heuvel L, DiMauro S (2001). The genetics and pathology of oxidative phosphorylation. Nat. Rev. Genet..

[3] Anderson S (1981). Sequence and organization of the human mitochondrial genome. Nature.

[4] Brandon M, Baldi P, Wallace DC (2006). Mitochondrial mutations in cancer. Oncogene.

[5] Zong WX, Rabinowitz JD, White E (2016). Mitochondria and cancer. Mol. Cell.

[6] Hanahan D, Weinberg RA (2011). Hallmarks of cancer: the next generation. Cell.

[7] Hengartner MO (2000). The biochemistry of apoptosis. Nature.

[8] Larman TC (2012). Spectrum of somatic mitochondrial mutations in five cancers. Proc. Natl Acad. Sci. USA.

[9] Ju YS (2014). Origins and functional consequences of somatic mitochondrial DNA mutations in human cancer.. eLife.

[10] Stewart JB (2015). Simultaneous DNA and RNA mapping of somatic mitochondrial mutations across diverse human cancers. PLoS Genet..

[11] Ju YS (2015). Frequent somatic transfer of mitochondrial DNA into the nuclear genome of human cancer cells. Genome Res..

[12] Reznik E (2016). Mitochondrial DNA copy number variation across human cancers. eLife.

[13] Hopkins JF (2017). Mitochondrial mutations drive prostate cancer aggression. Nat. Commun..

[14] The ICGC/TCGA Pan-Cancer Analysis of Whole Genomes Consortium. Pan-cancer analysis of whole genomes. *Nature*10.1038/s41586-020-1969-6 (2020). 10.1038/s41586-020-1969-6 PMC7025898 32025007

[15] The Cancer Genome Atlas Research Network et al. The Cancer Genome Atlas pan-cancer analysis project. *Nat. Genet.* **45**, 1113–1120 (2013). 10.1038/ng.2764 PMC3919969 24071849

[16] Li, Y. et al. Patterns of somatic structural variation in human cancer genomes. *Nature*10.1038/s41586-019-1913-9 (2020). 10.1038/s41586-019-1913-9 PMC7025897 32025012

[17] Rheinbay, E. et al. Analyses of non-coding somatic drivers in 2,693 cancer whole genomes. *Nature*10.1038/s41586-020-1965-x (2020). 10.1038/s41586-020-1965-x PMC7054214 32025015

[18] Dayama G, Emery SB, Kidd JM, Mills RE (2014). The genomic landscape of polymorphic human nuclear mitochondrial insertions. Nucleic Acids Res..

[19] Costello M (2013). Discovery and characterization of artifactual mutations in deep coverage targeted capture sequencing data due to oxidative DNA damage during sample preparation. Nucleic Acids Res..

[20] Lawrence MS (2013). Mutational heterogeneity in cancer and the search for new cancer-associated genes. Nature.

[21] Kennedy SR, Salk JJ, Schmitt MW, Loeb LA (2013). Ultra-sensitive sequencing reveals an age-related increase in somatic mitochondrial mutations that are inconsistent with oxidative damage. PLoS Genet..

[22] Tanaka M, Ozawa T (1994). Strand asymmetry in human mitochondrial DNA mutations. Genomics.

[23] Zheng W, Khrapko K, Coller HA, Thilly WG, Copeland WC (2006). Origins of human mitochondrial point mutations as DNA polymerase γ-mediated errors. Mutat. Res..

[24] Alexandrov LB (2015). Clock-like mutational processes in human somatic cells. Nat. Genet..

[25] Coller HA (2001). High frequency of homoplasmic mitochondrial DNA mutations in human tumors can be explained without selection. Nat. Genet..

[26] The Cancer Genome Atlas Network. (2012). Comprehensive molecular characterization of human colon and rectal cancer. Nature.

[27] The Cancer Genome Atlas Research Network et al. Integrated genomic characterization of endometrial carcinoma. *Nature* **497**, 67–73 (2013). 10.1038/nature12113 PMC3704730 23636398

[28] Nik-Zainal S (2012). Mutational processes molding the genomes of 21 breast cancers. Cell.

[29] Pak JW, Vang F, Johnson C, McKenzie D, Aiken JM (2005). MtDNA point mutations are associated with deletion mutations in aged rat. Exp. Gerontol..

[30] Davis CF (2014). The somatic genomic landscape of chromophobe renal cell carcinoma. Cancer Cell.

[31] Caro P (2010). Mitochondrial DNA sequences are present inside nuclear DNA in rat tissues and increase with age. Mitochondrion.

[32] Chen D, Xue W, Xiang J (2008). The intra-nucleus integration of mitochondrial DNA (mtDNA) in cervical mucosa cells and its relation with c-myc expression. J. Exp. Clin. Cancer Res..

[33] Srinivasainagendra V (2017). Migration of mitochondrial DNA in the nuclear genome of colorectal adenocarcinoma. Genome Med..

[34] Cui H (2013). Association of decreased mitochondrial DNA content with the progression of colorectal cancer. BMC Cancer.

[35] Dickinson A (2013). The regulation of mitochondrial DNA copy number in glioblastoma cells. Cell Death Differ..

[36] Van Osch FH (2015). Mitochondrial DNA copy number in colorectal cancer: between tissue comparisons, clinicopathological characteristics and survival. Carcinogenesis.

[37] McBride HM, Neuspiel M, Wasiak S (2006). Mitochondria: more than just a powerhouse. Curr. Biol..

[38] Vyas S, Zaganjor E, Haigis MC (2016). Mitochondria and cancer. Cell.

[39] Lott MT (2013). mtDNA variation and analysis using mitomap and mitomaster. Curr. Protoc. Bioinformatics.

[40] Mercer TR (2011). The human mitochondrial transcriptome. Cell.

[41] Langfelder P, Horvath S (2008). WGCNA: an R package for weighted correlation network analysis. BMC Bioinformatics.

[42] Subramanian A (2005). Gene set enrichment analysis: a knowledge-based approach for interpreting genome-wide expression profiles. Proc. Natl Acad. Sci. USA.

[43] Koczor CA (2009). Mitochondrial DNA damage initiates a cell cycle arrest by a *Chk2*-associated mechanism in mammalian cells. J. Biol. Chem..

[44] Ojala D, Montoya J, Attardi G (1981). tRNA punctuation model of RNA processing in human mitochondria. Nature.

[45] Joshi S (2015). The genomic landscape of renal oncocytoma identifies a metabolic barrier to tumorigenesis. Cell Rep..

[46] Gasparre G, Romeo G, Rugolo M, Porcelli AM (2011). Learning from oncocytic tumors: why choose inefficient mitochondria?. Biochim. Biophys. Acta.

[47] Clayton DA, Doda JN, Friedberg EC (1974). The absence of a pyrimidine dimer repair mechanism in mammalian mitochondria. Proc. Natl Acad. Sci. USA.

[48] Haradhvala NJ (2016). Mutational strand asymmetries in cancer genomes reveal mechanisms of DNA damage and repair. Cell.

[49] Bess AS, Crocker TL, Ryde IT, Meyer JN (2012). Mitochondrial dynamics and autophagy aid in removal of persistent mitochondrial DNA damage in *Caenorhabditis elegans*. Nucleic Acids Res..

[50] Koboldt DC (2012). VarScan 2: somatic mutation and copy number alteration discovery in cancer by exome sequencing. Genome Res..

[51] Kent WJ (2002). BLAT—the BLAST-like alignment tool. Genome Res..

[52] Simone D, Calabrese FM, Lang M, Gasparre G, Attimonelli M (2011). The reference human nuclear mitochondrial sequences compilation validated and implemented on the UCSC genome browser. BMC Genomics.

[53] Van Loo P (2010). Allele-specific copy number analysis of tumors. Proc. Natl Acad. Sci. USA.

[54] Van Allen EM (2014). Whole-exome sequencing and clinical interpretation of formalin-fixed, paraffin-embedded tumor samples to guide precision cancer medicine. Nat. Med..

