# Supplementary material for: Comprehensive molecular characterization of mitochondrial genomes in human cancers
Source: Nat Genet. 2020 Feb 5;52(3):342–52. doi: 10.1038/s41588-019-0557-x (PMC7058535; doi:10.1038/s41588-019-0557-x)
Supplement: Supplementary file 1 — Supplementary Figs. 1–22 and Tables 1–4 [file 41588_2019_557_MOESM1_ESM.pdf]

nature portfolio

https://doi.org/10.1038/s41588-019-0557-x

Supplementary information

Comprehensive molecular characterization of mitochondrial genomes in human cancers

In the format provided by the authors and unedited

Supplementary Information

Comprehensive Molecular Characterization of the Mitochondrial Genomes in Human Cancers

Table of contents:

Supplementary Figures
Supplementary Figure 1. Comparison of mtDNA coverage depth for samples with both WGS and WGS data ….. **p.3**
Supplementary Figure 2. Filters to obtain sensitive and specific mtDNA somatic mutations ….. **p.4**
Supplementary Figure 3. Consistent mtDNA mutation spectrum for substitutions at VAF between 1%-3%………………………………………..… **p.5**
Supplementary Figure 4. Comparison of mtDNA somatic mutations at DNA and mRNA level... **p.6**
Supplementary Figure 5. An overview of the mutation frequency of the 13 mtDNA coding genes across cancer tissue types ………………………….… **p.7**
Supplementary Figure 6. Replicative strand-specific mutational spectrum of mtDNA substitutions………………………………………………………. **p.8**
Supplementary Figure 7. The proportion of C→T substitutions at dipyrimidine contexts (where UV light frequently generates mutations)……………………...… **p.9**
Supplementary Figure 8. The correlation between the number of mtDNA somatic SNVs and patient age….. **p.10**
Supplementary Figure 9. Hypermutated sample validated by WXS…………….. **p.11**
Supplementary Figure 10. dN/dS ratio from mtDNA somatic missense substitutions………... **p.12**
Supplementary Figure 11. The proportion of samples with truncating mutations by cancer types………………………………………………………………….. **p.13**
Supplementary Figure 12. The numbers of SV breakpoints in samples with and without somatic mtDNA nuclear transfers (SMNT) by SV types…………………. **p.14**
Supplementary Figure 13. Somatic nuclear transfer of mtDNA ……….......... **p.15**
Supplementary Figure 14. The functional consequence of the SMNT events....... **p.16**
Supplementary Figure 15. The comparison of mtDNA copy number calculated from simple estimation (using only coverage depth information) vs. our method (incorporating coverage depth, tumor purity and ploidy)…………………………. **p.17**
Supplementary Figure 16. The copy number distributions of mtDNA in multiple cancers derived from the same tissue type.....…………………………………... **p.18**
Supplementary Figure 17. The correlation between mtDNA copy number and patient age……………………………………………………………………….. **p.19**
Supplementary Figure 18. The correlation between normal blood mtDNA copy number and patient age……………………………………………………….. **p.20**
Supplementary Figure 19. The correlation between mtDNA copy number and patient cancer stage ……………………...…..……………………………… **p.21**
Supplementary Figure 20. The correlation between mtDNA copy number and gene expression……………………………………………………………….. **p.22**
Supplementary Figure 21. The gene co-expression network seeded on mtDNA genes………………………………………………………………………….. **p.23**
Supplementary Figure 22. An overview of the TCMA data portal……………... **p.24**

Supplementary Tables

Supplementary Table 1. An overview of the mtDNA whole-genome sequencing (WGS) data.. **p.25**

Supplementary Table 2. The high-allele-frequency mtDNA mutations (VAF > 50%) identified in KICH by our pipeline vs. those by LR-PCR method................. **p.26**

Supplementary Table 3. Somatic mtDNA nuclear transfer (SMNT) predicted to interrupt the open reading frames………………………………………….............. **p.28**

Supplementary Table 4. An overview of the mtDNA RNA-sequencing (RNA-seq) data... **p.31**

Supplementary Note

The ICGC/TCGA Pan-Cancer Analysis of Whole Genomes Consortium (PCAWG)……………………………………………………………………...… **p.32**

Supplementary Figure 1

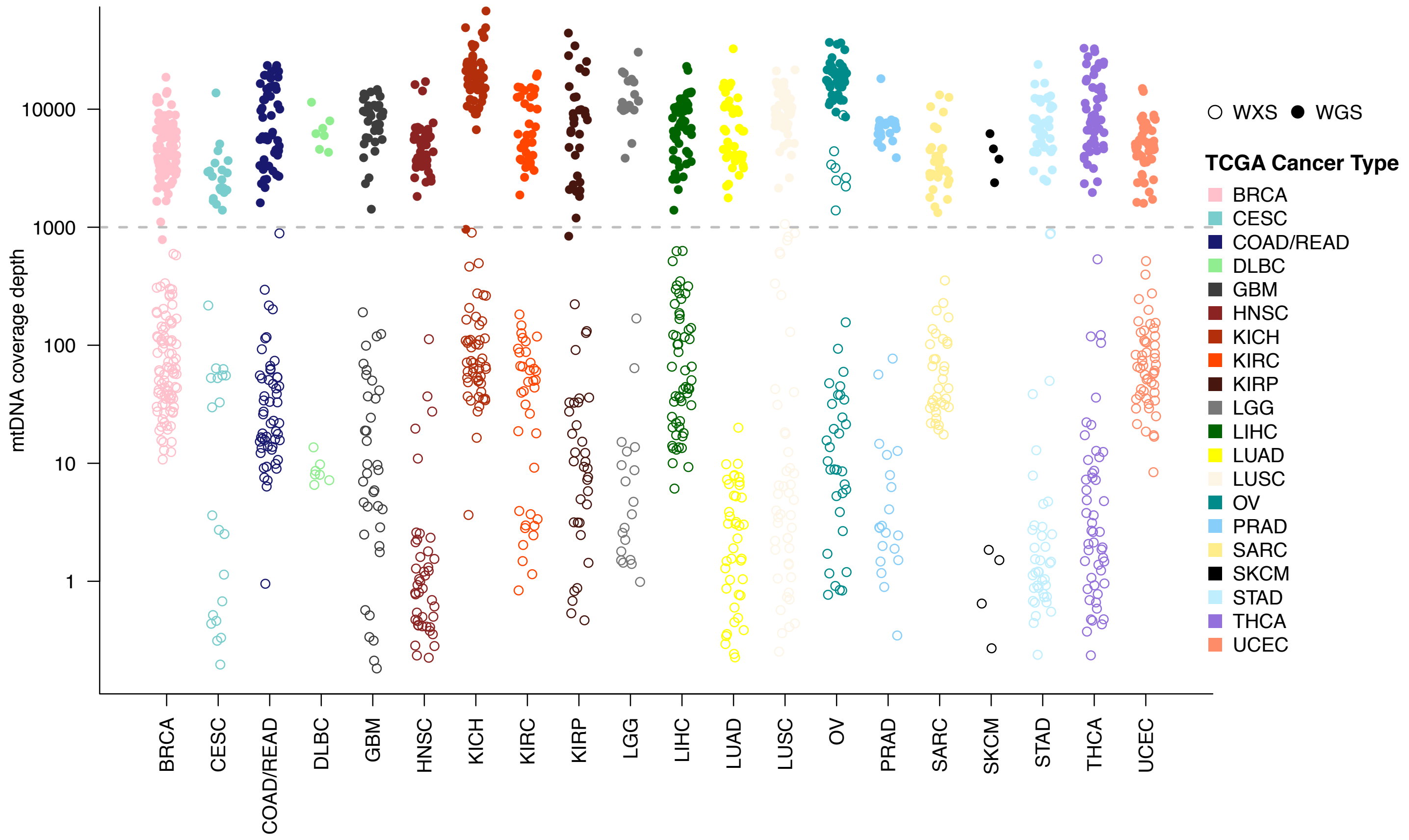

Comparison of mtDNA coverage depth for samples with both WGS and WGS data

Supplementary Figure 2

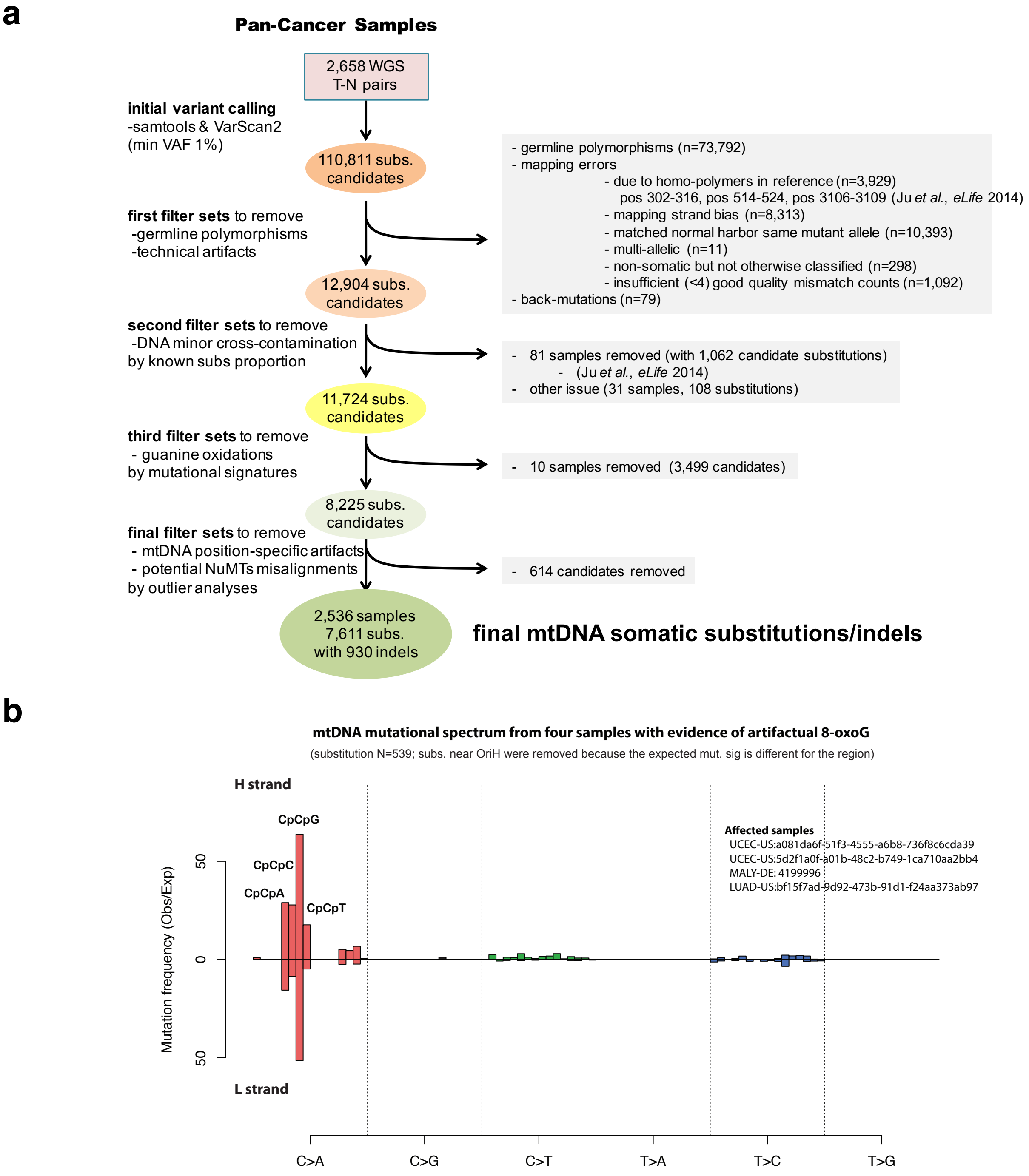

Filters to obtain sensitive and specific mtDNA somatic mutations
(a) A schematic diagram which summarizes the filters used in variant calling in this study. (b) Mutational spectrum from mutation candidates from four samples with evidence of artifactual guanine oxidation during library preparation step. The mutation spectrum is not consistent with that of mtDNA somatic mutations, but that of false positives due to artifacts (8-oxoGuanine) generated during library preparation steps (Costello et al., Nuc Acids Res 2012).

Supplementary Figure 3

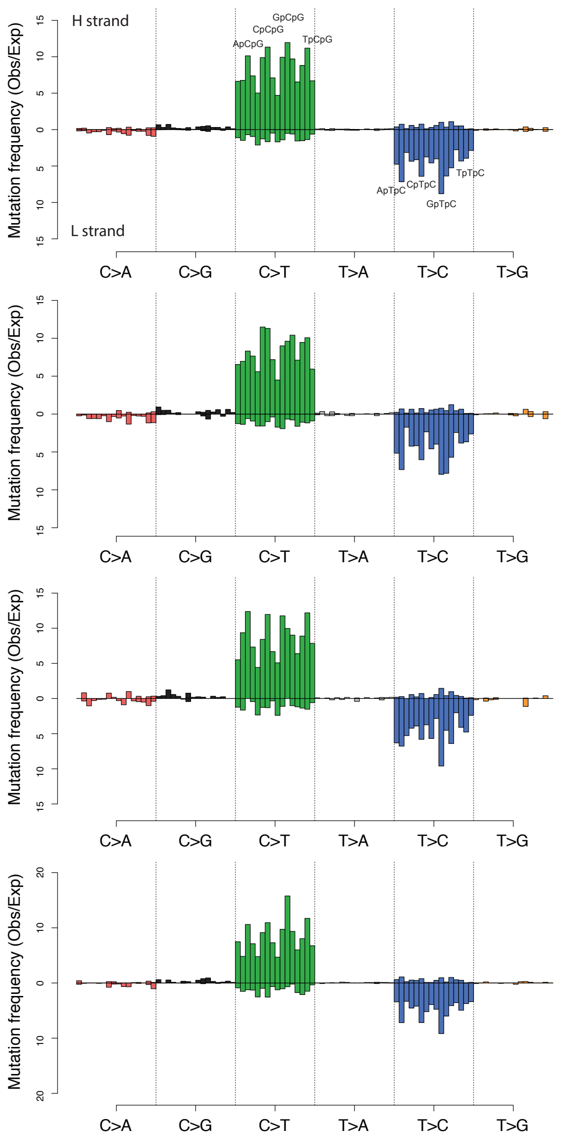

a **All mtDNA somatic substitutions**
(n=4,982 ; subs. near OriH were removed
because the expected mut. sig is different for the region)

b **mtDNA somatic substitutions with VAFs between [0.01, 0.03)**
(n=1,958 ; subs. near OriH were removed
because the expected mut. sig is different for the region)

c **mtDNA somatic substitutions with VAFs between [0.03, 0.10)**
(n=1,168 ; subs. near OriH were removed
because the expected mut. sig is different for the region)

d **mtDNA somatic substitutions with VAFs between [0.10, 1.00]**
(n=1,856; subs. near OriH were removed
because the expected mut. sig is different for the region)

Consistent mtDNA mutation spectrum for substitutions at VAF between 1%-3%
We compared mutation signatures for four different groups of substitutions: ones at VAF 1%-3%, 3%-10%, 10%-100% and overall (1%-100%). The 1%-3% VAF somatic substitutions (n=1,958; substitutions at D-Loop were not included) show unique mtDNA somatic mutation signature, suggesting they are mostly true positives.

Supplementary Figure 4

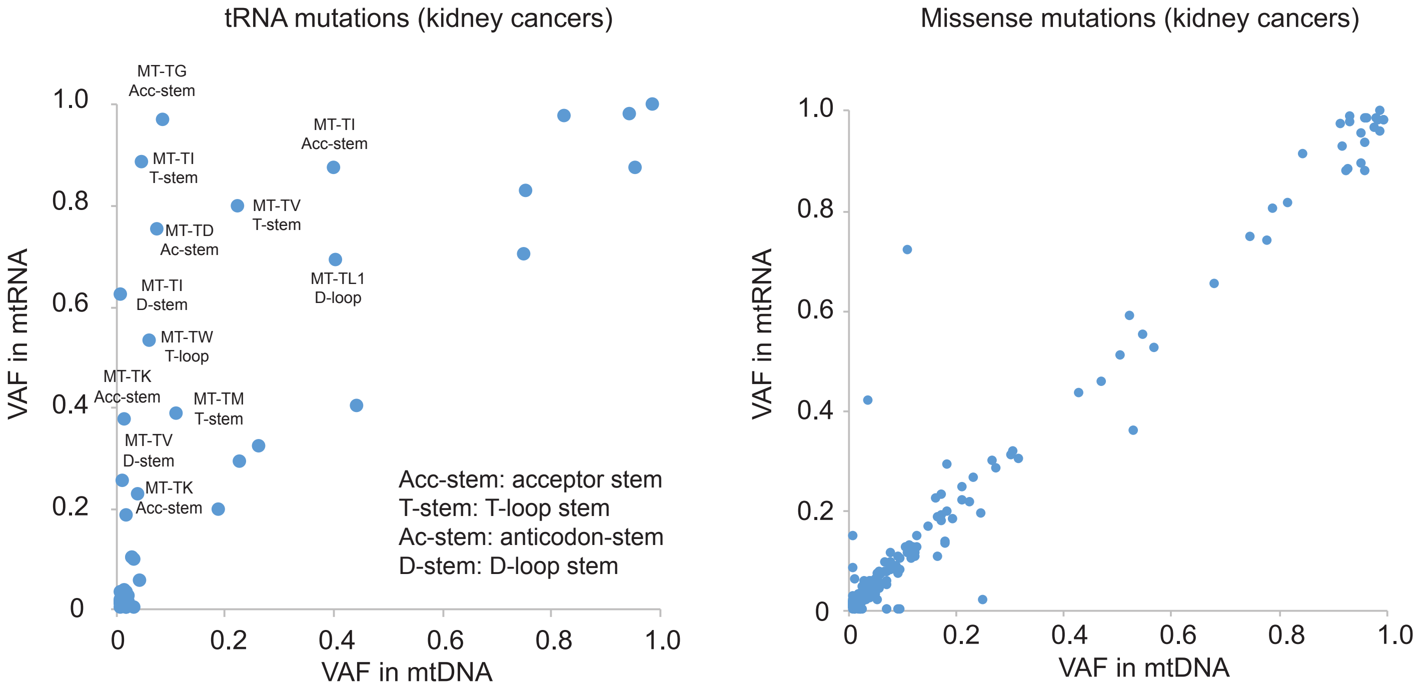

Comparison of mtDNA somatic mutations at DNA and mRNA level
Left panel: the variant allele frequency (VAF) of the same mutation in mitochondrial tRNA and its DNA. Several mutations affecting the secondary structure of tRNA resulted in accumulation of mutant tRNA. Right Panel: missense mutations of mitochondrial coding genes demonstrated proportional relationship between mtDNA and mtRNA VAF.

Supplementary Figure 5

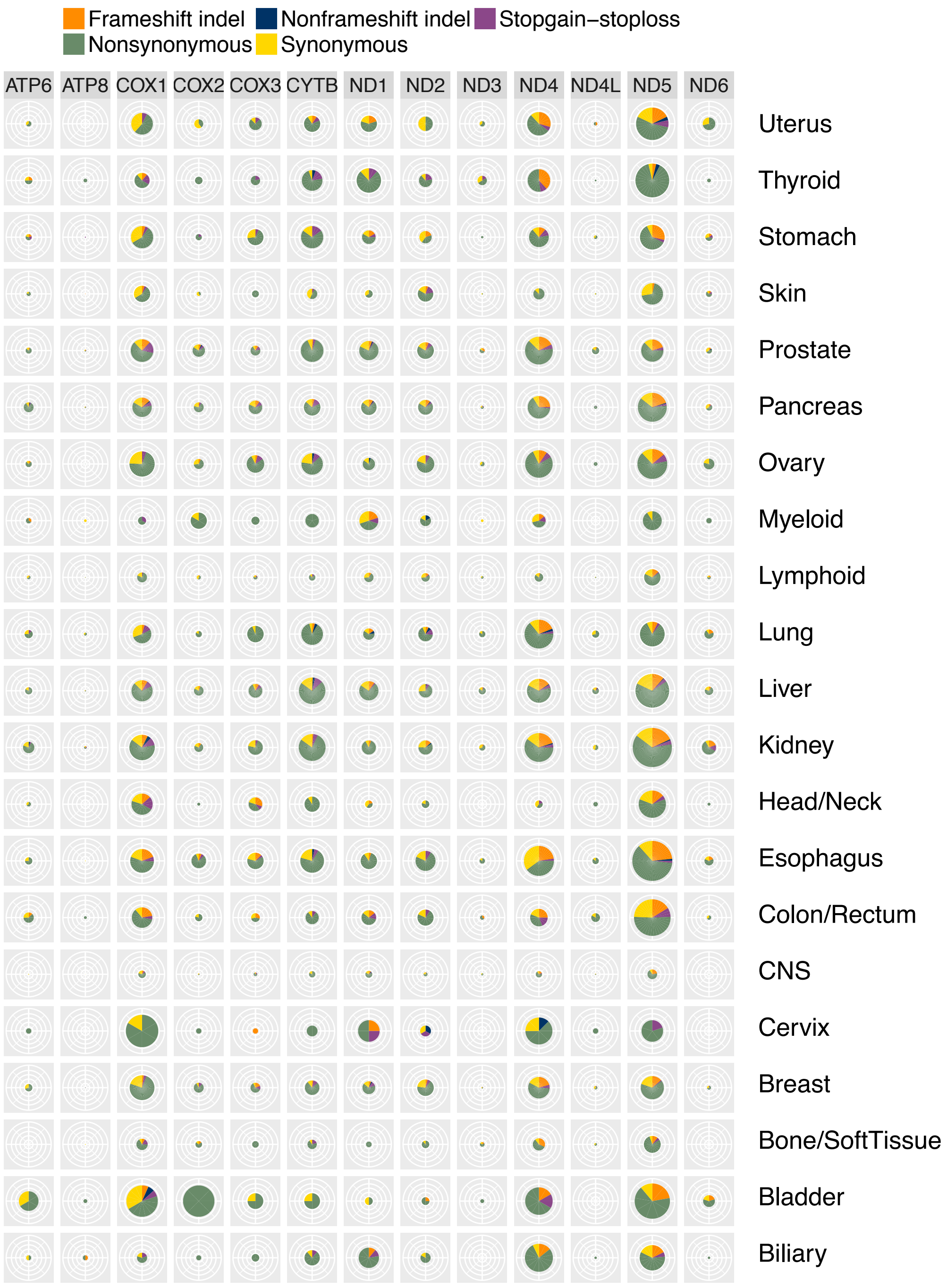

An overview of the mutation frequency of the 13 mtDNA coding genes across cancer tissue types
Pie chart sizes are proportional to overall mutation frequency; slice colors correspond to variant types.

Supplementary Figure 6

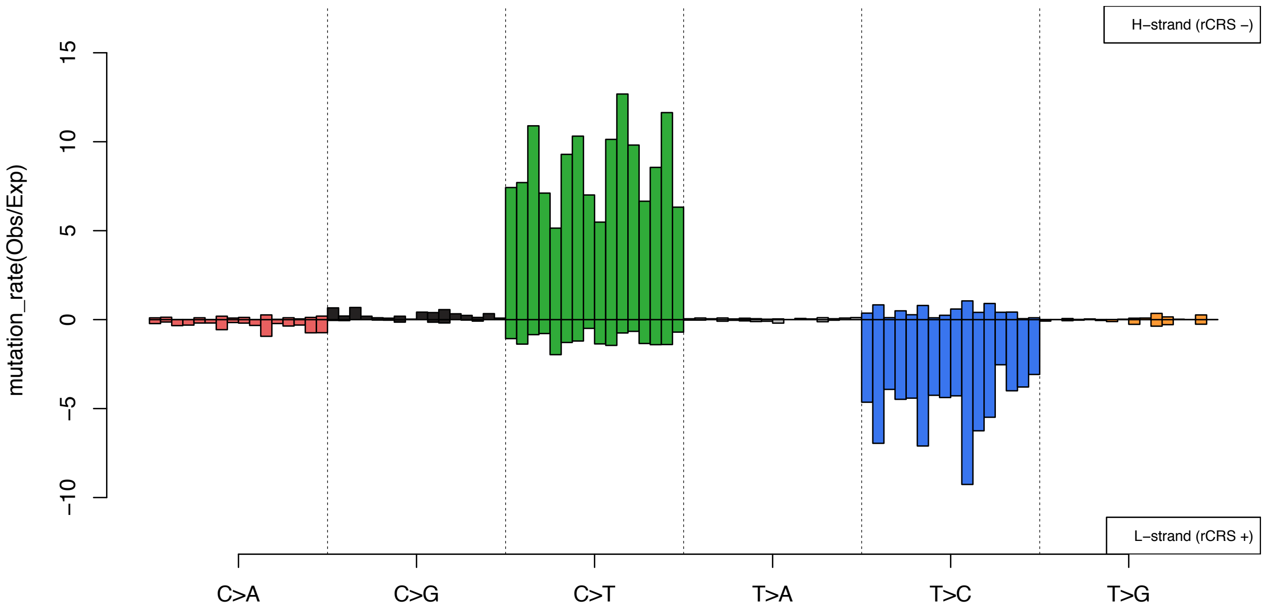

Replicative strand-specific mutational spectrum of mtDNA substitutions

Supplementary Figure 7

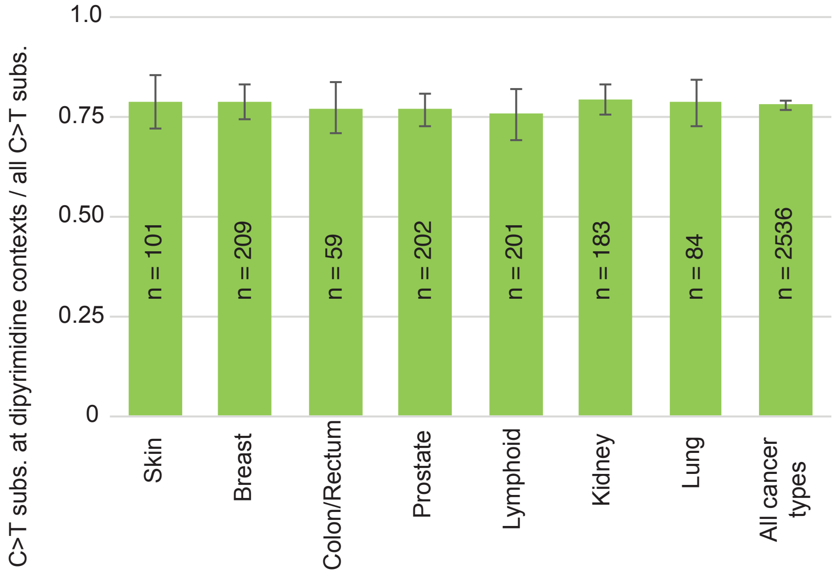

The proportion of C→T substitutions at dipyrimidine contexts (where UV light frequently generates mutations)
n = number of samples with mtDNA mutation information available. The error bars represent the 95% confidence intervals of the proportions calculated using binomial distribution.

Supplementary Figure 8

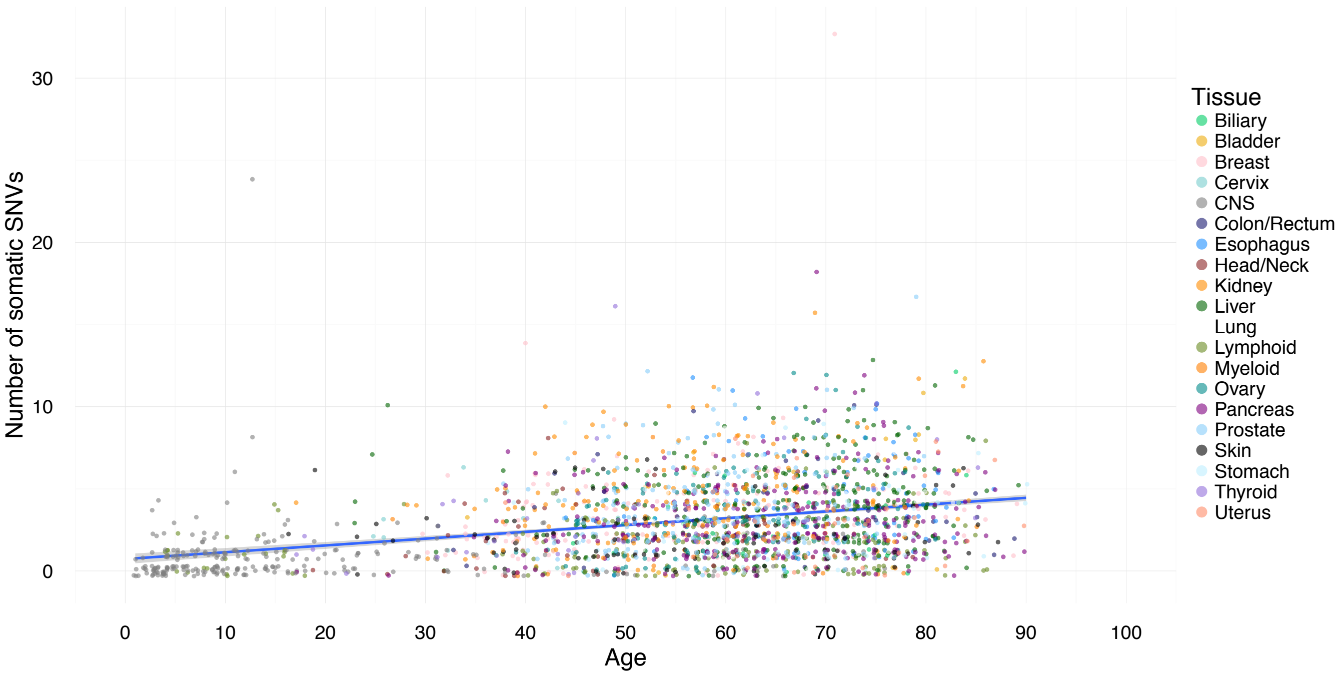

The correlation between the number of mtDNA somatic SNVs and patient age
The correlation was determined based on the 2,414 patients with both age and somatic SNV information available. The shaded region represents the 95% confidence interval for the predictions from a linear model with the number of somatics VAFs as the response variable and patient age as the explanatory variable.

Supplementary Figure 9

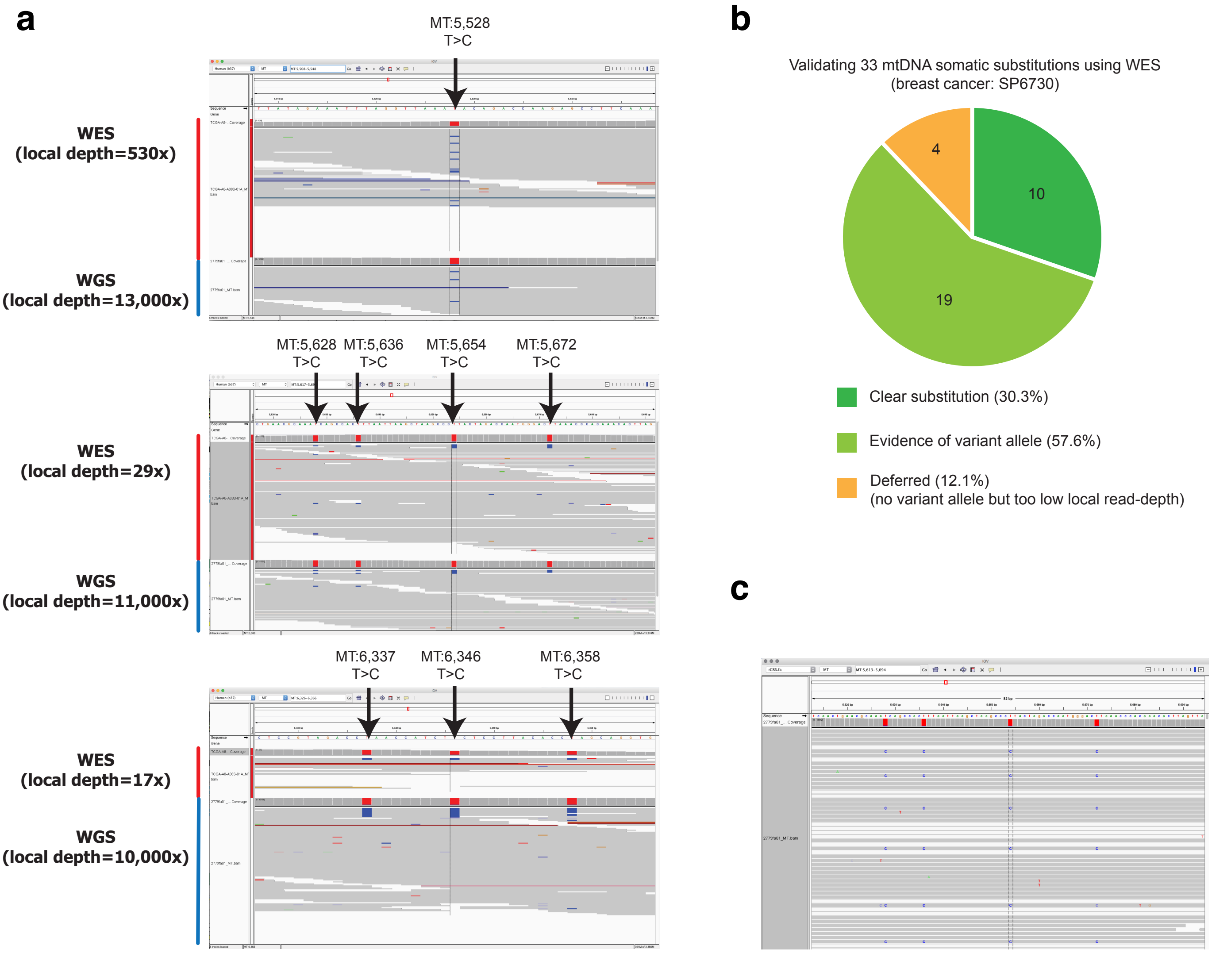

Hypermutated sample validated by WXS

Supplementary Figure 10

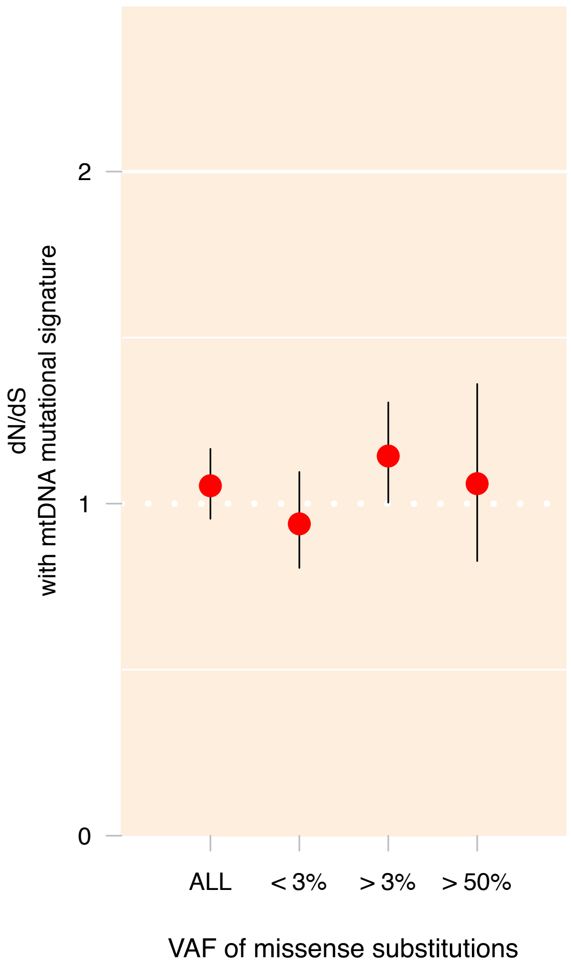

dN/dS ratio from mtDNA somatic missense substitutions
All VAF: 3521 missense vs 874 silent; VAF <3%: 1392 missense vs 366 silent ;VAF >3% 2129 missense vs 508 silent ; VAF >50%: 565 missense vs 132 silent mutations. The error bars represent the 95% confidence intervals of the dN/dS ratio (likelihood).

Supplementary Figure 11

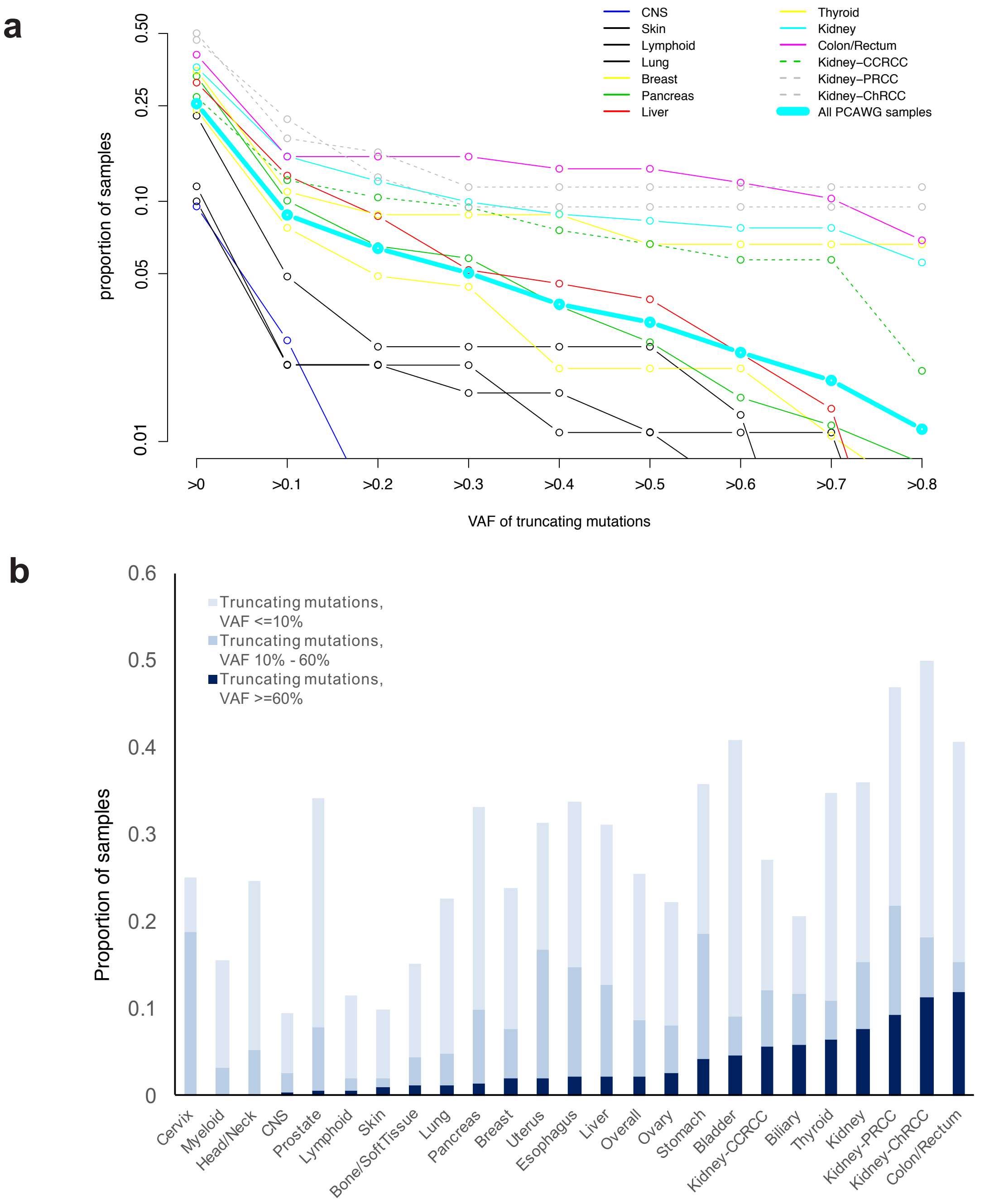

The proportion of samples with truncating mutations by cancer types
(a) The proportion of samples (y-axis) that harbor truncating mutations with VAF above specified cutoff (x-axis) within each cancer type. (b) The proportion of samples that harbor truncating mutations, grouped (in color) by their maximum VAF (<=10%, 10%-60%, >=60%) within each cancer type.

Supplementary Figure 12

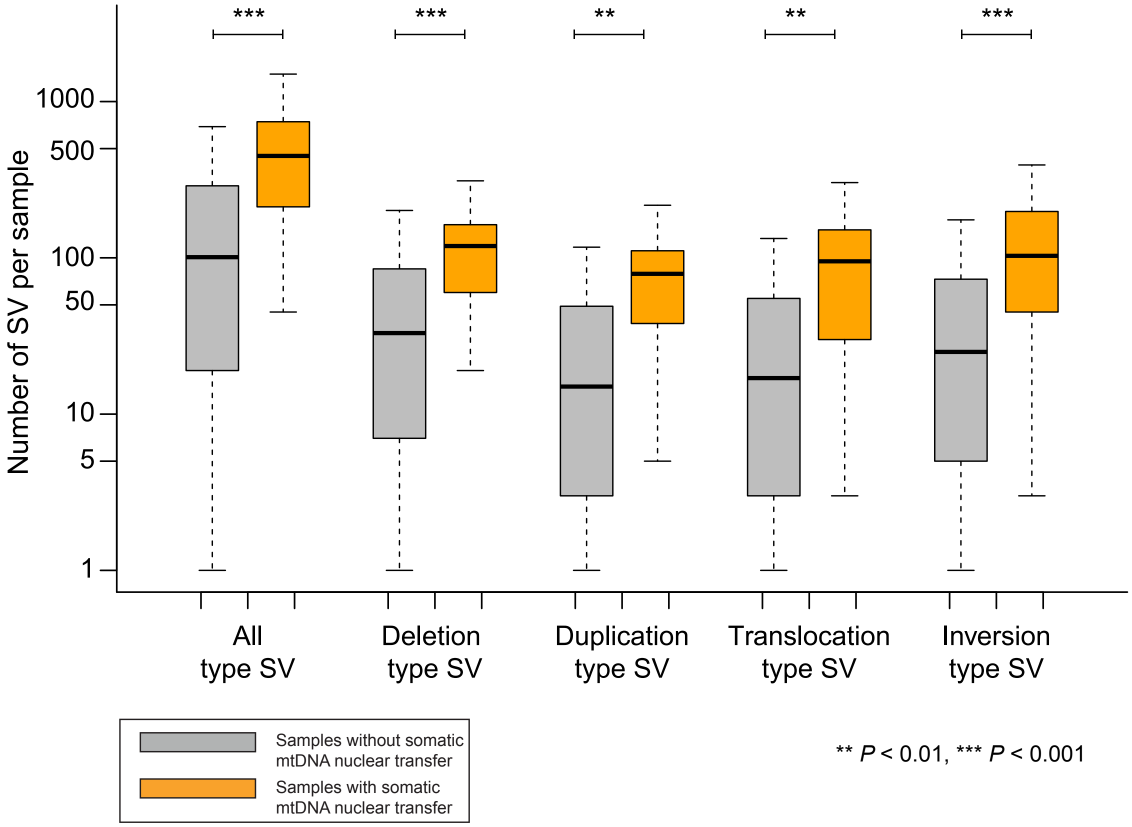

The numbers of SV breakpoints in samples with and without somatic mtDNA nuclear transfers (SMNT) by SV types

Samples without mtDNA transfer (gray) = 2603, with mtDNA transfer (orange) = 55. The p-values were based on two-sided t -test without multiple comparison adjustment. The boundaries of the boxes mark the first and third quartile, with the median in the center, and whiskers extending to 1.5 interquartile range from the boundaries.

Supplementary Figure 13

a

Breast
e4fc0909-f284-4471-866d-d8967b6adcbc

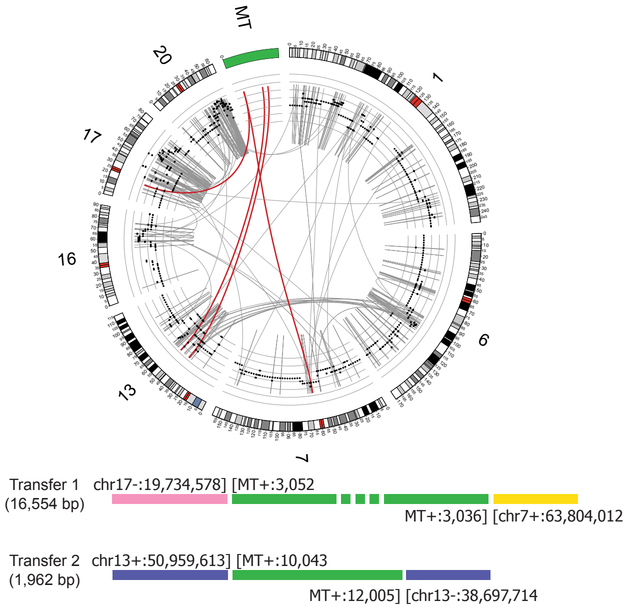

b

Lung (squamous)
e4fc0909-f284-4471-866d-d8967b6adcbc

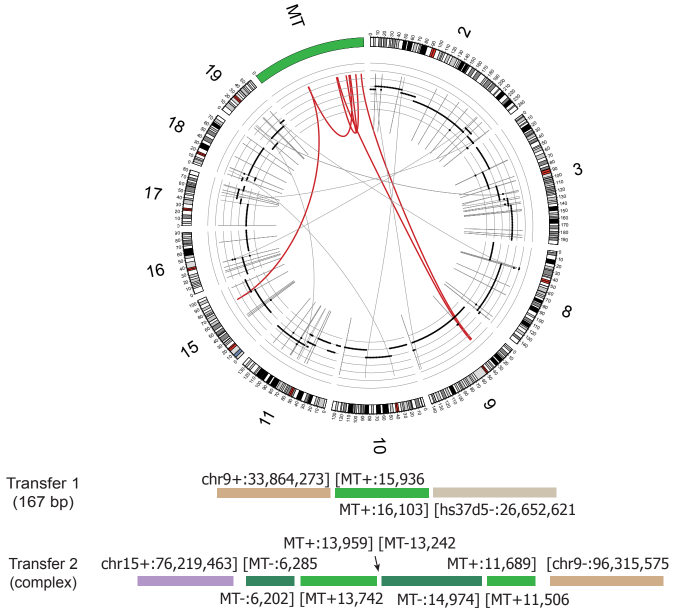

Somatic nuclear transfer of mtDNA

(a) A Circos plot representing two long-segment mtDNA nuclear transfers in a breast cancer genome. (b) A Circos plot representing two mtDNA nuclear transfers in a lung cancer genome. One mtDNA segment transferred has three inversion-type rearrangements as depicted.

Supplementary Figure 14

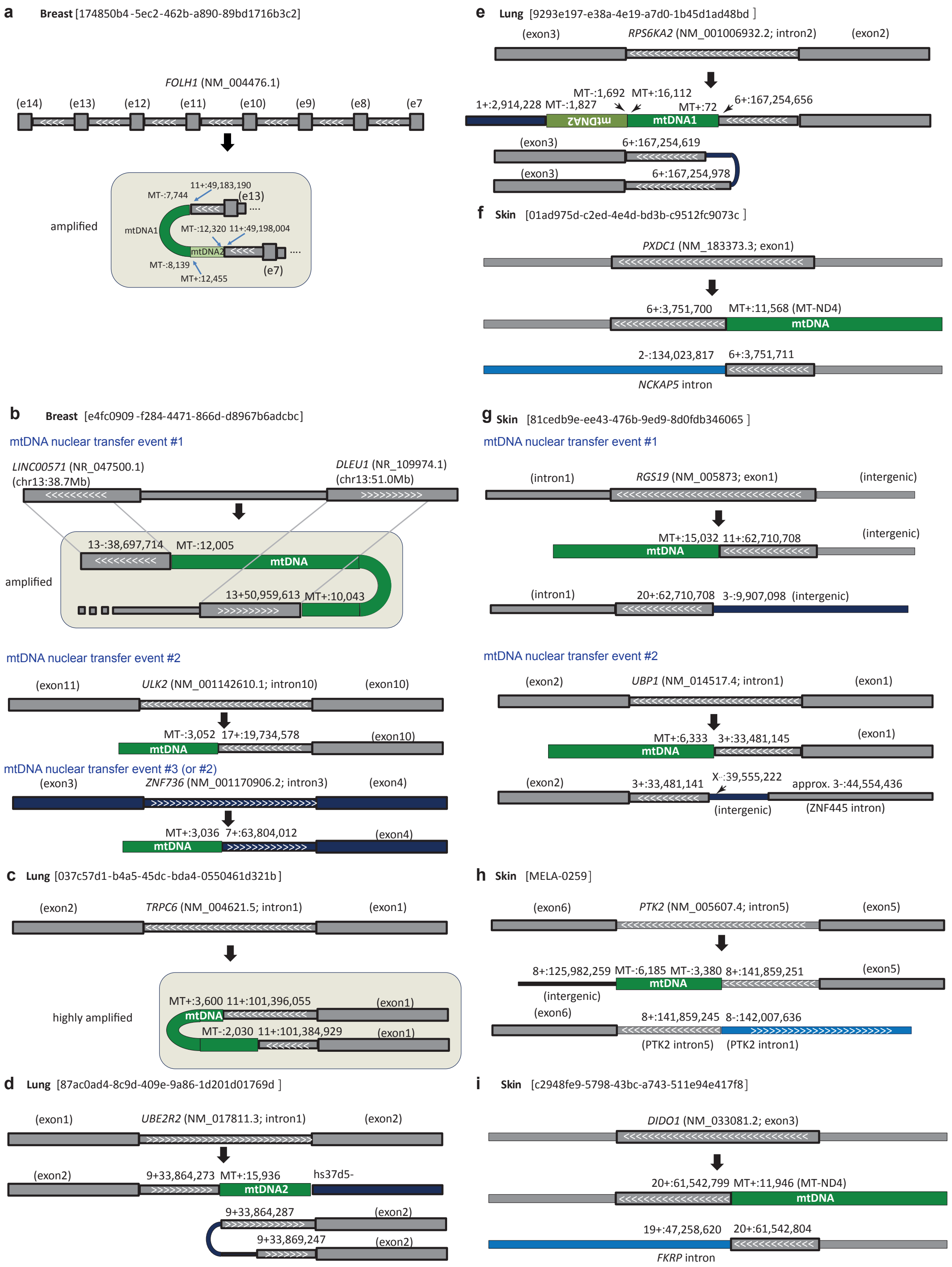

The functional consequence of the SMNT events

Supplementary Figure 15

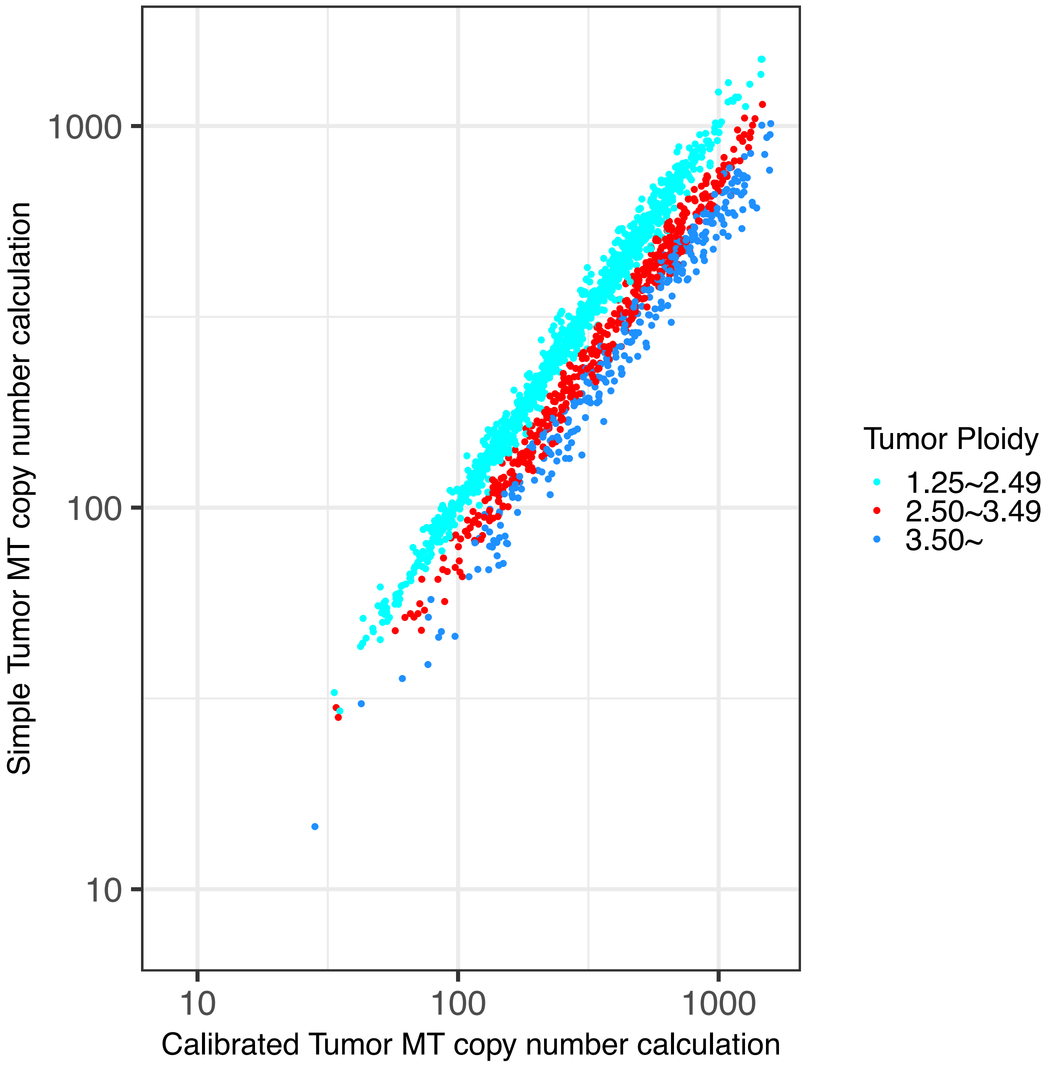

The comparison of mtDNA copy number calculated from simple estimation (using only coverage depth information) vs. our method (incorporating coverage depth, tumor purity and ploidy)

Supplementary Figure 16

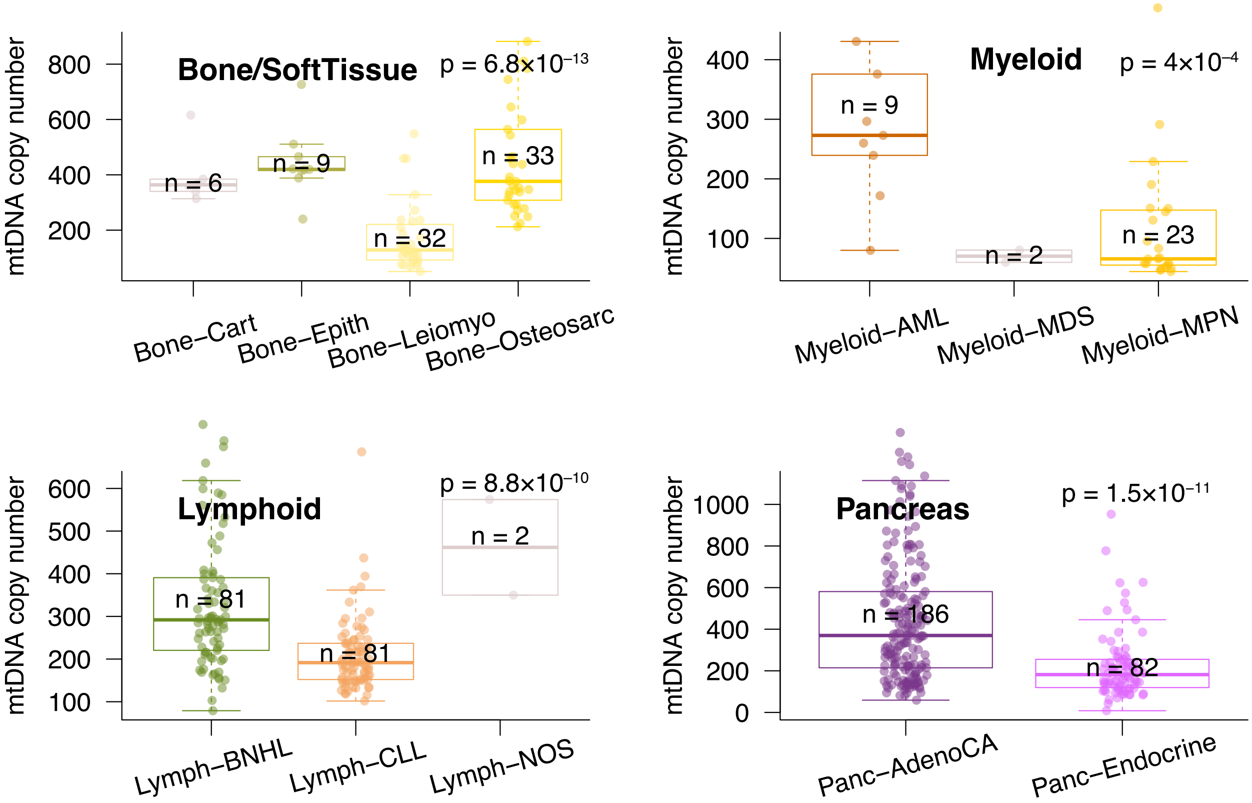

The copy number distributions of mtDNA in multiple cancers derived from the same tissue type
n = number of samples with cancer mtDNA information available. The p-values were either generated by t-test (for two cancer types) or ANOVA (for > two cancer types).The boundaries of the boxes mark the first and third quartile, with the median in the center, and whiskers extending to 1.5 interquartile range from the boundaries.

Supplementary Figure 17

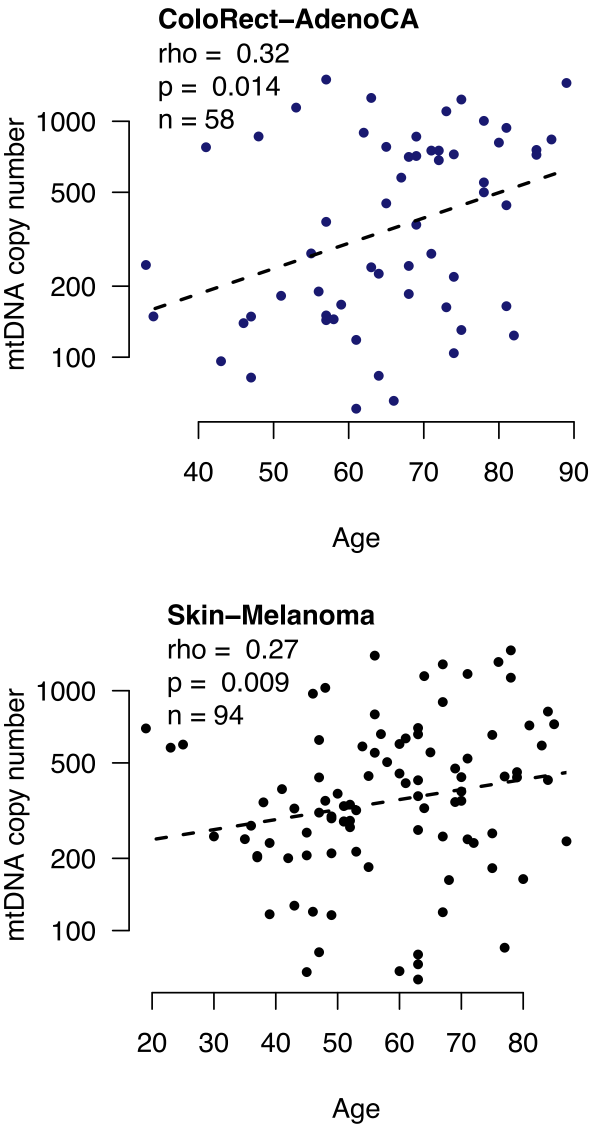

The correlation between mtDNA copy number and patient age
n = number of samples with cancer mtDNA copy number and patient age information available. The correlation and p-value were determined using Spearman's rank correlation.

Supplementary Figure 18

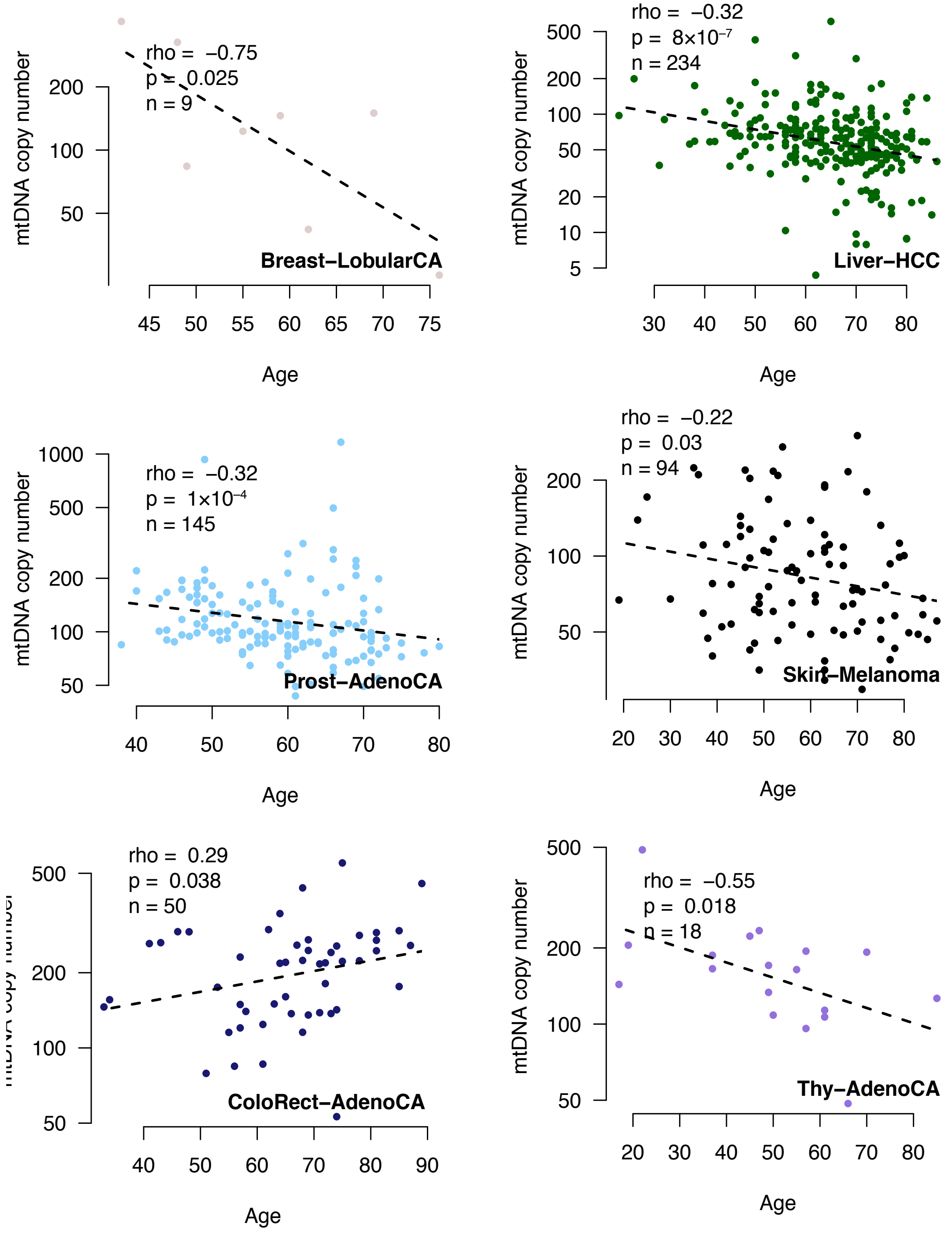

The correlation between normal blood mtDNA copy number and patient age
n = number of samples with normal blood mtDNA copy number and patient age information available. The correlation and p-value were determined using Spearman's rank correlation.

Supplementary Figure 19

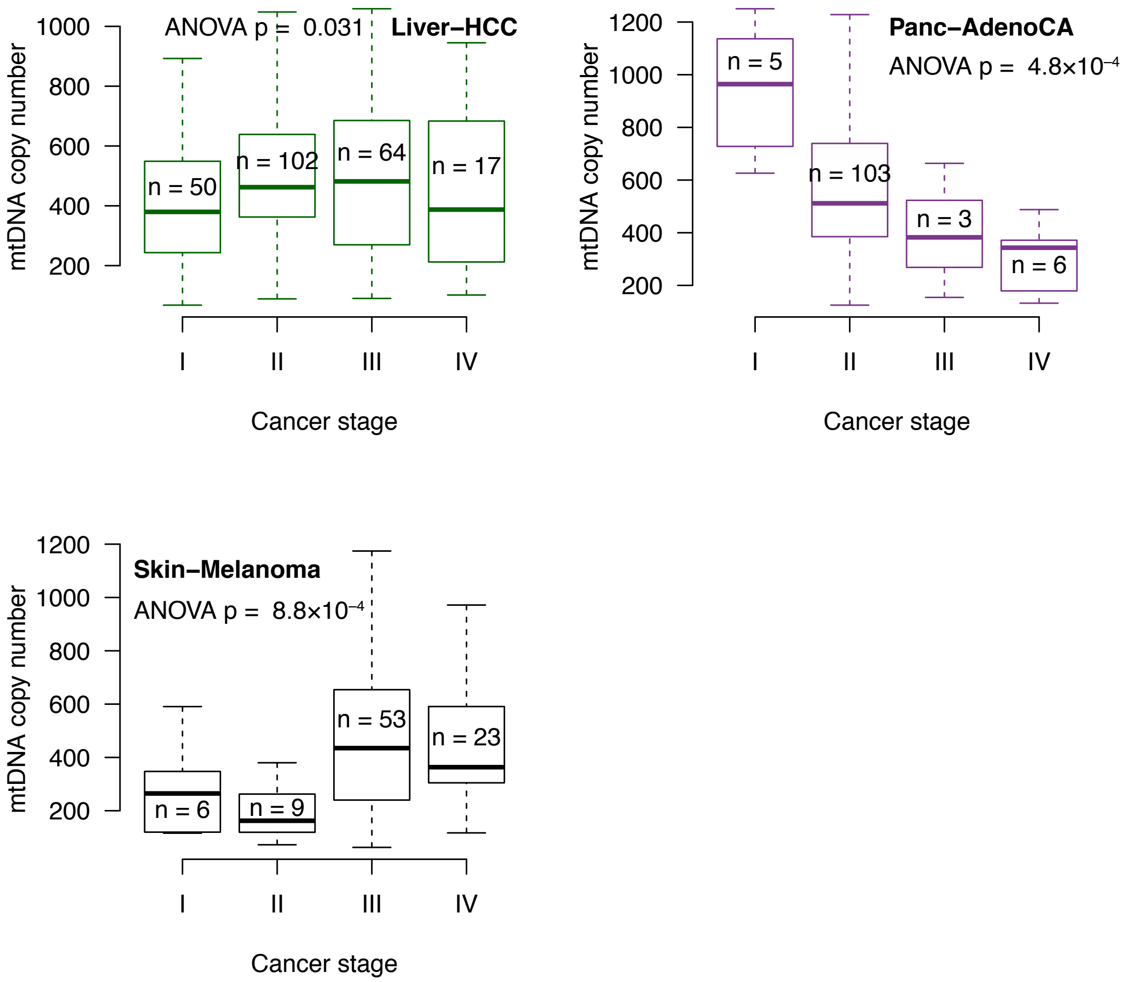

The correlation between mtDNA copy number and patient cancer stage
n = number of samples with cancer mtDNA copy number and stage information available. the boundaries of the boxes mark the first and third quartile, with the median in the center, and whiskers extending to 1.5 interquartile range from the boundaries.

Supplementary Figure 20

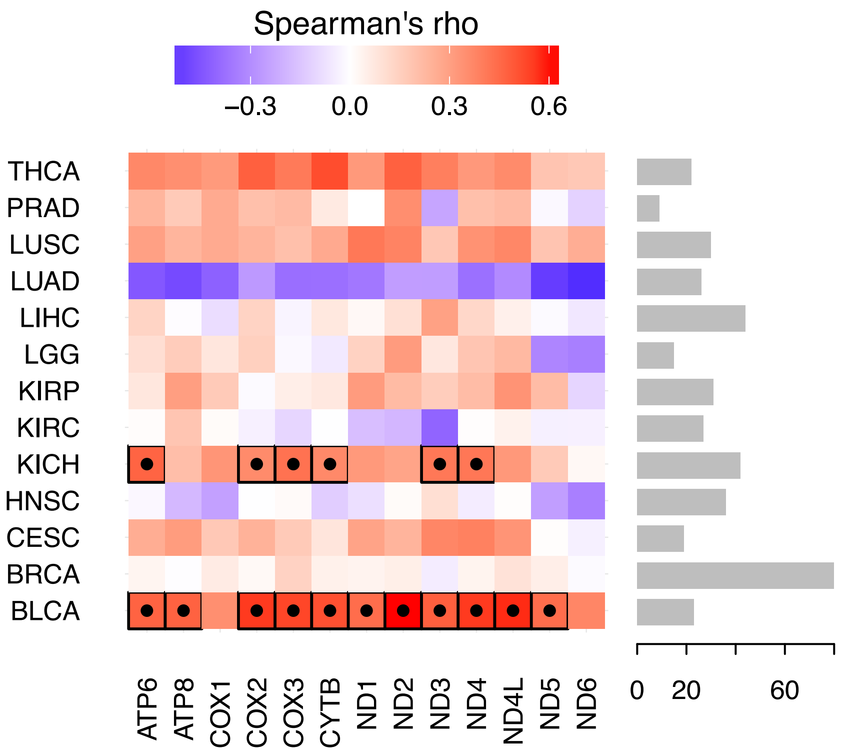

The correlation between mtDNA copy number and gene expression.
The left panel shows the heatmap of Spearman’s rho, with the significant cases (with FDR < 0.05) boxed and marked with black dots. The right panel shows the sample sizes (the number of samples with both mtDNA copy number and gene expression data available for each cancer type) in barplot.

Supplementary Figure 21

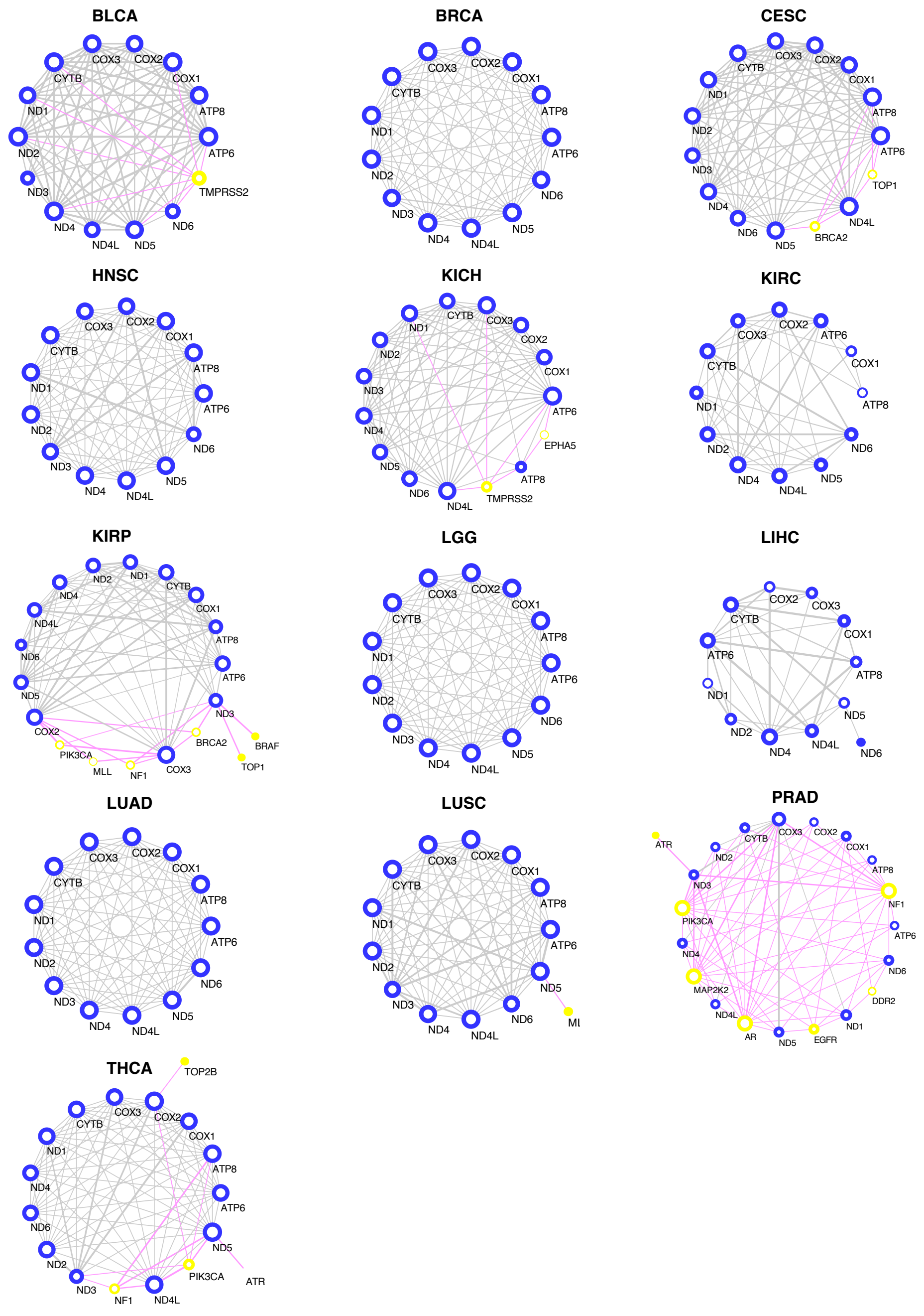

The gene co-expression network seeded on mtDNA genes

The size of the node is proportional to the number its direct neighbors (connectivity). The thickness of the edge is proportional to the connection strength between nodes. Further, the nodes were colored based on the gene: mtDNA genes in blue, nuclear genes in yellow; the edges were colored based on the connection type: mtDNA gene - mtDNA gene connection in gray, mtDNA gene - nuclear gene connection in magenta.

Supplementary Figure 22

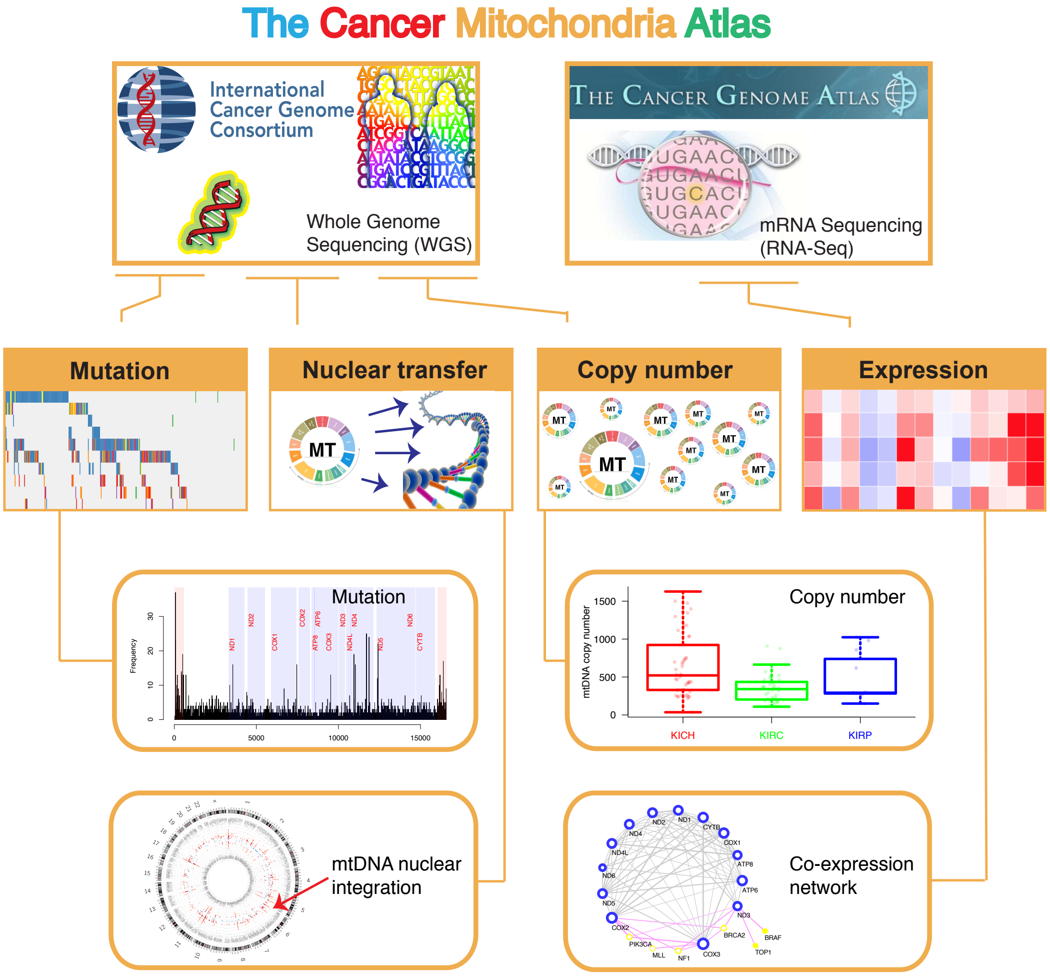

An overview of the TCMA data portal

Supplementary Table 1. An overview of the mtDNA whole-genome sequencing (WGS) data

Cancer tissue	Cancer (ICGC)	Sample #	Average mtDNA coverage
**Biliary**	Biliary-AdenoCA	34	10591.4
**Bladder**	Bladder-TCC	23	3201.2
**Bone/SoftTissue**	Bone-Benign, Bone-Epith, Bone-Osteosarc, SoftTissue-Leiomyo, SoftTissue-Liposarc	98	8160.8
**Breast**	Breast-AdenoCA, Breast-DCIS, Breast-LobularCA	214	9724.1
**Cervix**	Cervix-AdenoCA, Cervix-SCC	20	3211.9
**CNS**	CNS-GBM, CNS-Medullo, CNS-Oligo, CNS-PiloAstro	294	5766.3
**Colon/Rectum**	ColoRect-AdenoCA	60	10374.3
**Esophagus**	Eso-AdenoCA	98	6546.4
**Head/Neck**	Head-SCC	57	6765.3
**Kidney**	Kidney-ChRCC (Kidney-Chromophobe), Kidney-RCC (Kidney-Clear Cell, Kidney-Papillary)	189	13213.0
**Liver**	Liver-HCC	317	11542.0
**Lung**	Lung-AdenoCA, Lung-SCC	86	9146.9
**Lymphoid**	Lymph-BNHL, Lymph-CLL	202	5289.8
**Myeloid**	Myeloid-AML, Myeloid-MDS, Myeloid-MPN	38	3706.1
**Ovary**	Ovary-AdenoCA	113	16960.4
**Pancreas**	Panc-AdenoCA, Panc-Endocrine	324	12629.9
**Prostate**	Prost-AdenoCA	210	14533.1
**Skin**	Skin-Melanoma	107	10812.5
**Stomach**	Stomach-AdenoCA	75	7014.7
**Thyroid**	Thy-AdenoCA	48	12104.8
**Uterus**	Uterus-AdenoCA	51	5313.7
21 tissue types	38 Cancer types	***2658**	**9959.1**

*Samples used in mutation analysis: 2,536; samples used in nuclear transfer analysis: 2,658; samples used in copy number analysis: 2,157.

Supplementary Table 2. The high-allele-frequency mtDNA mutations (VAF > 50%) identified in KICH by our pipeline vs. those by LR-PCR method

(a) All high-allele-frequency mtDNA mutations (VAF > 50%) identified in KICH by our pipeline can be validated by LR-PCR method

TCGA barcode	Chr	Substitution /Indel	Position	Ref	Var	Region	VAF	In LR-PCR results
**TCGA-KL-8327**	MT	Indel	65	T	+G	D-Loop	59%	Yes
**TCGA-KL-8328**	MT	Substitution	16355	C	T	D-Loop	72%	Yes
**TCGA-KL-8328**	MT	Substitution	16399	A	G	D-Loop	57%	Yes
**TCGA-KL-8333**	MT	Substitution	5767	C	T	TRNC	75%	Yes
**TCGA-KL-8341**	MT	Substitution	14159	C	A	ND6	78%	Yes
**TCGA-KL-8344**	MT	Indel	13127	A	-C	ND5	91 %	Yes
**TCGA-KM-8438**	MT	Indel	65	T	+G	D-Loop	87%	Yes
**TCGA-KM-8438**	MT	Substitution	4569	G	A	ND2	82%	Yes
**TCGA-KM-8438**	MT	Substitution	5738	G	C	L-strand origin	83%	Yes
**TCGA-KM-8441**	MT	Indel	13230	C	-A	ND5	88%	Yes
**TCGA-KM-8477**	MT	Substitution	4429	G	A	TRNM	99%	Yes
**TCGA-KM-8477**	MT	Substitution	4969	G	C	ND2	99%	Yes
**TCGA-KM-8477**	MT	Substitution	10806	G	A	ND4	99%	Yes
**TCGA-KM-8477**	MT	Indel	12384	T	-C	ND5	80%	Yes
**TCGA-KN-8419**	MT	Indel	12417	C	+A	ND5	82%	Yes
**TCGA-KN-8422**	MT	Indel	65	T	+G	D-Loop	67%	Yes
**TCGA-KN-8432**	MT	Substitution	6490	T	C	COX1	96%	Yes
**TCGA-KN-8434**	MT	Substitution	3922	G	A	ND1	68%	Yes
**TCGA-KN-8434**	MT	Substitution	9651	C	T	COX3	80%	Yes
**TCGA-KN-8435**	MT	Substitution	16156	G	A	D-Loop	53%	Yes
**TCGA-KN-8437**	MT	Indel	13230	C	-A	ND5	90%	Yes

(b). Our pipeline captured 21/26 of the high-allele-frequency mtDNA mutations (VAF > 50%) previously identified in KICH by LR-PCR method

TCGA barcode	Chr	Substitution /Indel	Position	Ref	Var	Region	VAF	In WGS results
**TCGA-KL-8326**	MT	Indel	11866	A	+C	ND4	82%	Filtered
**TCGA-KL-8327**	MT	Indel	65	T	+G	D-Loop	59%	Yes
**TCGA-KL-8331**	MT	Indel	451	A	-T	D-Loop	51%	Yes
**TCGA-KL-8331**	MT	Substitution	16426	C	A	D-Loop	51%	No
**TCGA-KL-8333**	MT	Substitution	5767	C	T	TRNC	73%	Yes
**TCGA-KL-8341**	MT	Substitution	14159	C	A	ND6	81%	Yes
**TCGA-KL-8343**	MT	Indel	3565	A	+C	ND1	79%	No
**TCGA-KL-8344**	MT	Indel	13127	A	-C	ND5	86%	Yes
**TCGA-KM-8438**	MT	Indel	65	T	+G	D-Loop	93%	Yes
**TCGA-KM-8438**	MT	Substitution	4569	G	A	ND2	82%	Yes
**TCGA-KM-8438**	MT	Substitution	5738	G	C	L-strand origin	86%	Yes
**TCGA-KM-8441**	MT	Indel	13230	C	-A	ND5	90%	Yes

**TCGA-KM-8442**	MT	Indel	3105	A	-C	RNR2	93%	No
**TCGA-KM-8476**	MT	Substitution	16426	C	A	D-Loop	51%	No
**TCGA-KM-8477**	MT	Substitution	4429	G	A	TRNM	99%	Yes
**TCGA-KM-8477**	MT	Substitution	4969	G	C	ND2	99%	Yes
**TCGA-KM-8477**	MT	Substitution	10806	G	A	ND4	99%	Yes
**TCGA-KM-8477**	MT	Indel	12384	T	-C	ND5	76%	Yes
**TCGA-KN-8419**	MT	Indel	12417	C	+A	ND5	72%	Yes
**TCGA-KN-8422**	MT	Indel	65	T	+G	D-Loop	71%	Yes
**TCGA-KN-8428**	MT	Substitution	1900	A	G	RNR2	93%	No
**TCGA-KN-8432**	MT	Substitution	6490	T	C	COX1	95%	Yes
**TCGA-KN-8434**	MT	Substitution	3922	G	A	ND1	67%	Yes
**TCGA-KN-8434**	MT	Substitution	9651	C	T	COX3	76%	Yes
**TCGA-KN-8435**	MT	Substitution	16156	G	A	D-Loop	51%	Yes
**TCGA-KN-8437**	MT	Indel	13230	C	-A	ND5	89%	Yes

Supplementary Table 3. Somatic mtDNA nuclear transfer (SMNT) predicted to interrupt the open reading frames

Sample	MT Chr	MT Strand	MT Position	Nuclear Chr	Nuclear Strand	Nuclear Position	Orientation (1: MT-nuclear, 0: nuclear-MT)	Genes	Genetic impact
BLCA:9c274536-3ca1-4f0e-93a8-1688074d862f	MT	+	16,525	3	+	161,285,798	1	intergenic	-
BLCA:9c274536-3ca1-4f0e-93a8-1688074d862f	MT	+	16,113	10	+	131,401	0	intergenic	-
BLCA:9c274536-3ca1-4f0e-93a8-1688074d862f	MT	+	11,468	11	-	67,592,693	1	intergenic	-
BLCA:9c274536-3ca1-4f0e-93a8-1688074d862f	MT	+	11,140	11	-	67,593,978	0	intergenic	-
BLCA:9c274536-3ca1-4f0e-93a8-1688074d862f	MT	+	16,115	15	-	93,433,461	0	CHD2(intron)	not remarkable
BLCA:9c274536-3ca1-4f0e-93a8-1688074d862f	MT	+	27	15	-	93,433,460	1	CHD2(intron)	not remarkable
BOCA:CGP_donor_1691150	MT	+	16,106	4	+	82,052,576	0	PRKG2(intron)	indeterminable
BOCA:CGP_donor_1691150	MT	+	16,108	20	+	51,370,057	0	intergenic	-
BOCA:CGP_donor_1691217	MT	+	14,405	9	+	22,816,364	1	FLJ35282(intron)	indeterminable
BRCA:174850b4-5ec2-462b-a890-89bd1716b3c2	MT	+	12,320	11	-	49,198,004	0	FOLH1(intron)	truncation
BRCA:174850b4-5ec2-462b-a890-89bd1716b3c2	MT	-	7,744	11	+	49,183,190	1	FOLH1(intron)	truncation
BRCA:4da999a0-ef41-4a0b-b1d1-446b39cc855a	MT	+	1,358	8	+	37,433,689	0	intergenic	-
BRCA:4da999a0-ef41-4a0b-b1d1-446b39cc855a	MT	+	2,838	16	+	11,905,542	1	intergenic	-
BRCA:a9b7d7fe-be31-4f71-afee-c1bfdf511888	MT	+	1,579	3	+	11,755,134	0	VGLL4(intron)	not remarkable
BRCA:a9b7d7fe-be31-4f71-afee-c1bfdf511888	MT	+	1,671	16	+	64,385,169	1	intergenic	-
BRCA:b094e8b2-8ece-4b36-8025-18073a8b873c	MT	-	3,284	3	+	51,848,724	0	IQCF1(intron)	truncation
BRCA:b094e8b2-8ece-4b36-8025-18073a8b873c	MT	-	846	3	-	51,848,760	1	IQCF1(intron)	truncation
BRCA:b094e8b2-8ece-4b36-8025-18073a8b873c	MT	+	9,775	3	+	84,640,033	0	intergenic	
BRCA:b094e8b2-8ece-4b36-8025-18073a8b873c	MT	+	10,364	3	+	84,640,058	1	intergenic	
BRCA:c0892598-1f7b-4f23-9cd8-731f797753d5	MT	+	3,711	1	-	163,302,499	0	NUF2(intron)	truncation
BRCA:c0892598-1f7b-4f23-9cd8-731f797753d5	MT	+	80	16	+	61,521,078	1	intergenic	
BRCA:CGP_donor_1232859	MT	+	109	X	-	17,546,722	1	NHS(intron)	indeterminable
BRCA:CGP_donor_1337226	MT	+	16,247	15	+	53,349,620	0	intergenic	-
BRCA:CGP_donor_1337226	MT	+	16,455	15	+	53,349,509	1	intergenic	-
BRCA:CGP_donor_1353432	MT	+	16,127	7	-	96,923,230	0	intergenic	-
BRCA:CGP_donor_1353432	MT	+	12,736	X	-	14,944,763	1	intergenic	-
BRCA:CGP_donor_1353432	MT	+	63	7	-	96,680,019	0	intergenic	-
BRCA:CGP_donor_1363963	MT	+	10,060	8	+	89,726,912	0	intergenic	-
BRCA:CGP_donor_1363963	MT	+	12,584	8	+	77,053,130	0	intergenic	-
BRCA:d67cd793-2931-429a-9084-2f3c4c8be7ad	MT	+	8,643	9	-	96,752,532	0	intergenic	-
BRCA:d67cd793-2931-429a-9084-2f3c4c8be7ad	MT	+	10,203	9	-	96,752,475	1	intergenic	-
BRCA:d8d7a6c2-6427-4f47-968c-6c3affba4617	MT	+	4,915	17	+	37,882,252	0	ERBB2(intron)	truncation
BRCA:d8d7a6c2-6427-4f47-968c-6c3affba4617	MT	+	5,344	17	+	37,871,155	1	ERBB2(intron)	truncation
BRCA:e4fc0909-f284-4471-866d-d8967b6adcbc	MT	+	10,043	13	+	50,959,613	0	DLEU1(intron)	fusion_transcript
BRCA:e4fc0909-f284-4471-866d-d8967b6adcbc	MT	+	12,005	13	-	38,697,714	1	LINC00751(intron)	fusion_transcript
BRCA:e4fc0909-f284-4471-866d-d8967b6adcbc	MT	+	3,052	17	-	19,734,578	0	ULK2(intron)	truncation
BRCA:e4fc0909-f284-4471-866d-d8967b6adcbc	MT	+	3,036	7	+	63,804,012	1	ZNF736(intron)	truncation
HNSC:4cffea0b-90a7-4c86-a73f-bb8feca3ada7	MT	+	92	11	+	113,976,595	1	ZBTB16(intron)	not remarkable
HNSC:7de19081-d5fd-468c-ad0d-f6e3e8b2ad70	MT	+	5,763	8	+	115,373,838	0	intergenic	-
HNSC:7de19081-d5fd-468c-ad0d-f6e3e8b2ad70	MT	+	5,804	8	-	115,373,630	1	intergenic	-

**HNSC:90a8d2cb-1f31-45f9-8575-eed3cb9a7798**	MT	+	16,454	9	+	137,950,628	0	intergenic	-
**HNSC:90a8d2cb-1f31-45f9-8575-eed3cb9a7798**	MT	+	1,403	15	+	101,262,248	1	intergenic	-
**LUAD:bf15f7ad-9d92-473b-91d1-f24aa373ab97**	MT	+	522	7	-	64,473,632	0	intergenic	-
**LUAD:cc4bd56a-25c5-4c48-b583-ac3aeb778ca6**	MT	+	1,931	14	+	40,799,718	1	intergenic	-
**LUAD:cc4bd56a-25c5-4c48-b583-ac3aeb778ca6**	MT	+	15,955	14	-	72,230,011	1	intergenic	-
**LUAD:cc4bd56a-25c5-4c48-b583-ac3aeb778ca6**	MT	+	15,196	14	-	72,439,511	0	intergenic	-
**LUAD:cc4bd56a-25c5-4c48-b583-ac3aeb778ca6**	MT	+	2,253	14	-	72,618,504	1	intergenic	-
**LUAD:cc4bd56a-25c5-4c48-b583-ac3aeb778ca6**	MT	+	11,325	14	-	72,618,020	1	intergenic	-
**LUAD:cc4bd56a-25c5-4c48-b583-ac3aeb778ca6**	MT	+	6,126	14	-	72,623,601	1	intergenic	-
**LUAD:cc4bd56a-25c5-4c48-b583-ac3aeb778ca6**	MT	+	3,898	14	-	72,626,341	0	intergenic	-
**LUAD:cc4bd56a-25c5-4c48-b583-ac3aeb778ca6**	MT	+	693	21	+	9,827,144	1	intergenic	-
**LUSC:037c57d1-b4a5-45dc-bda4-0550461d321b**	MT	+	2,030	11	-	101,384,929	0	TRPC6(intron)	truncation
**LUSC:037c57d1-b4a5-45dc-bda4-0550461d321b**	MT	+	3,600	11	+	101,396,055	1	TRPC6(intron)	truncation
**LUSC:220be009-8f1e-4a77-ab04-3220b5f87ea6**	MT	+	9,815	13	+	108,054,362	0	FAM155A(intron)	truncation
**LUSC:3edf933a-be1a-46e8-bfb0-acdff30e64a0**	MT	+	1,344	12	-	94,816,065	1	CCDC41(intron)	not remarkable
**LUSC:585e6487-b0a3-4828-8a06-46bee01dff74**	MT	+	16,110	1	+	84,517,869	0	intergenic	-
**LUSC:585e6487-b0a3-4828-8a06-46bee01dff74**	MT	+	49	21	+	22,236,786	1	intergenic	-
**LUSC:87ac0ad4-8c9d-409e-9a86-1d201d01769d**	MT	+	11,689	9	-	36,315,575	1	intergenic	-
**LUSC:87ac0ad4-8c9d-409e-9a86-1d201d01769d**	MT	+	15,936	9	+	33,864,273	0	UBE2R2(intron)	truncation
**LUSC:87ac0ad4-8c9d-409e-9a86-1d201d01769d**	MT	+	16,103	hs37d5	-	26,652,621	1	intergenic	-
**LUSC:87ac0ad4-8c9d-409e-9a86-1d201d01769d**	MT	+	6,285	15	-	76,219,463	1	FBXO22(intron)	truncation
**LUSC:9293e197-e38a-4e19-a7d0-1b45d1ad48bd**	MT	-	1,827	1	+	2,914,228	0	intergenic	-
**LUSC:9293e197-e38a-4e19-a7d0-1b45d1ad48bd**	MT	+	72	6	+	167,254,656	1	RPS6KA2(intron)	truncation
**LUSC:e58e8850-154f-4695-bee0-005c76410327**	MT	+	7,998	1	-	39,786,349	0	MACF1(intron)	truncation
**LUSC:e58e8850-154f-4695-bee0-005c76410327**	MT	+	8,139	1	+	39,786,037	1	MACF1(intron)	truncation
**MALY:4150895**	MT	+	11,170	4	+	137,895,166	0	intergenic	-
**MALY:4159421**	MT	+	5,248	2	-	145,091,202	0	intergenic	-
**OV:7ca97692-0bf5-4bbd-81ce-10a051d04bd5**	MT	+	12,488	11	-	72,613,495	1	FCHSD2(intron)	truncation
**OV:c435627c-159d-4a6d-a819-30abac24bf4d**	MT	+	16,113	16	+	60,469,789	0	intergenic	-
**PACA:ICGC_0543**	MT	+	16,083	4	-	157,262,727	0	intergenic	-
**PACA:ICGC_0543**	MT	+	32	5	+	131,561,359	1	P4HA2(intron)	not remarkable
**PBCA:MBRep_T27**	MT	+	16,114	9	+	95,506,702	0	BICD2(intron)	not remarkable
**PBCA:MBRep_T27**	MT	+	191	9	+	95,506,708	1	BICD2(intron)	not remarkable
**SARC:b85e2156-d5ac-486c-93e1-86525ea41a64**	MT	+	1,372	10	-	30,823,230	0	intergenic	-
**SKCM:01ad975d-c2ed-4e4d-bd3b-c9512fc9073c**	MT	+	11,568	6	+	3,751,700	0	PXDC1(exonic)	truncation
**SKCM:01ad975d-c2ed-4e4d-bd3b-c9512fc9073c**	MT	-	11,557	6	-	63,951,719	1	intergenic	-
**SKCM:81cedb9e-ee43-476b-9ed9-8d0fdb346065**	MT	+	5,081	8	+	90,465,476	0	intergenic	-
**SKCM:81cedb9e-ee43-476b-9ed9-8d0fdb346065**	MT	+	6,333	3	+	33,481,145	0	UBP1(intron)	truncation
**SKCM:81cedb9e-ee43-476b-9ed9-8d0fdb346065**	MT	+	15,032	20	+	62,710,708	1	RGS19(exonic)	truncation
**UCEC:24118b8f-59fe-482a-aed3-7b1bde1571b7**	MT	+	3,767	8	-	38,766,339	0	PLEKHA2	truncation
**UCEC:24118b8f-59fe-482a-aed3-7b1bde1571b7**	MT	+	4,287	8	-	36,603,622	1	intergenic	-
**UCEC:748e38b1-2ead-4a0a-8881-d640617b856b**	MT	+	16,186	22	-	48,182,869	0	AK093107(intron)	not remarkable
**UCEC:748e38b1-2ead-4a0a-8881-d640617b856b**	MT	+	16,274	22	-	48,181,913	0	AK093107(intron)	not remarkable
**UCEC:7b9947d5-e86b-4006-8890-ea5209584a88**	MT	+	8,033	9	+	12,816,690	1	intergenic	-

**UCEC:7b9947d5-e86b-4006-8890-ea5209584a88**	MT	+	13,424	9	-	12,841,745	1	intergenic	-
**BRCA:CGP_donor_1475202**	MT	+	16,323	1	-	197,335,986	1	intergenic	-
**BRCA:CGP_donor_1475202**	MT	+	approx. 1,693	hs37d5	+	approx. 4,907,000	0	intergenic	-
**BRCA:CGP_donor_1475202**	MT	+	approx. 1,770	hs37d5	-	approx. 28,645,400	1	intergenic	-
**ESAD:OCCAMS-AH-196**	MT	+	approx. 38	9	-	94,218,789	1	intergenic	-
**LIRI:RK004**	MT	+	7,673	5	+	93,303,764	1	FAM12A(intron)	indeterminable
**LIRI:RK082**	MT	+	14,850	11	+	125,083,145	0	PKNOX2(intron)	not remarkable
**LIRI:RK082**	MT	+	approx. 15,182	11	+	125,083,160	1	PKNOX2(intron)	not remarkable
**LIRI:RK243**	MT	+	1,315	12	+	64,095,441	0	intergenic	-
**LIRI:RK243**	MT	+	7,709	12	+	64,096,484	1	intergenic	-
**MELA:MELA-0001**	MT	+	14,906	9	-	44,776,871	1	intergenic	-
**MELA:MELA-0001**	MT	+	286	11	-	39,777,366	1	intergenic	-
**MELA:MELA-0001**	MT	+	16,105	16	-	50,038,448	0	intergenic	-
**MELA:MELA-0002**	MT	-	10,296	13	+	88,692,225	0	intergenic	-
**MELA:MELA-0002**	MT	+	13,807	13	-	88,709,481	1	intergenic	-
**MELA:MELA-0002**	MT	+	13,988	15	+	54,192,579	0	intergenic	-
**MELA:MELA-0002**	MT	+	6,933	15	+	54,168,518	1	intergenic	-
**MELA:MELA-0167**	MT	+	10,789	11	-	3,172,598	0	OSBPL5(intron)	truncation
**MELA:MELA-0167**	MT	+	2,936	15	+	28,354,949	1	intergenic	-
**MELA:MELA-0167**	MT	+	16,335	11	-	12,718,261	0	TEAD1(intron)	not remarkable
**MELA:MELA-0167**	MT	+	16,504	11	-	12,718,337	1	TEAD1(intron)	not remarkable
**MELA:MELA-0167**	MT	+	12,450	11	-	3,850,705	0	RHOG(intron)	indeterminable
**MELA:MELA-0167**	MT	+	41	17	+	48,554,863	1	intergenic	indeterminable
**MELA:MELA-0174**	MT	+	16,119	6	-	14,734,925	0	intergenic	-
**MELA:MELA-0174**	MT	+	151	3	+	33,188,139	1	CRTAP(3UTR)	not remarkable
**MELA:MELA-0174**	MT	+	3,921	15	-	31,338,568	0	TRPM1(intron)	not remarkable
**MELA:MELA-0174**	MT	+	6,767	15	-	31,338,588	1	TRPM1(intron)	not remarkable
**MELA:MELA-0203**	MT	+	14,549	6	+	98,286,935	0	intergenic	-
**MELA:MELA-0203**	MT	+	9,625	6	+	120,503,724	1	intergenic	-
**MELA:MELA-0213**	MT	+	16,197	9	+	20,945,617	1	FOCAD(intron)	indeterminable
**MELA:MELA-0259**	MT	+	3,380	8	-	141,859,251	0	PTK2(intron)	truncation
**MELA:MELA-0259**	MT	+	6,185	8	-	125,982,259	1	intergenic	-
**PACA:PCSI_0250**	MT	+	1,252	15	+	101,848,527	0	PCSK6(intron)	not remarkable
**PACA:PCSI_0250**	MT	+	?2400	15	+	101,848,646	1	PCSK6(intron)	not remarkable
**PRAD:CPCG0098**	MT	+	11,624	11	-	70,026,819	0	ANO1(intron)	truncation
**PRAD:CPCG0098**	MT	+	11,972	11	+	69,523,432	1	intergenic	-
**SARC:cd73a55f-03c1-4da0-9fa7-1ae4a263664e**	MT	+	4,939	12	+	112,788,982	0	HECTD4(intron)	truncation
**SARC:cd73a55f-03c1-4da0-9fa7-1ae4a263664e**	MT	+	4,478	12	-	112,793,558	1	HECTD4(intron)	truncation
**SKCM:c2948fe9-5798-43bc-a743-511e94e417f8**	MT	+	14,122	20	-	61,976,332	0	CHRNA4(3UTR)	not remarkable
**SKCM:c2948fe9-5798-43bc-a743-511e94e417f8**	MT	+	11,946	20	+	61,542,799	0	DIDO1(exonic)	truncation

Supplementary Table 4. An overview of the mtDNA RNA-sequencing (RNA-seq) data

Cancer (TCGA)	Description	Cancer tissue	Sample #	Average # mappable reads
**BLCA**	Bladder Cancer	Bladder	252	10.9M
**BRCA**	Breast Cancer	Breast	837	8.3M
**CESC**	Cervical Squamous Cell Carcinoma	Cervix	196	7.4M
**LGG**	Brain Lower Grade Glioma	CNS	486	13.3M
**HNSC**	Head and Neck Squamous Cell Carcinoma	Head/Neck	426	4.9M
**KICH**	Kidney Chromophobe	Kidney	66	43.3M
**KIRC**	Kidney Renal Clear Cell Carcinoma	Kidney	448	17.0M
**KIRP**	Kidney Renal Papillary Cell Carcinoma	Kidney	198	17.9M
**LIHC**	Liver Hepatocellular carcinoma	Liver	200	11.1M
**LUAD**	Lung Adenocarcinoma	Lung	488	6.1M
**LUSC**	Lung Squamous Cell Carcinoma	Lung	220	4.8M
**PRAD**	Prostate Adenocarcinoma	Prostate	374	8.8M
**THCA**	Head and Neck Thyroid Carcinoma	Thyroid	498	13.6M
**Total**	**13 cancer types**	**11 tissue types**	**4689**	**10.7M**

The ICGC/TCGA Pan-Cancer Analysis of Whole Genomes (PCAWG) Consortium Working Groups

PCAWG Steering committee

Peter J Campbell#[1,2], **Gad Getz#**[3,4,5,6], **Jan O Korbel#**[7,8], **Lincoln D Stein#**[9,10] and **Joshua M Stuart#**[11,12]

PCAWG Head of project management

Jennifer L Jennings[13]

PCAWG Executive committee

Sultan T Al-Sedairy[14], Axel Aretz[15], Cindy Bell[16], Miguel Betancourt[17], Christiane Buchholz[18], Fabien Calvo[19], Christine Chomienne[20], Michael Dunn[21], Stuart Edmonds[22], Eric Green[23], Shailja Gupta[24], Carolyn M Hutter[23], Karine Jegalian[25], Jennifer L Jennings[13], Nic Jones[26], Hyung-Lae Kim[27], Youyong Lu[28,29,30], Hitoshi Nakagama[31], Gerd Nettekoven[32], Laura Planko[32], David Scott[26], Tatsuhiro Shibata[33,34], Kiyo Shimizu[35], **Lincoln D Stein#**[9,10], Michael R Stratton[1], Takashi Yugawa[35], Giampaolo Tortora[36,37], K VijayRaghavan[24], Huanming Yang[38] and Jean C Zenklusen[39]

PCAWG Ethics and Legal Working Group

Don Chalmers#[40], Yann Joly[41], **Bartha M Knoppers#**[41], Fruzsina Molnár-Gábor[42], Mark Phillips[41], Adrian Thorogood[41] and David Townend[43]

PCAWG Technical Working Group

Brice Aminou[44], Javier Bartolome[45], Keith A Boroevich[46,47], Rich Boyce[7], Alvis Brazma[7], Angela N Brooks[3,11,12,48], Alex Buchanan[49], Ivo Buchhalter[50,51,52], Adam P Butler[1], Niall J Byrne[44], Andy Cafferkey[7], Peter J Campbell[1,2], Zhaohong Chen[53], Sunghoon Cho[54], Wan Choi[55], Peter Clapham[1], Brandi N Davis-Dusenbery[56], Francisco M De La Vega[57,58,59], Jonas Demeulemeester[60,61], Michelle T Dow[53], Lewis Jonathan Dursi[9,62], Juergen Eils[63,64], Roland Eils[50,52,63,64], Kyle Ellrott[49], Claudiu Farcas[53], Nodirjon Fayzullaev[44], Vincent Ferretti[44,65], Paul Flicek[7], Nuno A Fonseca[7,66], Josep L L Gelpi[45,67], Gad Getz[3,4,5,6], Robert L Grossman[68], Olivier Harismendy[69,70], Allison P Heath[71], Michael C Heinold[50,52], Julian M Hess[3,72], Oliver Hofmann[73], Jongwhi H Hong[74], Thomas J Hudson[75,76], Barbara Hutter[77,78,79], Carolyn M Hutter[23], Daniel Hübschmann[52,63,80,81,82], Seiya Imoto[83], Sinisa Ivkovic[56], Seung-Hyup Jeon[55], Wei Jiao[9], Jongsun Jung[84], Rolf Kabbe[50], Andre Kahles[85,86,87,88,89], Jules NA Kerssemakers[50], Hyung-Lae Kim[27], Hyunghwan Kim[55], Jihoon Kim[90], Youngwook Kim[91,92],

Kortine Kleinheinz[50,52], Jan O Korbel[7,8], Michael Koscher[93], Antonios Koures[53], Milena Kovacevic[56], Chris Lawerenz[64], Ignaty Leshchiner[3], Jia Liu[94], Dimitri Livitz[3], George L Mihaiescu[44], Sanja Mijalkovic[56], Ana Mijalkovic Mijalkovic-Lazic[56], Satoru Miyano[83], Naoki Miyoshi[83], Hardeep K Nahal-Bose[44], Hidewaki Nakagawa[47], Mia Nastic[56], Steven J Newhouse[7], Jonathan Nicholson[1], **Brian D O'Connor#**[44,95], David Ocana[7], Kazuhiro Ohi[83], Lucila Ohno-Machado[53], Larsson Omberg[96], BF Francis Ouellette[44,97], Nagarajan Paramasivam[50,78], Marc D Perry[44,98], Todd D Pihl[99], Manuel Prinz[50], Montserrat Puiggròs[45], Petar Radovic[56], Keiran M Raine[1], Esther Rheinbay[3,6,100], Mara Rosenberg[3,100], Romina Royo[45], Gunnar Rätsch[85,86,87,88,89,101], Gordon Saksena[3], Matthias Schlesner[50,102], Solomon I Shorser[9], Charles Short[7], Heidi J Sofia[23], Jonathan Spring[103], **Lincoln D Stein#**[9,10], Adam J Struck[104], Grace Tiao[3], Nebojsa Tijanic[56], David Torrents[45,105], Peter Van Loo[60,61], Miguel Vazquez[45,106], David Vicente[45], Jeremiah A Wala[3,6,48], Zhining Wang[39], Sebastian M Waszak[8], Joachim Weischenfeldt[8,107,108], Johannes Werner[50,109], Ashley Williams[53], Youngchoon Woo[55], Adam J Wright[9], Qian Xiang[44], **Sergei Yakneen#**[8], Liming Yang[39], Denis Yuen[9], **Christina K Yung#**[44] and **Junjun Zhang#**[44]

PCAWG Reference Annotations Working Group

Angela N Brooks[3,11,12,48], Ivo Buchhalter[50,51,52], Peter J Campbell[1,2], Priyanka Dhingra[110,111], Lars Feuerbach[112], Mark Gerstein[113,114,115], Gad Getz[3,4,5,6], Mark P Hamilton[116], Henrik Hornshøj[117], Todd A Johnson[46], Andre Kahles[85,86,87,88,89], Abdullah Kahraman[118,119,120], Manolis Kellis[3,121], **Ekta Khurana#**[110,111,122,123], Jan O Korbel[7,8], Morten Muhlig Nielsen[117], Jakob Skou Pedersen[117,124], Paz Polak[3,4,6], Jüri Reimand[9,125], Esther Rheinbay[3,6,100], Nicola D Roberts[1], Gunnar Rätsch[85,86,87,88,89,101], Richard Sallari[3], Nasa Sinnott-Armstrong[3,59], Alfonso Valencia[45,105], Miguel Vazquez[45,106], Sebastian M Waszak[8], Joachim Weischenfeldt[8,107,108] and Christian von Mering[120,126]

PCAWG Quality-Control Working Group

Sergi Beltran[127,128], Ivo Buchhalter[50,51,52], Peter J Campbell[1,2], Roland Eils[50,52,63,64], Daniela S Gerhard[129], Gad Getz[3,4,5,6], **Ivo G Gut#**[127,128], Marta Gut[127,128], Barbara Hutter[77,78,79], Daniel Hübschmann[52,63,80,81,82], Kortine Kleinheinz[50,52], Jan O Korbel[7,8], Dimitri Livitz[3], Marc D Perry[44,98], Keiran M Raine[1], Esther Rheinbay[3,6,100], Mara Rosenberg[3,100], Gordon Saksena[3], Matthias Schlesner[50,102], Miranda D Stobbe[127,128], Jean-Rémi Trotta[127], Johannes Werner[50,109] and Justin P Whalley[127]

PCAWG SNV Calling Working Group

Matthew H Bailey[130,131], Beifang Niu[132], Matthias Bieg[78,133], Paul C Boutros[9,125,134,135], Ivo Buchhalter[50,51,52], Adam P Butler[1], Ken Chen[136], Zechen Chong[137], **Li Ding#**[130,131,138], Oliver Drechsel[128,139], Lewis Jonathan Dursi[9,62], Roland Eils[50,52,63,64], Kyle Ellrott[49], Shadrielle MG Espiritu[9], Yu Fan[140], Robert S Fulton[130,131,138], Shengjie Gao[38], Josep L L Gelpi[45,67], Mark Gerstein[113,114,115], Gad Getz[3,4,5,6], Santiago Gonzalez[7,8], Ivo G Gut[127,128], Faraz Hach[141,142], Michael C Heinold[50,52], Julian M Hess[3,72], Jonathan Hinton[1], Taobo Hu[143], Vincent Huang[9], Yi Huang[144,145], Barbara Hutter[77,78,79],

David R Jones[1], Jongsun Jung[84], Natalie Jäger[50], Hyung-Lae Kim[27], Kortine Kleinheinz[50,52], Sushant Kumar[114,115], Yogesh Kumar[143], Christopher M Lalansingh[9], Ignaty Leshchiner[3], Ivica Letunic[146], Dimitri Livitz[3], Eric Z Ma[143], Yosef E Maruvka[3,72,100], R Jay Mashl[131,147], Michael D McLellan[130,131,138], Andrew Menzies[1], Ana Milovanovic[45], Morten Muhlig Nielsen[117], Stephan Ossowski[128,139,148], Nagarajan Paramasivam[50,78], Jakob Skou Pedersen[117,124], Marc D Perry[44,98], Montserrat Puiggròs[45], Keiran M Raine[1], Esther Rheinbay[3,6,100], Romina Royo[45], S Cenk Sahinalp[142,149,150], Gordon Saksena[3], Iman Sarrafi[142,150], Matthias Schlesner[50,102], **Jared T Simpson#**[9,151], Lucy Stebbings[1], Chip Stewart[3], Miranda D Stobbe[127,128], Jon W Teague[1], Grace Tiao[3], David Torrents[45,105], Jeremiah A Wala[3,6,48], Jiayin Wang[131,145,152], Wenyi Wang[140], Sebastian M Waszak[8], Joachim Weischenfeldt[8,107,108], Michael C Wendl[131,138,153], Johannes Werner[50,109], David A Wheeler[154,155], Zhenggang Wu[143], Hong Xue[143], Sergei Yakneen[8], Takafumi N Yamaguchi[9], Kai Ye[152,156], Venkata D Yellapantula[138,157], Christina K Yung[44] and Junjun Zhang[44]

PCAWG Drivers and Functional Interpretation Working Group

Federico Abascal[1], Samirkumar B Amin[158,159,160], Gary D Bader[10], Pratiti Bandopadhayay[3,161,162], Jonathan Barenboim[9], Rameen Beroukhim[3,6,163], Johanna Bertl[117,164], Keith A Boroevich[46,47], Søren Brunak[165,166], Peter J Campbell[1,2], Joana Carlevaro-Fita[167,168,169], Dimple Chakravarty[170], Calvin Wing Yiu Chan[50,171], Ken Chen[136], Jung Kyoon Choi[172], Jordi Deu-Pons[173,174], Priyanka Dhingra[110,111], Klev Diamanti[175], Lars Feuerbach[112], J Lynn Fink[45,176], Nuno A Fonseca[7,66], Joan Frigola[173], Carlo Gambacorti-Passerini[177], Dale W Garsed[178], **Mark Gerstein#**[113,114,115], **Gad Getz#**[3,4,5,6], Qianyun Guo[124], Ivo G Gut[127,128], David Haan[11], Mark P Hamilton[116], Nicholas J Haradhvala[3,100], Arif O Harmanci[115,179], Mohamed Helmy[180], Carl Herrmann[50,52,181], Julian M Hess[3,72], Asger Hobolth[124,164], Ermin Hodzic[150], Chen Hong[112,171], Henrik Hornshøj[117], Keren Isaev[9,125], Jose MG Izarzugaza[182], Rory Johnson[168,183], Todd A Johnson[46], Malene Juul[117], Randi Istrup Juul[117], Andre Kahles[85,86,87,88,89], Abdullah Kahraman[118,119,120], Manolis Kellis[3,121], Ekta Khurana[110,111,122,123], Jaegil Kim[3], Jong K Kim[184], Youngwook Kim[91,92], Jan Komorowski[175,185], Jan O Korbel[7,8], Sushant Kumar[114,115], Andrés Lanzós[168,169,183], Erik Larsson[85], **Michael S Lawrence#**[3,46,100], Donghoon Lee[115], Kjong-Van Lehmann[85,86,87,88,89], Shantao Li[115], Xiaotong Li[115], Ziao Lin[3,186], Eric Minwei Liu[110,111,187], Lucas Lochovsky[114,115,160], Shaoke Lou[114,115], Tobias Madsen[117], Kathleen Marchal[188,189], Iñigo Martincorena[1], Alexander Martinez-Fundichely[110,111,123], Yosef E Maruvka[3,72,100], Patrick D McGillivray[114], William Meyerson[115,190], Ferran Muiños[174,191], Loris Mularoni[174,191], Hidewaki Nakagawa[47], Morten Muhlig Nielsen[117], Marta Paczkowska[9], Keunchil Park[192,193], Kiejung Park[194], **Jakob Skou Pedersen#**[117,124], Tirso Pons[195], Sergio Pulido-Tamayo[188,189], **Benjamin J Raphael#**[196], Jüri Reimand[9,125], Iker Reyes-Salazar[191], Matthew A Reyna[196], Esther Rheinbay[3,6,100], Mark A Rubin[183,197,198,199,200], Carlota Rubio-Perez[174,191,201], S Cenk Sahinalp[142,149,150], Gordon Saksena[3], Leonidas Salichos[114,115], Chris Sander[85,202,203], Steven E Schumacher[3,204], Mark Shackleton[178], Ofer Shapira[3,205], Ciyue Shen[203,206], Raunak Shrestha[142], Shimin Shuai[9,10], Nikos Sidiropoulos[108], Lina Sieverling[112,171], Nasa Sinnott-Armstrong[3,59], Lincoln D Stein[9,10], **Joshua M Stuart#**[11,12], David Tamborero[174,191], Grace Tiao[3], Tatsuhiko Tsunoda[46,207,208,209], Husen M Umer[175,210], Liis Uusküla-Reimand[211,212], Alfonso Valencia[45,105], Miguel Vazquez[45,106], Lieven PC Verbeke[189,213], Claes Wadelius[214], Lina Wadi[9], Jiayin Wang[131,145,152], Jonathan Warrell[114,115], Sebastian M Waszak[8], Joachim Weischenfeldt[8,107,108], **David A Wheeler#**[154,155], Guanming Wu[215], Jun Yu[216], Jing Zhang[115], Xuanping Zhang[145,217], Yan Zhang[115,218,219], Zhongming Zhao[220], Lihua Zou[221] and Christian von

Mering[120,126]

PCAWG Transcriptome Working Group

Samirkumar B Amin[158,159,160], Philip Awadalla[9,10], Peter J Bailey[222], **Alvis Brazma#**[7], **Angela N Brooks#**[3,11,12,48], Claudia Calabrese[7,8], Aurélien Chateigner[44], Isidro Cortés-Ciriano[223,224,225], Brian Craft[12], David Craft[3,226], Chad J Creighton[227], Natalie R Davidson[85,86,87,88,101], Deniz Demircioğlu[228,229], Serap Erkek[8], Nuno A Fonseca[7,66], Milana Frenkel-Morgenstern[230], Mary J Goldman[12], Liliana Greger[7], Jonathan Göke[228,231], Yao He[232], Katherine A Hoadley[233,234], Yong Hou[38,235], Matthew R Huska[236], Andre Kahles[85,86,87,88,89], Ekta Khurana[110,111,122,123], Helena Kilpinen[237], Jan O Korbel[7,8], Fabien C Lamaze[9], Kjong-Van Lehmann[85,86,87,88,89], Chang Li[38,235], Siliang Li[38,235], Xiaobo Li[38,235], Xinyue Li[38], Dongbing Liu[38,235], Fenglin Liu[232,238], Xingmin Liu[38,235], Maximillian G Marin[11], Julia Markowski[236], Matthew Meyerson[3,6,48], Tannistha Nandi[239], Morten Muhlig Nielsen[117], Akinyemi I Ojesina[240,241,242], BF Francis Ouellette[44,97], Qiang Pan-Hammarström[38,243], Peter J Park[223,225], Chandra Sekhar Pedamallu[3,6,163], Jakob Skou Pedersen[117,124], Marc D Perry[44,98], **Gunnar Rätsch#**[85,86,87,88,89,101], Roland F Schwarz[7,81,236,244], Yuichi Shiraishi[83], Reiner Siebert[245,246], Cameron M Soulette[11], Stefan G Stark[86,88,247,248], Oliver Stegle[7,8,249], Hong Su[38,235], Patrick Tan[239,250,251,252], Bin Tean Teh[250,251,252,253,254], Lara Urban[7,8], Jian Wang[38], Sebastian M Waszak[8], Kui Wu[38,235], Qian Xiang[44], Heng Xiong[38,235], Sergei Yakneen[8], Huanming Yang[38], Chen Ye[38,235], Christina K Yung[44], Fan Zhang[232], Junjun Zhang[44], Xiuqing Zhang[38], Zemin Zhang[232,255], Liangtao Zheng[232], Jingchun Zhu[12] and Shida Zhu[38,235]

PCAWG Epigenome Working Group

Hiroyuki Aburatani[256], **Benjamin P Berman#**[257,258,259], Hans Binder[260,261], **Benedikt Brors#**[79,112,262], Huy Q Dinh[257], Lars Feuerbach[112], Shengjie Gao[38], Ivo G Gut[127,128], Simon C Heath[127,128], Steve Hoffmann[260,261,263,264], Charles David Imbusch[112], Ekta Khurana[110,111,122,123], Helene Kretzmer[261,264], Peter W Laird[265], Jose I Martin-Subero[105,266], Genta Nagae[256,267], **Christoph Plass#**[268], Paz Polak[3,4,6], Hui Shen[269], Reiner Siebert[245,246], Nasa Sinnott-Armstrong[3,59], Miranda D Stobbe[127,128], Qi Wang[93], Dieter Weichenhan[268], Sergei Yakneen[8] and Wanding Zhou[269]

PCAWG Structural Variation Working Group

Kadir C Akdemir[136], Eva G Alvarez[270,271,272], Adrian Baez-Ortega[273], **Rameen Beroukhim#**[3,6,163], Paul C Boutros[9,125,134,135], David D L Bowtell[178], Benedikt Brors[79,112,262], Kathleen H Burns[274,275], John Busanovich[3,276], **Peter J Campbell#**[1,2], Kin Chan[277], Ken Chen[136], Isidro Cortés-Ciriano[223,224,225], Ana Dueso-Barroso[45], Andrew J Dunford[3], Paul A Edwards[278,279], Xavier Estivill[139,280], Dariush Etemadmoghadam[178], Lars Feuerbach[112], J Lynn Fink[45,176], Milana Frenkel-Morgenstern[230], Dale W Garsed[178], Mark Gerstein[113,114,115], Dmitry A Gordenin[281], David Haan[11], James E Haber[282], Julian M Hess[3,72], Barbara Hutter[77,78,79], Marcin Imielinski[283,284], David TW Jones[285,286], Young Seok Ju[1,172], Marat D Kazanov[287,288,289], Leszek J Klimczak[290], Youngil Koh[291,292], Jan O Korbel[7,8], Kiran Kumar[3], Eunjung Alice Lee[293], Jake June-Koo Lee[223,225], Yilong Li[1], Andy G Lynch[278,279,294], Geoff

Macintyre[278], Florian Markowetz[278,279], Iñigo Martincorena[1], Alexander Martinez-Fundichely[110,111,123], Satoru Miyano[83], Hidewaki Nakagawa[47], Fabio CP Navarro[114], Stephan Ossowski[128,139,148], Peter J Park[223,225], John V Pearson[295,296], Montserrat Puiggròs[45], Karsten Rippe[81], Nicola D Roberts[1], Steven A Roberts[297], Bernardo Rodriguez-Martin[270,271,272], Steven E Schumacher[3,204], Ralph Scully[298], Mark Shackleton[178], Nikos Sidiropoulos[108], Lina Sieverling[112,171], Chip Stewart[3], David Torrents[45,105], Jose MC Tubio[270,271,272], Izar Villasante[45], Nicola Waddell[295,296], Jeremiah A Wala[3,6,48], Joachim Weischenfeldt[8,107,108], Lixing Yang[299], Xiaotong Yao[284,300], Sung-Soo Yoon[292], Jorge Zamora[1,270,271,272] and Cheng-Zhong Zhang[3,301]

PCAWG Mutational Signatures Working Group

Ludmil B Alexandrov[1,70,302], Erik N Bergstrom[70,303], Arnoud Boot[251,304], Paul C Boutros[9,125,134,135], Kin Chan[277], Kyle Covington[155], Akihiro Fujimoto[47], Gad Getz[3,4,5,6], Dmitry A Gordenin[281], Nicholas J Haradhvala[3,100], Mi Ni Huang[251,304], S. M. Ashiqul Islam[53], Marat D Kazanov[287,288,289], Jaegil Kim[3], Leszek J Klimczak[290], Michael S Lawrence[3,46,100], Iñigo Martincorena[1], John R McPherson[251,304], Sandro Morganella[1], Ville Mustonen[305,306,307], Hidewaki Nakagawa[47], Alvin Wei Tian Ng[308], Serena Nik-Zainal[1,309,310,311], Paz Polak[3,4,6], Stephenie D Prokopec[9], Steven A Roberts[297], **Steven G Rozen#**[251,252,304], Radhakrishnan Sabarinathan[174,191,312], Natalie Saini[281], Tatsuhiro Shibata[33,34], Yuichi Shiraishi[83], **Michael R Stratton#**[1], **Bin Tean Teh#**[250,251,252,253,254], Ignacio Vázquez-García[1,157,313,314], Yang Wu[251,304], Fouad Yousif[9] and Willie Yu[315]

PCAWG Germline Cancer Genome Working Group

Ludmil B Alexandrov[1,70,302], Eva G Alvarez[270,271,272], Adrian Baez-Ortega[273], Matthew H Bailey[130,131], Mattia Bosio[45,128,139], G Steven Bova[316], Alvis Brazma[7], Alicia L Bruzos[270,271,272], Ivo Buchhalter[50,51,52], Carlos D Bustamante[58,59], Atul J Butte[317], Andy Cafferkey[7], Claudia Calabrese[7,8], Peter J Campbell[1,2], Stephen J Chanock[318], Nilanjan Chatterjee[319,320], Jieming Chen[115,321], Francisco M De La Vega[57,58,59], Olivier Delaneau[322,323,324], German M Demidov[128,139,148], Anthony DiBiase[325], Li Ding[130,131,138], Oliver Drechsel[128,139], Lewis Jonathan Dursi[9,62], Douglas F Easton[326,327], Serap Erkek[8], Georgia Escaramis[139,328,329], **Xavier Estivill#**[139,280], Erik Garrison[1], Mark Gerstein[113,114,115], Gad Getz[3,4,5,6], Dmitry A Gordenin[281], Nina Habermann[8], Olivier Harismendy[69,70], Eoghan Harrington[330], Shuto Hayashi[83], Seong Gu Heo[331], José María Heredia-Genestar[332], Aliaksei Z Holik[139], Eun Pyo Hong[331], Xing Hua[318], Kuan-lin Huang[131,333], Seiya Imoto[83], Sissel Juul[330], Ekta Khurana[110,111,122,123], Hyung-Lae Kim[27], Youngwook Kim[91,92], Leszek J Klimczak[290], **Jan O Korbel#**[7,8], Roelof Koster[334], Sushant Kumar[114,115], Ivica Letunic[146], Yilong Li[1], Tomas Marques-Bonet[105,127,332,335], R Jay Mashl[131,147], Simon Mayes[336], Michael D McLellan[130,131,138], Lisa Mirabello[318], Francesc Muyas[128,139,148], Hidewaki Nakagawa[47], Arcadi Navarro[105,127,332], Steven J Newhouse[7], Stephan Ossowski[128,139,148], Ji Wan Park[331], Esa Pitkänen[8], Aparna Prasad[128], Raquel Rabionet[128,139,337], Benjamin Raeder[8], Tobias Rausch[8], Steven A Roberts[297], Bernardo Rodriguez-Martin[270,271,272], Vasilisa A Rudneva[8], Gunnar Rätsch[85,86,87,88,89,101], Natalie Saini[281], Matthias Schlesner[50,102], Roland F Schwarz[7,81,236,244], Ayellet V Segre[3,338], Tal Shmaya[57], Suyash S Shringarpure[59], Nikos Sidiropoulos[108], Reiner Siebert[245,246], Jared T Simpson[9,151], Lei Song[318], Oliver Stegle[7,8,249], Hana Susak[128,139], Tomas J Tanskanen[339], Grace Tiao[3], Marta Tojo[272], Jose MC

Tubio[270,271,272], Daniel J Turner[336], Lara Urban[7,8], Sebastian M Waszak[8], David C Wedge[1,340,341], Joachim Weischenfeldt[8,107,108], David A Wheeler[154,155], Mark H Wright[59], Dai-Ying Wu[57], Tian Xia[342], Sergei Yakneen[8], Kai Ye[152,156], Venkata D Yellapantula[138,157], Jorge Zamora[1,270,271,272] and Bin Zhu[318]

PCAWG Pathology and Clinical Correlates Working Group

Fatima Al-Shahrour[343], Gurnit Atwal[9,10,344], Peter J Bailey[222], **Andrew V Biankin#**[222,345,346,347], Paul C Boutros[9,125,134,135], Peter J Campbell[1,2], David K Chang[222,345], Susanna L Cooke[222], Vikram Deshpande[100], Bishoy M Faltas[101], William C Faquin[100], **Levi Garraway#**[48], Gad Getz[3,4,5,6], **Sean M Grimmond#**[348], Syed Haider[9], **Katherine A Hoadley#**[233,234], Wei Jiao[9], Vera B Kaiser[349], Rosa Karlić[350], Mamoru Kato[351], Kirsten Kübler[3,6,100], Alexander J Lazar[158,352], Constance H Li[9,125], David N Louis[100], Adam A Margolin[104], Sancha Martin[1,353], Hardeep K Nahal-Bose[44], G Petur Nielsen[100], Serena Nik-Zainal[1,309,310,311], Larsson Omberg[96], Christine P'ng[9], Marc D Perry[44,98], Paz Polak[3,4,6], Esther Rheinbay[3,6,100], Mark A Rubin[183,197,198,199,200], Colin A Semple[349], Dennis C Sgroi[100], Tatsuhiro Shibata[33,34], Reiner Siebert[245,246], Jaclyn Smith[354], **Lincoln D Stein#**[9,10], Miranda D Stobbe[127,128], Ren X Sun[9], Kevin Thai[44], Derek W Wright[222,355], Chin-Lee Wu[100], Ke Yuan[278,353,356] and Junjun Zhang[44]

PCAWG Evolution & Heterogeneity Working Group

David J Adams[1], Pavana Anur[357], Rameen Beroukhim[3,6,163], Paul C Boutros[9,125,134,135], David D L Bowtell[178], Peter J Campbell[1,2], Shaolong Cao[140], Elizabeth L Christie[178], Marek Cmero[358,359,360], Yupeng Cun[361], Kevin J Dawson[1], Jonas Demeulemeester[60,61], Stefan C Dentro[1,60,340], Amit G Deshwar[362], Nilgun Donmez[142,150], Ruben M Drews[278], Roland Eils[50,52,63,64], Yu Fan[140], Matthew W Fittall[60], Dale W Garsed[178], Moritz Gerstung[7,8], Gad Getz[3,4,5,6], Santiago Gonzalez[7,8], Gavin Ha[3], Kerstin Haase[60], Marcin Imielinski[283,284], Lara Jerman[8,363], Yuan Ji[364,365], Clemency Jolly[60], Kortine Kleinheinz[50,52], Juhee Lee[366], Henry Lee-Six[1], Ignaty Leshchiner[3], Dimitri Livitz[3], Geoff Macintyre[278], Salem Malikic[142,150], Florian Markowetz[278,279], Iñigo Martincorena[1], Thomas J Mitchell[1,279,367], Quaid D Morris[344,368], Ville Mustonen[305,306,307], Layla Oesper[369], Martin Peifer[361], Myron Peto[357], Benjamin J Raphael[196], Daniel Rosebrock[3], Yulia Rubanova[151,344], S Cenk Sahinalp[142,149,150], Adriana Salcedo[9], Matthias Schlesner[50,102], Steven E Schumacher[3,204], Subhajit Sengupta[370], Ruian Shi[368], Seung Jun Shin[248], **Paul T Spellman#**[357], Oliver Spiro[3], Lincoln D Stein[9,10], Maxime Tarabichi[1,60], **Peter Van Loo#**[60,61], Shankar Vembu[368,371], Ignacio Vázquez-García[1,157,313,314], Wenyi Wang[140], **David C Wedge#**[1,340,341], David A Wheeler[154,155], Jeffrey A Wintersinger[151,180,344], Tsun-Po Yang[361], Xiaotong Yao[284,300], Kaixian Yu[372], Ke Yuan[278,353,356] and Hongtu Zhu[372,373]

PCAWG Portals and Visualization Working Group

Fatima Al-Shahrour[343], Elisabet Barrera[7], Wojciech Bazant[7], Alvis Brazma[7], Isidro Cortés-Ciriano[223,224,225], Brian Craft[12], David Craft[3,226], Vincent Ferretti[44,65], Nuno A Fonseca[7,66],

Anja Füllgrabe[7], Mary J Goldman[12], **David Haussler#**[12,374], Wolfgang Huber[8], Maria Keays[7], Alfonso Muñoz[7], Brian D O'Connor[44,95], Irene Papatheodorou[7], Robert Petryszak[7], Elena Piñeiro-Yáñez[343], Alfonso Valencia[45,105], **Miguel Vazquez#**[45,106], John N Weinstein[375,376], Qian Xiang[44], Junjun Zhang[44] and **Jingchun Zhu#**[12]

PCAWG Mitochondrial Genome and Immunogenomics Working Group

Peter J Campbell[1,2], Yiwen Chen[140], Chad J Creighton[227], Li Ding[130,131,138], Akihiro Fujimoto[47], Masashi Fujita[47], Gad Getz[3,4,5,6], Leng Han[217], Takanori Hasegawa[83], Shuto Hayashi[83], Seiya Imoto[83], Young Seok Ju[1,172], Hyung-Lae Kim[27], Youngwook Kim[91,92], Youngil Koh[291,292], Mitsuhiro Komura[83], Jun Li[140], **Han Liang#**[140], Iñigo Martincorena[1], Satoru Miyano[83], Shinichi Mizuno[377], **Hidewaki Nakagawa#**[47], Keunchil Park[192,193], Eigo Shimizu[83], Yumeng Wang[140], John N Weinstein[375,376], Yanxun Xu[378], Rui Yamaguchi[83], Fan Yang[368], Yang Yang[217], Christopher J Yoon[172], Sung-Soo Yoon[292], Yuan Yuan[140], Fan Zhang[232] and Zemin Zhang[232,255]

PCAWG Pathogens Working Group

Malik Alawi[379,380], Ivan Borozan[9], Daniel S Brewer[381,382], Colin S Cooper[382,383,384], Nikita Desai[44], Roland Eils[50,52,63,64], Vincent Ferretti[44,65], Adam Grundhoff[380,385], Murat Iskar[386], Kortine Kleinheinz[50,52], **Peter Lichter#**[77,386], Hidewaki Nakagawa[47], Akinyemi I Ojesina[240,241,242], Chandra Sekhar Pedamallu[3,6,163], Matthias Schlesner[50,102], Xiaoping Su[352] and **Marc Zapatka#**[386]

Providers of tumour-sequencing data

PCAWG Tumour-specific providers (ovarian cancer) in Australia

Kathryn Alsop[178], Australian Ovarian Cancer Study Group[295,387,388], **David D L Bowtell#**[178], Timothy JC Bruxner[176], Angelika N Christ[176], Elizabeth L Christie[178], Stephen M Cordner[389], Prue A Cowin[387], Ronny Drapkin[390], Dariush Etemadmoghadam[178], Sian Fereday[178], Dale W Garsed[178], Joshy George[160], Sean M Grimmond[348], Anne Hamilton[387], Oliver Holmes[295,296], Jillian A Hung[391], Karin S Kassahn[176,392], Stephen H Kazakoff[295,296], Catherine J Kennedy[391,393], Conrad R Leonard[295,296], Linda Mileshkin[178], David K Miller[176,345], Gisela Mir Arnau[387], Chris Mitchell[178], Felicity Newell[295,296], Katia Nones[295,296], Ann-Marie Patch[295,296], John V Pearson[295,296], Michael C Quinn[295,296], Mark Shackleton[178], Darrin F Taylor[176], Heather Thorne[178], Nadia Traficante[178], Ravikiran Vedururu[387], Nick M Waddell[296], Nicola Waddell[295,296], Paul M Waring[394], Scott Wood[295,296], Qinying Xu[295,296] and Anna deFazio[391,393,395]

PCAWG Tumour-specific providers (pancreatic cancer) in Australia

Matthew J Anderson[176], Davide Antonello[396], Andrew P Barbour[397,398], Claudio Bassi[396], Samantha Bersani[399], **Andrew V Biankin#**[222,345,346,347], Timothy JC Bruxner[176], Ivana Cataldo[399,400], David K Chang[222,345], Lorraine A Chantrill[345,401], Yoke-Eng Chiew[391,393], Angela Chou[345,393], Angelika N Christ[176], Sara Cingarlini[36], Nicole Cloonan[402], Vincenzo Corbo[400,403], Maria Vittoria Davi[404], Fraser R Duthie[222,405], J Lynn Fink[45,176], Anthony J Gill[345,406], Janet S Graham[222,407], **Sean M Grimmond#**[348], Ivon Harliwong[176], Oliver Holmes[295,296], Nigel B Jamieson[222,347,408], Amber L Johns[345], Karin S Kassahn[176,392], Stephen H Kazakoff[295,296], James G Kench[345,406,409], Luca Landoni[396], Rita T Lawlor[400], Conrad R Leonard[295,296], Andrea Mafficini[400], Neil D Merrett[396,410], David K Miller[176,345], Marco Miotto[396], Elizabeth A Musgrove[222], Adnan M Nagrial[345], Felicity Newell[295,296], Katia Nones[295,296], Karin A Oien[394,411], Marina Pajic[345], Ann-Marie Patch[295,296], John V Pearson[295,296], Mark Pinese[345], Andreia V Pinho[412], Michael C Quinn[295,296], Alan J Robertson[176], Ilse Rooman[345], Borislav C Rusev[400], Jaswinder S Samra[396,413], Maria Scardoni[399], Christopher J Scarlett[345,414], Aldo Scarpa[400], Elisabetta Sereni[396], Katarzyna O Sikora[400], Michele Simbolo[403], Morgan L Taschuk[44], Christopher W Toon[345], Giampaolo Tortora[36,37], Caterina Vicentini[400], Nick M Waddell[296], Nicola Waddell[295,296], Scott Wood[295,296], Jianmin Wu[345], Qinying Xu[295,296] and Nikolajs Zeps[415,416]

PCAWG Tumour-specific providers (skin cancer) in Australia

Lauri A Aaltonen[417], Andreas Behren[418], Hazel Burke[419], Jonathan Cebon[418], Rebecca A Dagg[420], Ricardo De Paoli-Iseppi[419], Ken Dutton-Regester[295], Matthew A Field[421], Anna Fitzgerald[422], Sean M Grimmond[348], **Nicholas K Hayward#**[295,419], Peter Hersey[419], Oliver Holmes[295,296], Valerie Jakrot[419], Peter A Johansson[295], Hojabr Kakavand[419], Stephen H Kazakoff[295,296], Richard F Kefford[423], Loretta MS Lau[424], Conrad R Leonard[295,296], Georgina V Long[419], **Graham J Mann#**[393,419,419], Felicity Newell[295,296], Katia Nones[295,296], Ann-Marie Patch[295,296], John V Pearson[295,296], Hilda A Pickett[424], Antonia L Pritchard[295], Gulietta M Pupo[393], Robyn PM Saw[419], Sarah-Jane Schramm[393], **Richard A Scolyer#**[409,413,419], Mark Shackleton[178], Catherine A Shang[422], Ping Shang[419], Andrew J Spillane[419], Jonathan R Stretch[419], Varsha Tembe[393], John F Thompson[419], Ricardo E Vilain[425], Nick M Waddell[296], Nicola Waddell[295,296], James S Wilmott[419], Scott Wood[295,296], Qinying Xu[295,296] and Jean Y Yang[426]

PCAWG Tumour-specific providers (pancreatic cancer) in Canada

John Bartlett[427,428], Prashant Bavi[429], Ivan Borozan[9], Dianne E Chadwick[430], Michelle Chan-Seng-Yue[429], Sean Cleary[429,431], Ashton A Connor[431,432], Karolina Czajka[76], Robert E Denroche[429], Neesha C Dhani[433], Jenna Eagles[76], Vincent Ferretti[44,65], Steven Gallinger[429,431,432], Robert C Grant[429,432], David Hedley[433], Michael A Hollingsworth[434], **Thomas J Hudson#**[75,76], Gun Ho Jang[429], Jeremy Johns[76], Sangeetha Kalimuthu[429], Sheng-Ben Liang[430], Ilinca Lungu[429,435], Xuemei Luo[9], Faridah Mbabaali[76], **John D McPherson#**[76,429,436], Treasa A McPherson[432], Jessica K Miller[76], Malcolm J Moore[433], Faiyaz Notta[429,437], Danielle Pasternack[76], Gloria M Petersen[438], Michael H A Roehrl[125,429,430,439,440,441], Michelle Sam[76], Iris Selander[432], Stefano Serra[394], Sagedeh Shahabi[430], **Lincoln D Stein#**[9,10], Morgan L Taschuk[44], Sarah P Thayer[434], Lee E Timms[76], Gavin W Wilson[9,429], Julie M Wilson[429] and Bradly G Wouters[125]

PCAWG Tumour-specific providers (prostate cancer) in Canada

Timothy A Beck[44,442], Vinayak Bhandari[9], **Paul C Boutros#**[9,125,134,135], **Robert G Bristow#**[125,443,444,445,446], Colin C Collins[142], Shadrielle MG Espiritu[9], Neil E Fleshner[447], Natalie S Fox[9], Michael Fraser[9], Syed Haider[9], Lawrence E Heisler[44], Vincent Huang[9], Emilie Lalonde[9], Julie Livingstone[9], John D McPherson[76,429,436], Alice Meng[448], Veronica Y Sabelnykova[9], Adriana Salcedo[9], Yu-Jia Shiah[9], Theodorus Van der Kwast[441] and Takafumi N Yamaguchi[9]

PCAWG Tumour-specific providers (gastric cancer) in China

Shuai Ding[449], Daiming Fan[450], Yong Hou[38,235], Yi Huang[144,145], Lin Li[38], Siliang Li[38,235], Dongbing Liu[38,235], Xingmin Liu[38,235], **Youyong Lu#**[28,29,30], Yongzhan Nie[450,451], Hong Su[38,235], Jian Wang[38], Kui Wu[38,235], Xiao Xiao[145], Rui Xing[30], **Huanming Yang#**[38], Shanlin Yang[449], Yingyan Yu[452], Xiuqing Zhang[38], Yong Zhou[38] and Shida Zhu[38,235]

PCAWG Tumour-specific providers (renal cancer) in the EU & France

Rosamonde E Banks[453], Guillaume Bourque[454,455], Alvis Brazma[7], Paul Brennan[456], **Mark Lathrop#**[455], Louis Letourneau[457], Yasser Riazalhosseini[455], Ghislaine Scelo[456], **Jörg Tost#**[458], Naveen Vasudev[459] and Juris Viksna[460]

PCAWG Tumour-specific providers (breast cancer) in the EU & United Kingdom

Sung-Min Ahn[461], Ludmil B Alexandrov[1,70,302], Samuel Aparicio[462], Laurent Arnould[463], MR Aure[464], Shriram G Bhosle[1], Ewan Birney[7], Ake Borg[465], Sandrine Boyault[466], Arie B Brinkman[467], Jane E Brock[468], Annegien Broeks[469], Adam P Butler[1], Anne-Lise Børresen-Dale[464,470], Carlos Caldas[278,471], Peter J Campbell[1,2], Suet-Feung Chin[278,471], Helen Davies[1,309,310], Christine Desmedt[472,473], Luc Dirix[474], Serge Serge[1], Anna Ehinger[475], Jorunn E Eyfjord[476], Aquila Fatima[204], John A Foekens[477], P Andrew Futreal[478], Øystein Garred[479,480], Moritz Gerstung[7,8], Dilip D Giri[481], Dominik Glodzik[1], Dorthe Grabau[482], Holmfridur Hilmarsdottir[476], Gerrit K Hooijer[483], Jocelyne Jacquemier[484], Se Jin Jang[485], Jon G Jonasson[476], Jos Jonkers[486], Hyung-Yong Kim[484], Tari A King[487,488,489], Stian Knappskog[1,490], Gu Kong[484], Savitri Krishnamurthy[352,491], Sunil R Lakhani[492], Anita Langerød[464], Denis Larsimont[493], Hee Jin Lee[485], Jeong-Yeon Lee[494], Ming Ta Michael Lee[478], Yilong Li[1], Ole Christian Lingjærde[495], Gaetan MacGrogan[496], John WM Martens[477], Sancha Martin[1,353], Iñigo Martincorena[1], Andrew Menzies[1], Sandro Morganella[1], Ville Mustonen[305,306,307], Serena Nik-Zainal[1,309,310,311], Sarah O'Meara[1], Iris Pauporté[20], Sarah Pinder[497], Xavier Pivot[498], Elena Provenzano[499], Colin A Purdie[500], Keiran M Raine[1], Manasa Ramakrishna[1], Kamna Ramakrishnan[1], Jorge Reis-Filho[481], Andrea L Richardson[204], Markus Ringnér[501], Javier Bartolomé

Rodriguez[45], F Germán Rodríguez-González[502], Gilles Romieu[503], Roberto Salgado[394], Torill Sauer[495], Rebecca Shepherd[1], Anieta M Sieuwerts[477], Peter T Simpson[492], Marcel Smid[477], Christos Sotiriou[53], Paul N Span[504], Lucy Stebbings[1], Ólafur Andri Stefánsson[505], Alasdair Stenhouse[506], **Michael R Stratton**#[1], Henk G Stunnenberg[235,507], Fred Sweep[508], Benita Kiat Tee Tan[509], Jon W Teague[1], Gilles Thomas[510], Alastair M Thompson[506], Stefania Tommasi[511], Isabelle Treilleux[512,513], Andrew Tutt[204], Naoto T Ueno[514], Steven Van Laere[474], Peter Van Loo[60,61], Gert G Van den Eynden[474], Peter Vermeulen[474], Alain Viari[400], Anne Vincent-Salomon[507], David C Wedge[1,340,341], Bernice H Wong[515], Lucy Yates[1], Xueqing Zou[1], Carolien HM van Deurzen[516], Marc J van de Vijver[394] and L van't Veer[517]

PCAWG Tumour-specific providers (malignant lymphoma) in Germany

Ole Ammerpohl[518,519], Sietse Aukema[520,521], Anke K Bergmann[522], Stephan H Bernhart[260,261,264], Hans Binder[260,261], Arndt Borkhardt[523], Christoph Borst[524], Benedikt Brors[79,112,262], Birgit Burkhardt[525], Alexander Claviez[526], Roland Eils[50,52,63,64], Maria Elisabeth Goebler[527], Andrea Haake[518], Siegfried Haas[524], Martin Hansmann[528], Jessica I Hoell[523], Steve Hoffmann[260,261,263,264], Michael Hummel[529], Daniel Hübschmann[52,63,80,81,82], Dennis Karsch[530], Wolfram Klapper[520], Kortine Kleinheinz[50,52], Michael Kneba[530], Jan O Korbel[7,8], Helene Kretzmer[261,264], Markus Kreuz[531], Dieter Kube[532], Ralf Küppers[533], Chris Lawerenz[64], Dido Lenze[529], Peter Lichter[77,386], Markus Loeffler[531], Cristina López[246,518], Luisa Mantovani-Löffler[534], Peter Möller[535], German Ott[536], Bernhard Radlwimmer[386], Julia Richter[518,520], Marius Rohde[537], Philip C Rosenstiel[538], Andreas Rosenwald[539], Markus B Schilhabel[538], Matthias Schlesner[50,102], Stefan Schreiber[540], **Reiner Siebert**#[245,246], Peter F Stadler[260,261,264], Peter Staib[541], Stephan Stilgenbauer[542], Stephanie Sungalee[8], Monika Szczepanowski[520], Umut H Toprak[52,543], Lorenz HP Trümper[532], Rabea Wagener[246,518] and Thorsten Zenz[79]

PCAWG Tumour-specific providers (paediatric brain cancer) in Germany

Ivo Buchhalter[50,51,52], Juergen Eils[63,64], Roland Eils[50,52,63,64], Volker Hovestadt[386], Barbara Hutter[77,78,79], David TW Jones[285,286], Natalie Jäger[50], Christof von Kalle[81], Marcel Kool[93,285], Jan O Korbel[7,8], Andrey Korshunov[93], Pablo Landgraf[544,545], Chris Lawerenz[64], Hans Lehrach[546], **Peter Lichter**#[77,386], Paul A Northcott[547], Stefan M Pfister[93,285,548], Bernhard Radlwimmer[386], Guido Reifenberger[545], Matthias Schlesner[50,102], Hans-Jörg Warnatz[546], Joachim Weischenfeldt[8,107,108], Stephan Wolf[549], Marie-Laure Yaspo[546] and Marc Zapatka[386]

PCAWG Tumour-specific providers (prostate cancer) in Germany

Yassen Assenov[550], Benedikt Brors[79,112,262], Juergen Eils[63,64], Roland Eils[50,52,63,64], Lars Feuerbach[112], Clarissa Gerhauser[268], Jan O Korbel[7,8], Chris Lawerenz[64], Hans Lehrach[546], Sarah Minner[551],

Christoph Plass[268], **Guido Sauter#**[552], Thorsten Schlomm[107,553], Nikos Sidiropoulos[108], Ronald Simon[552], **Holger Sültmann#**[79,554], Hans-Jörg Warnatz[546], Dieter Weichenhan[268], Joachim Weischenfeldt[8,107,108] and Marie-Laure Yaspo[546]

PCAWG Tumour-specific providers (oral cancer) in India

Nidhan K Biswas[555], Luca Landoni[396], Arindam Maitra[555], **Partha P Majumder#**[555] and **Rajiv Sarin#**[556]

PCAWG Tumour-specific providers (pancreatic cancer) in Italy

Davide Antonello[396], Stefano Barbi[403], Claudio Bassi[396], Samantha Bersani[399], Giada Bonizzato[400], Cinzia Cantù[400], Ivana Cataldo[399,400], Sara Cingarlini[36], Vincenzo Corbo[400,403], Maria Vittoria Davi[404], Angelo P Dei Tos[557], Matteo Fassan[558], Sonia Grimaldi[400], Luca Landoni[396], Rita T Lawlor[400], Claudio Luchini[399], Andrea Mafficini[400], Giuseppe Malleo[396], Giovanni Marchegiani[396], Michele Milella[36], Marco Miotto[396], Salvatore Paiella[396], Antonio Pea[396], Paolo Pederzoli[396], Borislav C Rusev[400], Andrea Ruzzenente[396], Roberto Salvia[396], Maria Scardoni[399], **Aldo Scarpa#**[400], Elisabetta Sereni[396], Michele Simbolo[403], Nicola Sperandio[400], Giampaolo Tortora[36,37] and Caterina Vicentini[400]

PCAWG Tumour-specific providers (biliary tract cancer) in Japan

Yasuhito Arai[33], Natsuko Hama[33], Nobuyoshi Hiraoka[559], Fumie Hosoda[33], Mamoru Kato[351], Hiromi Nakamura[33], Hidenori Ojima[560], Takuji Okusaka[561], **Tatsuhiro Shibata#**[33,34], Yasushi Totoki[33] and Tomoko Urushidate[34]

PCAWG Tumour-specific providers (gastric cancer) in Japan

Hiroyuki Aburatani#[256], Yasuhito Arai[33], Masashi Fukayama[562], Natsuko Hama[33], Fumie Hosoda[33], Shumpei Ishikawa[563], Hitoshi Katai[564], Mamoru Kato[351], Hiroto Katoh[563], Daisuke Komura[563], Genta Nagae[256,267], Hiromi Nakamura[33], Hirofumi Rokutan[351], Mihoko Saito-Adachi[351], **Tatsuhiro Shibata#**[33,34], Akihiro Suzuki[256,565], Hirokazu Taniguchi[34], Kenji Tatsuno[256], Yasushi Totoki[33], Tetsuo Ushiku[562], Shinichi Yachida[33,566] and Shogo Yamamoto[256]

PCAWG Tumour-specific providers (liver cancer) in Japan

Hiroyuki Aburatani[256], Hiroshi Aikata[567], Koji Arihiro[567], Shun-ichi Ariizumi[568], Keith A Boroevich[46,47], Kazuaki Chayama[567], Akihiro Fujimoto[47], Masashi Fujita[47], Mayuko Furuta[47], Kunihito Gotoh[569], Natsuko Hama[33], Takanori Hasegawa[83], Shinya Hayami[570], Shuto Hayashi[83], Satoshi Hirano[571], Seiya Imoto[83], Mamoru Kato[351], Yoshiiku Kawakami[567], Kazuhiro Maejima[47],

Satoru Miyano[83], Genta Nagae[256,267], **Hidewaki Nakagawa#**[47], Hiromi Nakamura[33], Toru Nakamura[571], Kaoru Nakano[47], Hideki Ohdan[567], Yasushi Rino[572], Aya Sasaki-Oku[47], **Tatsuhiro Shibata#**[33,34], Yuichi Shiraishi[83], Hiroko Tanaka[83], Yasushi Totoki[33], Tatsuhiko Tsunoda[46,207,208,209], Masaki Ueno[570], Rui Yamaguchi[83], Masakazu Yamamoto[568] and Hiroki Yamaue[570]

PCAWG Tumour-specific providers (biliary tract cancer) in Singapore

Su Pin Choo[573], Ioana Cutcutache[251,304], Narong Khuntikeo[396,574], John R McPherson[251,304], Choon Kiat Ong[575], Chawalit Pairojkul[394], Irinel Popescu[576], **Steven G Rozen#**[251,252,304], **Patrick Tan#**[239,250,251,252] and **Bin Tean Teh#**[250,251,252,253,254]

PCAWG Tumour-specific providers (blood cancer) in South Korea

Keun Soo Ahn[577], Hyung-Lae Kim[27], Youngil Koh[291,292] and **Sung-Soo Yoon#**[292]

PCAWG Tumour-specific providers (chronic lymphocytic leukaemia) in Spain

Marta Aymerich[578], **Elias Campo#**[579,580], Josep L L Gelpi[45,67], Ivo G Gut[127,128], Marta Gut[127,128], Armando Lopez-Guillermo[581], Carlos López-Otín[582], Xose S Puente[582], Romina Royo[45] and David Torrents[45,105]

PCAWG Tumour-specific providers (bone cancer) in the United Kingdom

Fernanda Amary[583], Daniel Baumhoer[584], Sam Behjati[1], Bodil Bjerkehagen[584,585], **Peter J Campbell#**[1,2], **Adrienne M Flanagan#**[586], P Andrew Futreal[478], Ola Myklebost[490], Nischalan Pillay[587], Patrick Tarpey[588], Roberto Tirabosco[589] and Olga Zaikova[590]

PCAWG Tumour-specific providers (chronic myeloid disorders) in the United Kingdom

Jacqueline Boultwood[591], David T Bowen[1], Adam P Butler[1], **Peter J Campbell#**[1,2], Mario Cazzola[592], Carlo Gambacorti-Passerini[177], Anthony R Green[279], Eva Hellstrom-Lindberg[593], Luca Malcovati[592], Sancha Martin[1,353], Jyoti Nangalia[1], Elli Papaemmanuil[2] and Paresh Vyas[295,594]

PCAWG Tumour-specific providers (oesophageal cancer) in the

United Kingdom

Yeng Ang[595], Hugh Barr[596], Duncan Beardsmore[597], Matthew Eldridge[278], **Rebecca C Fitzgerald#**[310], James Gossage[598], Nicola Grehan[310], George B Hanna[599], Stephen J Hayes[600,601], Ted R Hupp[602], David Khoo[603], Jesper Lagergren[593,604], Laurence B Lovat[237], Shona MacRae[375], Maria O'Donovan[310], J Robert O'Neill[605], Simon L Parsons[606], Shaun R Preston[607], Sonia Puig[608], Tom Roques[609], Grant Sanders[234], Sharmila Sothi[610], Simon Tavaré[278], Olga Tucker[611], Richard Turkington[612], Timothy J Underwood[613] and Ian Welch[614]

PCAWG Tumour-specific providers (prostate cancer) in the United Kingdom

Daniel M Berney[615], Johann S De Bono[383], G Steven Bova[316], Daniel S Brewer[381,382], Adam P Butler[1], Declan Cahill[616], Niedzica Camacho[383], **Colin S Cooper#**[382,383,384], Nening M Dennis[616], Tim Dudderidge[616,617], Sandra E Edwards[383], **Rosalind A Eeles#**[383,616], Cyril Fisher[616], Christopher S Foster[618,619], Mohammed Ghori[1], Pelvender Gill[594], Vincent J Gnanapragasam[367,620], Gunes Gundem[187], Freddie C Hamdy[594], Steve Hawkins[278], Steven Hazell[616], William Howat[367], William B Isaacs[621], Katalin Karaszi[594], Jonathan D Kay[237], Vincent Khoo[616], Zsofia Kote-Jarai[383], Barbara Kremeyer[1], Pardeep Kumar[616], Adam Lambert[594], Daniel A Leongamornlert[1,383], Naomi Livni[616], Yong-Jie Lu[615,622], Hayley J Luxton[237], Andy G Lynch[278,279,294], Luke Marsden[594], Charlie E Massie[278], Lucy Matthews[383], Erik Mayer[616,623], Ultan McDermott[1], Sue Merson[383], Thomas J Mitchell[1,279,367], David E Neal[278,367], Anthony Ng[624], David Nicol[616], Christopher Ogden[616], Edward W Rowe[616], Nimish C Shah[367], Jon W Teague[1], Sarah Thomas[616], Alan Thompson[616], Peter Van Loo[60,61], Clare Verrill[594,625], Tapio Visakorpi[316], Anne Y Warren[367,626], David C Wedge[1,340,341], Hayley C Whitaker[237], Jorge Zamora[1,270,271,272], Hongwei Zhang[622] and Nicholas van As[616]

PCAWG Tumour-specific providers (TCGA) in the United States

Adam Abeshouse[187], Nishant Agrawal[627], Rehan Akbani[310,376], Hikmat Al-Ahmadie[187], Monique Albert[428], Kenneth Aldape[352,628], Adrian Ally[629], Yeng Ang[595], Elizabeth L Appelbaum[131,237], Joshua Armenia[630], Sylvia Asa[441,606], J Todd Auman[631], Matthew H Bailey[130,131], Miruna Balasundaram[629], Saianand Balu[234], Jill Barnholtz-Sloan[632,633], Hugh Barr[596], John Bartlett[427,428], Oliver F Bathe[634,635], Stephen B Baylin[320,617], Duncan Beardsmore[597], Christopher Benz[636], Andrew Berchuck[637], Benjamin P Berman[257,258,259], Rameen Beroukhim[3,6,163], Mario Berrios[638], Darell Bigner[639], Michael Birrer[100], Tom Bodenheimer[234], Lori Boice[608], Moiz S Bootwalla[638], Marcus Bosenberg[640], Reanne Bowlby[629], Jeffrey Boyd[641], Russell R Broaddus[352], Malcolm Brock[642], Denise Brooks[629], Susan Bullman[3,163], Samantha J Caesar-Johnson[39], Thomas E Carey[643], Rebecca Carlsen[629], Robert Cerfolio[644], Vishal S Chandan[645], Hsiao-Wei Chen[595,630], Andrew D Cherniack[3,48,163], Jeremy Chien[646], Juok Cho[3], Eric Chuah[629], Carrie Cibulskis[3], Kristian Cibulskis[3], Leslie Cope[320], Matthew G Cordes[131,609], Kyle Covington[155], Erin Curley[647], Bogdan Czerniak[352,603], Ludmila Danilova[320], Ian J Davis[648], Timothy Defreitas[3], John A Demchok[39], Noreen Dhalla[629], Rajiv Dhir[649], Li Ding[130,131,138], HarshaVardhan Doddapaneni[155], Adel El-Naggar[352,603], Ina Felau[39], Martin L Ferguson[650], Gaetano

Finocchiaro[651], Kwun M Fong[652], Scott Frazer[3], William Friedman[653], Catrina C Fronick[131,609], Lucinda A Fulton[131], Robert S Fulton[130,131,138], Stacey B Gabriel[3], Jianjiong Gao[630], Nils Gehlenborg[3,654], Jeffrey E Gershenwald[655,656], Gad Getz[3,4,5,6], Ronald Ghossein[481], Nasra H Giama[657], Richard A Gibbs[155], Carmen Gomez[658], James Gossage[598], Ramaswamy Govindan[130], Nicola Grehan[310], George B Hanna[599], D Neil Hayes[234,659,660], Stephen J Hayes[600,601], Apurva M Hegde[375,376], David I Heiman[3], Zachary Heins[187], Austin J Hepperla[234], Katherine A Hoadley[233,234], Andrea Holbrook[638], Robert A Holt[629], Alan P Hoyle[234], Ralph H Hruban[320], Jianhong Hu[155], Mei Huang[608], David Huntsman[661], Ted R Hupp[602], Jason Huse[187], **Carolyn M Hutter#**[23], Christine A Iacobuzio-Donahue[481], Michael Ittmann[662,663,664], Joy C Jayaseelan[155], Stuart R Jefferys[234], Corbin D Jones[665], Steven JM Jones[629], Hartmut Juhl[666], Koo Jeong Kang[667], Beth Karlan[668], Katayoon Kasaian[629], Electron Kebebew[669,670], David Khoo[603], Hark Kyun Kim[671], Jaegil Kim[3], Tari A King[487,488,489], Viktoriya Korchina[155], Ritika Kundra[595,630], Jesper Lagergren[593,604], Phillip H Lai[638], Peter W Laird[265], Eric Lander[3], Michael S Lawrence[3,46,100], Alexander J Lazar[158,352], Xuan Le[672], Darlene Lee[673], Douglas A Levine[187,674], Lora Lewis[155], Tim Ley[675], Haiyan Irene Li[673], Pei Lin[3], W M Linehan[676], Eric Minwei Liu[110,111,187], Fei Fei Liu[368], Laurence B Lovat[237], Yiling Lu[376], Lisa Lype[677], Yussanne Ma[673], Shona MacRae[375], Dennis T Maglinte[638,678], Elaine R Mardis[131,641,679], Jeffrey Marks[396,680], Marco A Marra[673], Thomas J Matthew[11], Michael Mayo[673], Karen McCune[681], Michael D McLellan[130,131,138], Samuel R Meier[3], Shaowu Meng[234], Matthew Meyerson[3,6,48], Piotr A Mieczkowski[233], Tom Mikkelsen[682], Christopher A Miller[131], Gordon B Mills[683], Richard A Moore[673], Carl Morrison[394,684], Lisle E Mose[234], Catherine D Moser[657], Andrew J Mungall[673], Karen Mungall[673], David Mutch[685], Donna M Muzny[155], Jerome Myers[686], Yulia Newton[11], Michael S Noble[3], Peter O'Donnell[687], Brian Patrick O'Neill[688], Angelica Ochoa[187], Akinyemi I Ojesina[240,241,242], Joong-Won Park[689], Joel S Parker[690], Simon L Parsons[606], Harvey Pass[691], Alessandro Pastore[85], Chandra Sekhar Pedamallu[3,6,163], Nathan A Pennell[692], Charles M Perou[234,690,693], Gloria M Petersen[438], Nicholas Petrelli[694], Olga Potapova[695], Shaun R Preston[607], Sonia Puig[608], Janet S Rader[696], Suresh Ramalingam[697], W Kimryn Rathmell[698], Victor Reuter[481], Sheila M Reynolds[677], Matthew Ringel[699], Jeffrey Roach[700], Lewis R Roberts[657], A Gordon Robertson[673], Tom Roques[609], Mark A Rubin[183,197,198,199,200], Sara Sadeghi[673], Gordon Saksena[3], Charles Saller[701], Francisco Sanchez-Vega[595,630], Chris Sander[85,202,203], Grant Sanders[234], Dirk Schadendorf[77,702], Jacqueline E Schein[673], Heather K Schmidt[131], Nikolaus Schultz[630], Steven E Schumacher[3,204], Richard A Scolyer[409,413,419], Raja Seethala[703], Yasin Senbabaoglu[85], Troy Shelton[647], Yan Shi[234], Juliann Shih[3,163], Ilya Shmulevich[677], Craig Shriver[704], Sabina Signoretti[163,168,705], Janae V Simons[234], Samuel Singer[396,706], Payal Sipahimalani[673], Tara J Skelly[233], Karen Smith-McCune[681], Nicholas D Socci[85], Heidi J Sofia[23], Matthew G Soloway[690], Anil K Sood[707,708,709], Sharmila Sothi[610], Angela Tam[673], Donghui Tan[233], Roy Tarnuzzer[39], Nina Thiessen[673], R Houston Thompson[710], Leigh B Thorne[608], Ming Tsao[437,606], Olga Tucker[611], Richard Turkington[612], Christopher Umbricht[274,597,711], Timothy J Underwood[613], David J Van Den Berg[638], Erwin G Van Meir[712], Umadevi Veluvolu[233], Douglas Voet[3], Jiayin Wang[131,145,152], Linghua Wang[155], Zhining Wang[39], Paul Weinberger[713], John N Weinstein[375,376], Daniel J Weisenberger[638], Ian Welch[614], David A Wheeler[154,155], Dennis Wigle[714], Matthew D Wilkerson[233], Richard K Wilson[131,715], Boris Winterhoff[716], Maciej Wiznerowicz[717,718], Tina Wong[131,673], Winghing Wong[719], Liu Xi[155], Liming Yang[39], Christina Yau[636], Venkata D Yellapantula[138,157], **Jean C Zenklusen#**[39], Hailei Zhang[3], Hongxin Zhang[630] and Jiashan Zhang[39]

# Denotes **working group or project co-leader**

Author Affiliations

1. Wellcome Sanger Institute, Wellcome Genome Campus, Hinxton CB10 1SA, UK.
2. Department of Haematology, University of Cambridge, Cambridge CB2 2XY, UK.
3. Broad Institute of MIT and Harvard, Cambridge, MA 02142, USA.
4. Center for Cancer Research, Massachusetts General Hospital, Boston, MA 02129, USA.
5. Department of Pathology, Massachusetts General Hospital, Boston, MA 02115, USA.
6. Harvard Medical School, Boston, MA 02115, USA.
7. European Molecular Biology Laboratory, European Bioinformatics Institute (EMBL-EBI), Cambridge CB10 1SD, UK.
8. Genome Biology Unit, European Molecular Biology Laboratory (EMBL), Heidelberg 69117, Germany.
9. Computational Biology Program, Ontario Institute for Cancer Research, Toronto, ON M5G 0A3, Canada.
10. Department of Molecular Genetics, University of Toronto, Toronto, ON M5S 1A8, Canada.
11. Department of Biomolecular Engineering, University of California Santa Cruz, Santa Cruz, CA 95064, USA.
12. UC Santa Cruz Genomics Institute, University of California Santa Cruz, Santa Cruz, CA 95064, USA.
13. International Cancer Genome Consortium (ICGC)/ICGC Accelerating Research in Genomic Oncology (ARGO) Secretariat, Ontario Institute for Cancer Research, Toronto, ON M5G 0A3, Canada.
14. King Faisal Specialist Hospital and Research Centre, Al Maather, Riyadh 12713, Saudi Arabia.
15. DLR Project Management Agency, Bonn 53227, Germany.
16. Genome Canada, Ottawa, ON K2P 1P1, Canada.
17. Instituto Carlos Slim de la Salud, Mexico City, Mexico.
18. Federal Ministry of Education and Research, Berlin 10117, Germany.
19. Institut Gustave Roussy, Villejuif 94805, France.
20. Institut National du Cancer (INCA), Boulogne-Billancourt 92100, France.
21. The Wellcome Trust, London NW1 2BE, UK.
22. Prostate Cancer Canada, Toronto, ON M5C 1M1, Canada.
23. National Human Genome Research Institute, National Institutes of Health, Bethesda, MD 20892, USA.
24. Department of Biotechnology, Ministry of Science and Technology, Government of India, New Delhi, Delhi 110003, India.
25. Science Writer, Garrett Park, MD 20896, USA.
26. Cancer Research UK, London EC1V 4AD, UK.
27. Department of Biochemistry, College of Medicine, Ewha Womans University, Seoul 07895, South Korea.
28. Chinese Cancer Genome Consortium, Shenzhen 518083, China.
29. Department of Medical Oncology, Beijing Hospital, Beijing 100730, China.
30. Laboratory of Molecular Oncology, Key Laboratory of Carcinogenesis and Translational Research (Ministry of Education), Peking University Cancer Hospital and Institute, Beijing 100142, China.
31. National Cancer Center, Tokyo 104-0045, Japan.

32. German Cancer Aid, Bonn 53113, Germany.
33. Division of Cancer Genomics, National Cancer Center Research Institute, National Cancer Center, Tokyo 104-0045, Japan.
34. Laboratory of Molecular Medicine, Human Genome Center, Institute of Medical Science, University of Tokyo, Tokyo 108-8639, Japan.
35. Japan Agency for Medical Research and Development, Tokyo 100-0004, Japan.
36. Medical Oncology, University and Hospital Trust of Verona, Verona 37134, Italy.
37. University of Verona, Verona 37129, Italy.
38. BGI-Shenzhen, Shenzhen 518083, China.
39. National Cancer Institute, National Institutes of Health, Bethesda, MD 20892, USA.
40. Centre for Law and Genetics, University of Tasmania, Sandy Bay Campus, Hobart, TAS 7001, Australia.
41. Centre of Genomics and Policy, McGill University and Génome Québec Innovation Centre, Montreal, QC H3A 1A4, Canada.
42. Heidelberg Academy of Sciences and Humanities, Heidelberg 69120, Germany.
43. CAPHRI Research School, Maastricht University, Maastricht, ER 6200MD, The Netherlands.
44. Genome Informatics Program, Ontario Institute for Cancer Research, Toronto, ON M5G 0A3, Canada.
45. Barcelona Supercomputing Center (BSC), Barcelona 08034, Spain.
46. Laboratory for Medical Science Mathematics, RIKEN Center for Integrative Medical Sciences, Yokohama 230-0045, Japan.
47. RIKEN Center for Integrative Medical Sciences, Yokohama 230-0045, Japan.
48. Department of Medical Oncology, Dana-Farber Cancer Institute, Boston, MA 02215, USA.
49. Biomedical Engineering, Oregon Health and Science University, Portland, OR 97239, USA.
50. Division of Theoretical Bioinformatics, German Cancer Research Center (DKFZ), Heidelberg 69120, Germany.
51. Heidelberg Center for Personalized Oncology (DKFZ-HIPO), German Cancer Research Center, Heidelberg 69120, Germany.
52. Institute of Pharmacy and Molecular Biotechnology and BioQuant, Heidelberg University, Heidelberg 69120, Germany.
53. University of California San Diego, San Diego, CA 92093, USA.
54. PDXen Biosystems Inc, Seoul 4900, South Korea.
55. Electronics and Telecommunications Research Institute, Daejeon 34129, South Korea.
56. Seven Bridges Genomics, Charlestown, MA 02129, USA.
57. Annai Systems, Inc, Carlsbad, CA 92013, USA.
58. Department of Biomedical Data Science, Stanford University School of Medicine, Stanford, CA 94305, USA.
59. Department of Genetics, Stanford University School of Medicine, Stanford, CA 94305, USA.
60. The Francis Crick Institute, London NW1 1AT, UK.
61. University of Leuven, Leuven B-3000, Belgium.
62. The Hospital for Sick Children, Toronto, ON M5G 0A4, Canada.
63. Heidelberg University, Heidelberg 69120, Germany.
64. New BIH Digital Health Center, Berlin Institute of Health (BIH) and Charité - Universitätsmedizin Berlin, Berlin 10117, Germany.
65. Department of Biochemistry and Molecular Medicine, University of Montreal, Montreal, QC

H3C 3J7, Canada.
66. CIBIO/InBIO - Research Center in Biodiversity and Genetic Resources, Universidade do Porto, Vairão 4485-601, Portugal.
67. Department Biochemistry and Molecular Biomedicine, University of Barcelona, Barcelona 08028, Spain.
68. Department of Medicine, Section of Hematology/Oncology, University of Chicago, Chicago, IL 60637, USA.
69. Department of Medicine, Division of Biomedical Informatics, UC San Diego School of Medicine, San Diego, CA 92093, USA.
70. UC San Diego Moores Cancer Center, San Diego, CA 92093, USA.
71. Children's Hospital of Philadelphia, Philadelphia, PA 19146, USA.
72. Massachusetts General Hospital Center for Cancer Research, Charlestown, MA 02129, USA.
73. University of Melbourne Centre for Cancer Research, Melbourne, VIC 3010, Australia.
74. Syntekabio Inc, Daejon 34025, South Korea.
75. AbbVie, North Chicago, IL 60064, USA.
76. Genomics Research Program, Ontario Institute for Cancer Research, Toronto, ON M5G 0A3, Canada.
77. German Cancer Consortium (DKTK), Heidelberg 69120, Germany.
78. Heidelberg Center for Personalized Oncology (DKFZ-HIPO), German Cancer Research Center (DKFZ), Heidelberg 69120, Germany.
79. National Center for Tumor Diseases (NCT) Heidelberg, Heidelberg 69120, Germany.
80. Department of Pediatric Immunology, Hematology and Oncology, University Hospital, Heidelberg 69120, Germany.
81. German Cancer Research Center (DKFZ), Heidelberg 69120, Germany.
82. Heidelberg Institute for Stem Cell Technology and Experimental Medicine (HI-STEM), Heidelberg 69120, Germany.
83. Institute of Medical Science, University of Tokyo, Tokyo 108-8639, Japan.
84. Genome Integration Data Center, Syntekabio, Inc, Daejon, 34025, South Korea.
85. Computational Biology Center, Memorial Sloan Kettering Cancer Center, New York, NY 10065, USA.
86. Department of Biology, ETH Zurich, Zürich 8093, Switzerland.
87. Department of Computer Science, ETH Zurich, Zurich 8092, Switzerland.
88. SIB Swiss Institute of Bioinformatics, Lausanne 1015, Switzerland.
89. University Hospital Zurich, Zurich 8091, Switzerland.
90. Health Sciences Department of Biomedical Informatics, University of California San Diego, La Jolla, CA 92093, USA.
91. Department of Health Sciences and Technology, Sungkyunkwan University School of Medicine, Seoul 06351, South Korea.
92. Samsung Genome Institute, Seoul 06351, South Korea.
93. Functional and Structural Genomics, German Cancer Research Center (DKFZ), Heidelberg 69120, Germany.
94. Leidos Biomedical Research, Inc, McLean, VA 22102, USA.
95. Center for Biomolecular Science and Engineering, University of California Santa Cruz, Santa Cruz, CA 95064, USA.
96. Sage Bionetworks, Seattle, WA 98109, USA.

97. Department of Cell and Systems Biology, University of Toronto, Toronto, ON M5S 3G5, Canada.
98. Department of Radiation Oncology, University of California San Francisco, San Francisco, CA 94518, USA.
99. CSRA Incorporated, Fairfax, VA 22042, USA.
100. Massachusetts General Hospital, Boston, MA 02114, USA.
101. Weill Cornell Medical College, New York, NY 10065, USA.
102. Bioinformatics and Omics Data Analytics, German Cancer Research Center (DKFZ), Heidelberg 69120, Germany.
103. Institute for Genomics and Systems Biology, University of Chicago, Chicago, IL 60637, USA.
104. Computational Biology Program, School of Medicine, Oregon Health and Science University, Portland, OR 97239, USA.
105. Institució Catalana de Recerca i Estudis Avançats (ICREA), Barcelona 08010, Spain.
106. Department of Clinical and Molecular Medicine, Faculty of Medicine and Health Sciences, Norwegian University of Science and Technology, Trondheim 7030, Norway.
107. Department of Urology, Charité Universitätsmedizin Berlin, Berlin 10117, Germany.
108. Finsen Laboratory and Biotech Research and Innovation Centre (BRIC), University of Copenhagen, Copenhagen 2200, Denmark.
109. Department of Biological Oceanography, Leibniz Institute of Baltic Sea Research, Rostock 18119, Germany.
110. Department of Physiology and Biophysics, Weill Cornell Medicine, New York, NY 10065, USA.
111. Institute for Computational Biomedicine, Weill Cornell Medicine, New York, NY 10021, USA.
112. Division of Applied Bioinformatics, German Cancer Research Center (DKFZ), Heidelberg 69120, Germany.
113. Department of Computer Science, Yale University, New Haven, CT 06520, USA.
114. Department of Molecular Biophysics and Biochemistry, Yale University, New Haven, CT 06520, USA.
115. Program in Computational Biology and Bioinformatics, Yale University, New Haven, CT 06520, USA.
116. Department of Internal Medicine, Stanford University, Stanford, CA 94305, USA.
117. Department of Molecular Medicine (MOMA), Aarhus University Hospital, Aarhus N 8200, Denmark.
118. Clinical Bioinformatics, Swiss Institute of Bioinformatics, Geneva 1202, Switzerland.
119. Institute for Pathology and Molecular Pathology, University Hospital Zurich, Zurich 8091, Switzerland.
120. Institute of Molecular Life Sciences, University of Zurich, Zurich 8057, Switzerland.
121. MIT Computer Science and Artificial Intelligence Laboratory, Massachusetts Institute of Technology, Cambridge, MA 02139, USA.
122. Controlled Department and Institution, New York, NY 10065, USA.
123. Englander Institute for Precision Medicine, Weill Cornell Medicine, New York, NY 10065, USA.
124. Bioinformatics Research Centre (BiRC), Aarhus University, Aarhus 8000, Denmark.
125. Department of Medical Biophysics, University of Toronto, Toronto, ON M5S 1A8, Canada.

126. Institute of Molecular Life Sciences and Swiss Institute of Bioinformatics, University of Zurich, Zurich 8057, Switzerland.
127. CNAG-CRG, Centre for Genomic Regulation (CRG), Barcelona Institute of Science and Technology (BIST), Barcelona 08028, Spain.
128. Universitat Pompeu Fabra (UPF), Barcelona 08003, Spain.
129. Office of Cancer Genomics, National Cancer Institute, National Institutes of Health, Bethesda, MD 20892, USA.
130. Alvin J. Siteman Cancer Center, Washington University School of Medicine, St Louis, MO 63110, USA.
131. The McDonnell Genome Institute at Washington University, St Louis, MO 63108, USA.
132. Computer Network Information Center, Chinese Academy of Sciences, Beijing 100190, China.
133. Center for Digital Health, Berlin Institute of Health and Charitè - Universitätsmedizin Berlin, Berlin 10117, Germany.
134. Department of Human Genetics, University of California Los Angeles, Los Angeles, CA 90095, USA.
135. Department of Pharmacology, University of Toronto, Toronto, ON M5S 1A8, Canada.
136. University of Texas MD Anderson Cancer Center, Houston, TX 77030, USA.
137. Department of Genetics, Informatics Institute, University of Alabama at Birmingham, Birmingham, AL 35294, USA.
138. Department of Medicine and Department of Genetics, Washington University School of Medicine, St. Louis, St Louis, MO 63110, USA.
139. Centre for Genomic Regulation (CRG), The Barcelona Institute of Science and Technology, Barcelona 08003, Spain.
140. Department of Bioinformatics and Computational Biology, The University of Texas MD Anderson Cancer Center, Houston, TX 77030, USA.
141. Department of Urologic Sciences, University of British Columbia, Vancouver, BC V5Z 1M9, Canada.
142. Vancouver Prostate Centre, Vancouver, BC V6H 3Z6, Canada.
143. Division of Life Science and Applied Genomics Center, Hong Kong University of Science and Technology, Clear Water Bay, Hong Kong, China.
144. Geneplus-Shenzhen, Shenzhen 518122, China.
145. School of Computer Science and Technology, Xi'an Jiaotong University, Xi'an 710048, China.
146. Biobyte solutions GmbH, Heidelberg 69126, Germany.
147. Division of Oncology, Washington University School of Medicine, St Louis, MO 63110, USA.
148. Institute of Medical Genetics and Applied Genomics, University of Tübingen, Tübingen 72076, Germany.
149. Indiana University, Bloomington, IN 47405, USA.
150. Simon Fraser University, Burnaby, BC V5A 1S6, Canada.
151. Department of Computer Science, University of Toronto, Toronto, ON M5S 1A8, Canada.
152. School of Electronic and Information Engineering, Xi'an Jiaotong University, Xi'an 710048, China.
153. Department of Genetics, Washington University School of Medicine, St Louis, MO 63110, USA.

154. Department of Molecular and Human Genetics, Baylor College of Medicine, Houston, TX 77030, USA.
155. Human Genome Sequencing Center, Baylor College of Medicine, Houston, TX 77030, USA.
156. The First Affiliated Hospital, Xi'an Jiaotong University, Xi'an 710048, China.
157. Department of Epidemiology and Biostatistics, Memorial Sloan Kettering Cancer Center, New York, NY 10065, USA.
158. Department of Genomic Medicine, The University of Texas MD Anderson Cancer Center, Houston, TX 77030, USA.
159. Quantitative and Computational Biosciences Graduate Program, Baylor College of Medicine, Houston, TX 77030, USA.
160. The Jackson Laboratory for Genomic Medicine, Farmington, CT 06032, USA.
161. Dana-Farber/Boston Children's Cancer and Blood Disorders Center, Boston, MA 02215, USA.
162. Department of Pediatrics, Harvard Medical School, Boston, MA 02115, USA.
163. Department of Medical Oncology, Dana-Farber Cancer Institute, Boston, MA 02115, USA.
164. Department of Mathematics, Aarhus University, Aarhus 8000, Denmark.
165. Center for Biological Sequence Analysis, Department of Bio and Health Informatics, Technical University of Denmark, Lyngby 2800, Denmark.
166. Novo Nordisk Foundation Center for Protein Research, University of Copenhagen, Copenhagen 2200, Denmark.
167. Department for BioMedical Research, University of Bern, Bern 3008, Switzerland.
168. Department of Medical Oncology, Inselspital, University Hospital and University of Bern, Bern 3010, Switzerland.
169. Graduate School for Cellular and Biomedical Sciences, University of Bern, Bern 3012, Switzerland.
170. Department of Urology, Icahn School of Medicine at Mount Sinai, New York, NY 10029, USA.
171. Faculty of Biosciences, Heidelberg University, Heidelberg 69120, Germany.
172. Korea Advanced Institute of Science and Technology, Daejeon 34141, South Korea.
173. Institute for Research in Biomedicine (IRB Barcelona), The Barcelona Institute of Science and Technology, Barcelona 8003, Spain.
174. Research Program on Biomedical Informatics, Universitat Pompeu Fabra, Barcelona 08002, Spain.
175. Department of Cell and Molecular Biology, Science for Life Laboratory, Uppsala University, Uppsala SE-75124, Sweden.
176. Queensland Centre for Medical Genomics, Institute for Molecular Bioscience, University of Queensland, St Lucia, Brisbane, QLD 4072, Australia.
177. University of Milano Bicocca, Monza 20052, Italy.
178. Sir Peter MacCallum Department of Oncology, Peter MacCallum Cancer Centre, University of Melbourne, Melbourne, VIC 3000, Australia.
179. Center for Precision Health, School of Biomedical Informatics, The University of Texas Health Science Center, Houston, TX 77030, USA.
180. The Donnelly Centre, University of Toronto, Toronto, ON M5S 3E1, Canada.
181. Health Data Science Unit, University Clinics, Heidelberg 69120, Germany.
182. Technical University of Denmark, Lyngby 2800, Denmark.

183. Department for Biomedical Research, University of Bern, Bern 3008, Switzerland.
184. Research Core Center, National Cancer Centre Korea, Goyang-si 410-769, South Korea.
185. Institute of Computer Science, Polish Academy of Sciences, Warsawa 01-248, Poland.
186. Harvard University, Cambridge, MA 02138, USA.
187. Memorial Sloan Kettering Cancer Center, New York, NY 10065, USA.
188. Department of Information Technology, Ghent University, Ghent B-9000, Belgium.
189. Department of Plant Biotechnology and Bioinformatics, Ghent University, Ghent B-9000, Belgium.
190. Yale School of Medicine, Yale University, New Haven, CT 06520, USA.
191. Institute for Research in Biomedicine (IRB Barcelona), Barcelona 08028, Spain.
192. Division of Hematology-Oncology, Samsung Medical Center, Sungkyunkwan University School of Medicine, Seoul 06351, South Korea.
193. Samsung Advanced Institute for Health Sciences and Technology, Sungkyunkwan University School of Medicine, Seoul 06351, South Korea.
194. Cheonan Industry-Academic Collaboration Foundation, Sangmyung University, Cheonan 31066, South Korea.
195. Spanish National Cancer Research Centre, Madrid 28029, Spain.
196. Department of Computer Science, Princeton University, Princeton, NJ 08540, USA.
197. Bern Center for Precision Medicine, University Hospital of Bern, University of Bern, Bern 3008, Switzerland.
198. Englander Institute for Precision Medicine, Weill Cornell Medicine and New York Presbyterian Hospital, New York, NY 10021, USA.
199. Meyer Cancer Center, Weill Cornell Medicine, New York, NY 10065, USA.
200. Pathology and Laboratory, Weill Cornell Medical College, New York, NY 10021, USA.
201. Vall d'Hebron Institute of Oncology: VHIO, Barcelona 08035, Spain.
202. cBio Center, Dana-Farber Cancer Institute, Harvard Medical School, Boston, MA 02115, USA.
203. Department of Cell Biology, Harvard Medical School, Boston, MA 02115, USA.
204. Department of Cancer Biology, Dana-Farber Cancer Institute, Boston, MA 02215, USA.
205. Dana-Farber Cancer Institute, Boston, MA 02215, USA.
206. cBio Center, Dana-Farber Cancer Institute, Harvard Medical School, Boston, MA 02215, USA.
207. Core Research for Evolutional Science and Technology (CREST), JST, Tokyo 102-8666, Japan.
208. Department of Biological Sciences, Laboratory for Medical Science Mathematics, Graduate School of Science, University of Tokyo, Yokohama 230-0045, Japan.
209. Department of Medical Science Mathematics, Medical Research Institute, Tokyo Medical and Dental University (TMDU), Tokyo 113-8510, Japan.
210. Department of Oncology-Pathology, Science for Life Laboratory, Karolinska Institutet, Stockholm 17121, Sweden.
211. Department of Gene Technology, Tallinn University of Technology, Tallinn 12616, Estonia.
212. Genetics and Genome Biology Program, SickKids Research Institute, The Hospital for Sick Children, Toronto, ON M5G 1X8, Canada.
213. Department of Information Technology, Ghent University, Interuniversitair Micro-Electronica Centrum (IMEC), Ghent B-9000, Belgium.

214. Department of Immunology, Genetics and Pathology, Science for Life Laboratory, Uppsala University, Uppsala SE-75108, Sweden.
215. Department of Medical Informatics and Clinical Epidemiology, Division of Bioinformatics and Computational Biology, OHSU Knight Cancer Institute, Oregon Health and Science University, Portland, OR 97239, USA.
216. Department of Medicine and Therapeutics, The Chinese University of Hong Kong, Shatin, NT, Hong Kong, China.
217. The University of Texas Health Science Center at Houston, Houston, TX 77030, USA.
218. Department of Biomedical Informatics, College of Medicine, The Ohio State University, Columbus, OH 43210, USA.
219. The Ohio State University Comprehensive Cancer Center (OSUCCC – James), Columbus, OH 43210, USA.
220. The University of Texas School of Biomedical Informatics (SBMI) at Houston, Houston, TX 77030, USA.
221. Department of Biochemistry and Molecular Genetics, Feinberg School of Medicine, Northwestern University, Chicago, IL 60637, USA.
222. Wolfson Wohl Cancer Research Centre, Institute of Cancer Sciences, University of Glasgow, Glasgow G61 1BD, UK.
223. Department of Biomedical Informatics, Harvard Medical School, Boston, MA 02115, USA.
224. Department of Chemistry, Centre for Molecular Science Informatics, University of Cambridge, Cambridge CB2 1EW, UK.
225. Ludwig Center at Harvard Medical School, Boston, MA 02115, USA.
226. Physics Division, Optimization and Systems Biology Lab, Massachusetts General Hospital, Boston, MA 02114, USA.
227. Department of Medicine, Baylor College of Medicine, Houston, TX 77030, USA.
228. Computational and Systems Biology, Genome Institute of Singapore, Singapore 138672, Singapore.
229. School of Computing, National University of Singapore, Singapore 117417, Singapore.
230. The Azrieli Faculty of Medicine, Bar-Ilan University, Safed 13195, Israel.
231. National Cancer Centre Singapore, Singapore 169610, Singapore.
232. Peking University, Beijing 100871, China.
233. Department of Genetics, University of North Carolina at Chapel Hill, Chapel Hill, NC 27599, USA.
234. Lineberger Comprehensive Cancer Center, University of North Carolina at Chapel Hill, Chapel Hill, NC 27599, USA.
235. China National GeneBank-Shenzhen, Shenzhen 518083, China.
236. Berlin Institute for Medical Systems Biology, Max Delbrück Center for Molecular Medicine, Berlin 13125, Germany.
237. University College London, London WC1E 6BT, UK.
238. School of Life Sciences, Peking University, Beijing 100180, China.
239. Genome Institute of Singapore, Singapore 138672, Singapore.
240. Department of Epidemiology, University of Alabama at Birmingham, Birmingham, AL 35294, USA.
241. HudsonAlpha Institute for Biotechnology, Huntsville, AL 35806, USA.
242. O'Neal Comprehensive Cancer Center, University of Alabama at Birmingham, Birmingham,

AL 35294, USA.
243. Department of Biosciences and Nutrition, Karolinska Institutet, Stockholm 14183, Sweden.
244. German Cancer Consortium (DKTK), Partner site Berlin.
245. Human Genetics, University of Kiel, Kiel 24118, Germany.
246. Institute of Human Genetics, Ulm University and Ulm University Medical Center, Ulm 89081, Germany.
247. Computational and Systems Biology Program, Memorial Sloan Kettering Cancer Center, New York, NY 10065, USA.
248. Korea University, Seoul 02481, South Korea.
249. Division of Computational Genomics and Systems Genetics, German Cancer Research Center (DKFZ), Heidelberg 69120, Germany.
250. Cancer Science Institute of Singapore, National University of Singapore, Singapore 169609, Singapore.
251. Programme in Cancer and Stem Cell Biology, Duke-NUS Medical School, Singapore 169857, Singapore.
252. SingHealth, Duke-NUS Institute of Precision Medicine, National Heart Centre Singapore, Singapore 169609, Singapore.
253. Institute of Molecular and Cell Biology, Singapore 169609, Singapore.
254. Laboratory of Cancer Epigenome, Division of Medical Science, National Cancer Centre Singapore, Singapore 169610, Singapore.
255. BIOPIC, ICG and College of Life Sciences, Peking University, Beijing 100871, China.
256. Genome Science Division, Research Center for Advanced Science and Technology, University of Tokyo, Tokyo 153-8904, Japan.
257. Center for Bioinformatics and Functional Genomics, Cedars-Sinai Medical Center, Los Angeles, CA 90048, USA.
258. Department of Biomedical Sciences, Cedars-Sinai Medical Center, Los Angeles, CA 90048, USA.
259. The Hebrew University Faculty of Medicine, Jerusalem 91120, Israel.
260. Department of Computer Science, Bioinformatics Group, University of Leipzig, Leipzig 04109, Germany.
261. Interdisciplinary Center for Bioinformatics, University of Leipzig, Leipzig 04109, Germany.
262. German Cancer Consortium (DKTK), German Cancer Research Center (DKFZ), Heidelberg 69120, Germany.
263. Computational Biology, Leibniz Institute on Aging - Fritz Lipmann Institute (FLI), Jena 07745, Germany.
264. Transcriptome Bioinformatics, LIFE Research Center for Civilization Diseases, University of Leipzig, Leipzig 04109, Germany.
265. Center for Epigenetics, Van Andel Research Institute, Grand Rapids, MI 49503, USA.
266. Institut d'Investigacions Biomèdiques August Pi i Sunyer (IDIBAPS), Barcelona 08036, Spain.
267. Research Center for Advanced Science and Technology, University of Tokyo, Tokyo 108-8639, Japan.
268. Cancer Epigenomics, German Cancer Research Center (DKFZ), Heidelberg 69120, Germany.
269. Van Andel Research Institute, Grand Rapids, MI 49503, USA.
270. Centre for Research in Molecular Medicine and Chronic Diseases (CiMUS), Universidade de Santiago de Compostela, Santiago de Compostela 15706, Spain.

271. Department of Zoology, Genetics and Physical Anthropology, (CiMUS), Universidade de Santiago de Compostela, Santiago de Compostela 15706, Spain.
272. The Biomedical Research Centre (CINBIO), Universidade de Vigo, Vigo 36310, Spain.
273. Department of Veterinary Medicine, Transmissible Cancer Group, University of Cambridge, Cambridge CB3 0ES, UK.
274. Department of Pathology, Johns Hopkins University School of Medicine, Baltimore, MD 21205, USA.
275. McKusick-Nathans Institute of Genetic Medicine, Sidney Kimmel Comprehensive Cancer Center at Johns Hopkins University School of Medicine, Baltimore, MD 21287, USA.
276. Foundation Medicine, Inc, Cambridge, MA 02141, USA.
277. Department of Biochemistry, Microbiology and Immunology, Faculty of Medicine, University of Ottawa, Ottawa, ON K1H 8M5, Canada.
278. Li Ka Shing Centre, Cancer Research UK Cambridge Institute, University of Cambridge, Cambridge CB2 0RE, UK.
279. University of Cambridge, Cambridge CB2 1TN, UK.
280. Quantitative Genomics Laboratories (qGenomics), Barcelona 08950, Spain.
281. Genome Integrity and Structural Biology Laboratory, National Institute of Environmental Health Sciences (NIEHS), Durham, NC 27709, USA.
282. Brandeis University, Waltham, MA 02254, USA.
283. Institute for Computational Biomedicine, Weill Cornell Medical College, New York, NY 10065, USA.
284. New York Genome Center, New York, NY 10013, USA.
285. Hopp Children's Cancer Center (KiTZ), Heidelberg 69120, Germany.
286. Pediatric Glioma Research Group, German Cancer Research Center (DKFZ), Heidelberg 69120, Germany.
287. A.A. Kharkevich Institute of Information Transmission Problems, Moscow 127051, Russia.
288. Oncology and Immunology, Dmitry Rogachev National Research Center of Pediatric Hematology, Moscow 117997, Russia.
289. Skolkovo Institute of Science and Technology, Moscow 121205, Russia.
290. Integrative Bioinformatics Support Group, National Institute of Environmental Health Sciences (NIEHS), Durham, NC 27709, USA.
291. Center For Medical Innovation, Seoul National University Hospital, Seoul 03080, South Korea.
292. Department of Internal Medicine, Seoul National University Hospital, Seoul 03080, South Korea.
293. Division of Genetics and Genomics, Boston Children's Hospital, Harvard Medical School, Boston, MA 02115, USA.
294. School of Medicine/School of Mathematics and Statistics, University of St Andrews, St Andrews, Fife KY16 9SS, UK.
295. Department of Genetics and Computational Biology, QIMR Berghofer Medical Research Institute, Brisbane, QLD 4006, Australia.
296. Institute for Molecular Bioscience, University of Queensland, St Lucia, Brisbane, QLD 4072, Australia.
297. School of Molecular Biosciences and Center for Reproductive Biology, Washington State University, Pullman, WA 99164, USA.

298. Cancer Research Institute, Beth Israel Deaconess Medical Center, Boston, MA 02215, USA.
299. Ben May Department for Cancer Research and Department of Human Genetics, University of Chicago, Chicago, IL 60637, USA.
300. Tri-Institutional PhD Program in Computational Biology and Medicine, Weill Cornell Medicine, New York, NY 10065, USA.
301. Department of Biostatistics and Computational Biology, Dana-Farber Cancer Institute and Harvard Medical School, Boston, MA 02215, USA.
302. Department of Cellular and Molecular Medicine and Department of Bioengineering, University of California San Diego, La Jolla, CA 92093, USA.
303. Department of Cellular and Molecular Medicine and Department of Bioengineering, University of California, San Diego, La Jolla, CA 92093, USA.
304. Centre for Computational Biology, Duke-NUS Medical School, Singapore 169857, Singapore.
305. Department of Computer Science, University of Helsinki, Helsinki 00014, Finland.
306. Institute of Biotechnology, University of Helsinki, Helsinki 00014, Finland.
307. Organismal and Evolutionary Biology Research Programme, University of Helsinki, Helsinki 00014, Finland.
308. Programme in Cancer and Stem Cell Biology, Centre for Computational Biology, Duke-NUS Medical School, Singapore 169857, Singapore.
309. Academic Department of Medical Genetics, University of Cambridge, Addenbrooke's Hospital, Cambridge CB2 0QQ, UK.
310. MRC Cancer Unit, University of Cambridge, Cambridge CB2 0XZ, UK.
311. The University of Cambridge School of Clinical Medicine, Cambridge CB2 0SP, UK.
312. National Centre for Biological Sciences, Tata Institute of Fundamental Research, Bangalore 560065, India.
313. Department of Applied Mathematics and Theoretical Physics, Centre for Mathematical Sciences, University of Cambridge, Cambridge CB3 0WA, UK.
314. Department of Statistics, Columbia University, New York, NY 10027, USA.
315. Duke-NUS Medical School, Singapore 169857, Singapore.
316. Faculty of Medicine and Health Technology, Tampere University and Tays Cancer Center, Tampere University Hospital, Tampere Fl-33014, Finland.
317. Bakar Computational Health Sciences Institute and Department of Pediatrics, University of California, San Francisco, CA 94158-2549, USA.
318. Division of Cancer Epidemiology and Genetics, National Cancer Institute, National Institutes of Health, Bethesda, MD 20892, USA.
319. Department of Biostatistics, Bloomberg School of Public Health, Johns Hopkins University, Baltimore, MD 21205, USA.
320. Department of Oncology, Sidney Kimmel Comprehensive Cancer Center at Johns Hopkins University School of Medicine, Baltimore, MD 21287, USA.
321. Integrated Graduate Program in Physical and Engineering Biology, Yale University, New Haven, CT 06520, USA.
322. Department of Computational Biology, University of Lausanne, Lausanne 1015, Switzerland.
323. Department of Genetic Medicine and Development, University of Geneva Medical School, Geneva CH 1211, Switzerland.

324. Swiss Institute of Bioinformatics, University of Geneva, Geneva CH 1211, Switzerland.
325. Independent Consultant, Wellesley 02481, USA.
326. Department of Oncology, Centre for Cancer Genetic Epidemiology, University of Cambridge, Cambridge CB1 8RN, UK.
327. Department of Public Health and Primary Care, Centre for Cancer Genetic Epidemiology, University of Cambridge, Cambridge CB1 8RN, UK.
328. CIBER Epidemiología y Salud Pública (CIBERESP), Madrid 28029, Spain.
329. Research Group on Statistics, Econometrics and Health (GRECS), UdG, Barcelona 8041, Spain.
330. Oxford Nanopore Technologies, New York, NY 10013, USA.
331. Department of Medical Genetics, College of Medicine, Hallym University, Chuncheon 24252, South Korea.
332. Department of Experimental and Health Sciences, Institute of Evolutionary Biology (UPF-CSIC), Universitat Pompeu Fabra, Barcelona 08003, Spain.
333. Icahn School of Medicine at Mount Sinai, New York, NY 10029, USA.
334. Laboratory of Translational Genomics, Division of Cancer Epidemiology and Genetics, National Cancer Institute, National Institutes of Health, Bethesda, MD 20892, USA.
335. Institut Català de Paleontologia Miquel Crusafont, Universitat Autònoma de Barcelona, Barcelona 08193, Spain.
336. Applications Department, Oxford Nanopore Technologies, Oxford OX4 4DQ, UK.
337. Department of Genetics, Microbiology and Statistics, University of Barcelona, IRSJD, IBUB, Barcelona 08028, Spain.
338. Department of Ophthalmology and Ocular Genomics Institute, Massachusetts Eye and Ear, Harvard Medical School, Boston, MA 02114, USA.
339. Department of Medical and Clinical Genetics, Genome-Scale Biology Research Program, University of Helsinki, Helsinki 00100, Finland.
340. Big Data Institute, Li Ka Shing Centre, University of Oxford, Oxford OX3 7LF, UK.
341. Oxford NIHR Biomedical Research Centre, University of Oxford, Oxford OX4 2PG, UK.
342. School of Electronic Information and Communications, Huazhong University of Science and Technology, Wuhan 430074, China.
343. Bioinformatics Unit, Spanish National Cancer Research Centre (CNIO), Madrid 28029, Spain.
344. Vector Institute, Toronto, ON M5G 0A3, Canada.
345. Cancer Division, Garvan Institute of Medical Research, Kinghorn Cancer Centre, University of New South Wales (UNSW Sydney), Sydney, NSW 2010, Australia.
346. South Western Sydney Clinical School, Faculty of Medicine, University of New South Wales (UNSW Sydney), Liverpool, NSW 2170, Australia.
347. West of Scotland Pancreatic Unit, Glasgow Royal Infirmary, Glasgow G31 2ER, UK.
348. Centre for Cancer Research, Victorian Comprehensive Cancer Centre, University of Melbourne, Melbourne, VIC 3010, Australia.
349. MRC Human Genetics Unit, MRC IGMM, University of Edinburgh, Edinburgh EH4 2XU, UK.
350. Department of Biology, Bioinformatics Group, Division of Molecular Biology, Faculty of Science, University of Zagreb, Zagreb 10000, Croatia.
351. Department of Bioinformatics, Division of Cancer Genomics, National Cancer Center Research Institute, Tokyo 104-0045, Japan.

352. Department of Pathology, The University of Texas MD Anderson Cancer Center, Houston, TX 77030, USA.
353. University of Glasgow, Glasgow G61 1BD, UK.
354. Oregon Health and Science University, Portland, OR 97239, USA.
355. MRC-University of Glasgow Centre for Virus Research, Glasgow G61 1QH, UK.
356. School of Computing Science, University of Glasgow, Glasgow G12 8RZ, UK.
357. Molecular and Medical Genetics, OHSU Knight Cancer Institute, Oregon Health and Science University, Portland, OR 97239, USA.
358. Department of Surgery, University of Melbourne, Parkville, VIC 3010, Australia.
359. The Murdoch Children's Research Institute, Royal Children's Hospital, Parkville, VIC 3052, Australia.
360. Walter and Eliza Hall Institute, Parkville, VIC 3052, Australia.
361. University of Cologne, Cologne 50931, Germany.
362. The Edward S. Rogers Sr. Department of Electrical and Computer Engineering, University of Toronto, Toronto, ON M5S 3G4, Canada.
363. University of Ljubljana, Ljubljana 1000, Slovenia.
364. Department of Public Health Sciences, University of Chicago, Chicago, IL 60637, USA.
365. Research Institute, NorthShore University HealthSystem, Evanston, IL 60201, USA.
366. Department of Statistics, University of California Santa Cruz, Santa Cruz, CA 95064, USA.
367. Cambridge University Hospitals NHS Foundation Trust, Cambridge CB2 0QQ, UK.
368. University of Toronto, Toronto, ON M5G 2M9, Canada.
369. Department of Computer Science, Carleton College, Northfield, MN 55057, USA.
370. Center for Psychiatric Genetics, NorthShore University HealthSystem, Evanston, IL 60201, USA.
371. Argmix Consulting, North Vancouver, BC V7M 2J5, Canada.
372. Department of Biostatistics, The University of Texas MD Anderson Cancer Center, Houston, TX 77030, USA.
373. Department of Biostatistics, University of North Carolina at Chapel Hill, Chapel Hill, NC 27599, USA.
374. Howard Hughes Medical Institute, University of California Santa Cruz, Santa Cruz, CA 95064, USA.
375. Cancer Unit, MRC University of Cambridge, Cambridge CB2 0XZ, UK.
376. Department of Bioinformatics and Computational Biology and Department of Systems Biology, The University of Texas MD Anderson Cancer Center, Houston, TX 77030, USA.
377. Department of Health Sciences, Faculty of Medical Sciences, Kyushu University, Fukuoka 812-8582, Japan.
378. Department of Applied Mathematics and Statistics, Johns Hopkins University, Baltimore, MD 21218, USA.
379. Bioinformatics Core Facility, University Medical Center Hamburg, Hamburg 20246, Germany.
380. Heinrich Pette Institute, Leibniz Institute for Experimental Virology, Hamburg 20251, Germany.
381. Earlham Institute, Norwich NR4 7UZ, UK.
382. Norwich Medical School, University of East Anglia, Norwich NR4 7TJ, UK.
383. The Institute of Cancer Research, London SW7 3RP, UK.

384. University of East Anglia, Norwich NR4 7TJ, UK.
385. German Center for Infection Research (DZIF), Partner Site Hamburg-Borstel-Lübeck-Riems, Hamburg, Germany.
386. Division of Molecular Genetics, German Cancer Research Center (DKFZ), Heidelberg 69120, Germany.
387. Peter MacCallum Cancer Centre, University of Melbourne, Melbourne, VIC 3000, Australia.
388. QIMR Berghofer Medical Research Institute, Brisbane, QLD 4006, Australia.
389. Victorian Institute of Forensic Medicine, Southbank, VIC 3006, Australia.
390. University of Pennsylvania, Philadelphia, PA 19104, USA.
391. Department of Gynaecological Oncology, Westmead Hospital, Sydney, NSW 2145, Australia.
392. Genetics and Molecular Pathology, SA Pathology, Adelaide, SA 5000, Australia.
393. Centre for Cancer Research, Westmead Institute for Medical Research, University of Sydney, Sydney, NSW 2145, Australia.
394. Department of Clinical Pathology, University of Melbourne, Melbourne, VIC 3052, Australia.
395. Faculty of Medicine and Health, University of Sydney, Sydney, NSW 2145, Australia.
396. Department of Surgery, Pancreas Institute, University and Hospital Trust of Verona, Verona 37134, Italy.
397. Department of Surgery, Princess Alexandra Hospital, Brisbane, QLD 4102, Australia.
398. Surgical Oncology Group, Diamantina Institute, University of Queensland, Brisbane, QLD 4102, Australia.
399. Department of Diagnostics and Public Health, University and Hospital Trust of Verona, Verona 37134, Italy.
400. ARC-Net Centre for Applied Research on Cancer, University and Hospital Trust of Verona, Verona 37134, Italy.
401. Illawarra Shoalhaven Local Health District L3 Illawarra Cancer Care Centre, Wollongong Hospital, Wollongong, NSW 2500, Australia.
402. School of Biological Sciences, University of Auckland, Auckland 1010, New Zealand.
403. Department of Pathology and Diagnostics, University and Hospital Trust of Verona, Verona 37134, Italy.
404. Department of Medicine, Section of Endocrinology, University and Hospital Trust of Verona, Verona 37134, Italy.
405. Department of Pathology, Queen Elizabeth University Hospital, Glasgow G51 4TF, UK.
406. University of Sydney, Sydney, NSW 2006, Australia.
407. Department of Medical Oncology, Beatson West of Scotland Cancer Centre, Glasgow G12 0YN, UK.
408. Academic Unit of Surgery, School of Medicine, College of Medical, Veterinary and Life Sciences, University of Glasgow, Glasgow Royal Infirmary, Glasgow G4 OSF, UK.
409. Tissue Pathology and Diagnostic Oncology, Royal Prince Alfred Hospital, Sydney, NSW 2050, Australia.
410. Discipline of Surgery, Western Sydney University, Penrith, NSW 2751, Australia.
411. Institute of Cancer Sciences, College of Medical Veterinary and Life Sciences, University of Glasgow, Glasgow G12 8QQ, UK.
412. Faculty of Medicine and Health Sciences, Macquarie University, Sydney, NSW 2109,

Australia.
413. Sydney Medical School, University of Sydney, Sydney, NSW 2050, Australia.
414. School of Environmental and Life Sciences, Faculty of Science, The University of Newcastle, Ourimbah, NSW 2258, Australia.
415. Eastern Clinical School, Monash University, Melbourne, VIC 3128, Australia.
416. Epworth HealthCare, Richmond, VIC 3121, Australia.
417. Applied Tumor Genomics Research Program, Research Programs Unit, University of Helsinki, Helsinki 00290, Finland.
418. Olivia Newton-John Cancer Research Institute, La Trobe University, Heidelberg, VIC 3084, Australia.
419. Melanoma Institute Australia, University of Sydney, Sydney, NSW 2065, Australia.
420. Children's Hospital at Westmead, University of Sydney, Sydney, NSW 2145, Australia.
421. Australian Institute of Tropical Health and Medicine, James Cook University, Douglas, QLD 4814, Australia.
422. Bioplatforms Australia, North Ryde, NSW 2109, Australia.
423. Melanoma Institute Australia, Macquarie University, Sydney, NSW 2109, Australia.
424. Children's Medical Research Institute, Sydney, NSW 2145, Australia.
425. Discipline of Pathology, Sydney Medical School, University of Sydney, Sydney, NSW 2065, Australia.
426. School of Mathematics and Statistics, University of Sydney, Sydney, NSW 2006, Australia.
427. Diagnostic Development, Ontario Institute for Cancer Research, Toronto, ON M5G 0A3, Canada.
428. Ontario Tumour Bank, Ontario Institute for Cancer Research, Toronto, ON M5G 0A3, Canada.
429. PanCuRx Translational Research Initiative, Ontario Institute for Cancer Research, Toronto, ON M5G 0A3, Canada.
430. UHN Program in BioSpecimen Sciences, Toronto General Hospital, Toronto, ON M5G 2C4, Canada.
431. Hepatobiliary/Pancreatic Surgical Oncology Program, University Health Network, Toronto, ON M5G 2C4, Canada.
432. Lunenfeld-Tanenbaum Research Institute, Mount Sinai Hospital, Toronto, ON M5G 1X5, Canada.
433. Division of Medical Oncology, Princess Margaret Cancer Centre, Toronto, ON M5G 2M9, Canada.
434. University of Nebraska Medical Center, Omaha, NE 68198-6880, USA.
435. Transformative Pathology, Ontario Institute for Cancer Research, Toronto, ON M5G 0A3, Canada.
436. Department of Biochemistry and Molecular Medicine, University California at Davis, Sacramento, CA 95817, USA.
437. University Health Network, Princess Margaret Cancer Centre, Toronto, ON M5G 1L7, Canada.
438. Department of Health Sciences Research, Mayo Clinic, Rochester, MN 55905, USA.
439. Department of Laboratory Medicine and Pathobiology, University of Toronto, Toronto, ON M5S 1A8, Canada.
440. Department of Pathology, Human Oncology and Pathogenesis Program, Memorial Sloan

Kettering Cancer Center, New York, NY 10053, USA.
441. Department of Pathology, University Health Network, Toronto General Hospital, Toronto, ON M5G 2C4, Canada.
442. Human Longevity Inc, San Diego, CA 92121, USA.
443. CRUKManchester Institute and Centre, Manchester M20 4GJ, UK.
444. Department of Radiation Oncology, University of Toronto, Toronto, ON M5S 1A8, Canada.
445. Division of Cancer Sciences, Manchester Cancer Research Centre, University of Manchester, Manchester M20 4GJ, UK.
446. Radiation Medicine Program, Princess Margaret Cancer Centre, Toronto, ON M5G 2M9, Canada.
447. Department of Surgical Oncology, Princess Margaret Cancer Centre, Toronto, ON M5G 2M9, Canada.
448. STTARR Innovation Facility, Princess Margaret Cancer Centre, Toronto, ON M5G 1L7, Canada.
449. Hefei University of Technology, Anhui 230009, China.
450. State Key Laboratory of Cancer Biology, and Xijing Hospital of Digestive Diseases, Fourth Military Medical University, Shaanxi 710032, China.
451. Fourth Military Medical University, Shaanxi 710032, China.
452. Department of Surgery, Ruijin Hospital, Shanghai Jiaotong University School of Medicine, Shanghai 200025, China.
453. Leeds Institute of Medical Research, University of Leeds, St James's University Hospital, Leeds LS9 7TF, UK.
454. Canadian Center for Computational Genomics, McGill University, Montreal, QC H3A 0G1, Canada.
455. Department of Human Genetics, McGill University, Montreal, QC H3A 1B1, Canada.
456. International Agency for Research on Cancer, Lyon 69008, France.
457. McGill University and Genome Quebec Innovation Centre, Montreal, QC H3A 0G1, Canada.
458. Centre National de Génotypage, CEA - Institute de Génomique, Evry 91000, France.
459. Leeds Institute of Medical Research @ St James's, University of Leeds, St James’s University Hospital, Leeds LS9 7TF, UK.
460. Institute of Mathematics and Computer Science, University of Latvia, Riga LV 1459, Latvia.
461. Department of Oncology, Gil Medical Center, Gachon University, Incheon 405-760, South Korea.
462. Department of Molecular Oncology, BC Cancer Research Centre, Vancouver, BC V5Z 1L3, Canada.
463. Los Alamos National Laboratory, Los Alamos, NM 87545, USA.
464. Department of Cancer Genetics, Institute for Cancer Research, Oslo University Hospital-Radiumhospitalet, Oslo O310, Norway.
465. Department of Clinical Sciences, Lund, Division of Oncology and Pathology, Skåne University Hospital, Lund University, Lund 223 62, Sweden.
466. Translational Research Lab, Centre Léon Bérard, Lyon 69373, France.
467. Department of Molecular Biology, Faculty of Science, Radboud Institute for Molecular Life Sciences, Radboud University, Nijmegen 6500 HB, The Netherlands.
468. Department of Pathology, Brigham and Women's Hospital, Harvard Medical School, Boston, MA 02115, USA.

469. Department of Molecular Pathology, The Netherlands Cancer Institute, Amsterdam 1066 CX, The Netherlands.
470. Institute of Clinical Medicine, Faculty of Medicine, University of Oslo, Oslo O310, Norway.
471. Department of Oncology, University of Cambridge, Cambridge CB2 1TN, UK.
472. Breast Cancer Translational Research Laboratory JC Heuson, Institut Jules Bordet, Brussels 1000, Belgium.
473. Department of Oncology, Laboratory for Translational Breast Cancer Research, KU Leuven, Leuven 3000, Belgium.
474. Translational Cancer Research Unit, GZA Hospitals St.-Augustinus, Center for Oncological Research, Faculty of Medicine and Health Sciences, University of Antwerp, Antwerp 2000, Belgium.
475. Department of Laboratory Medicine, Translational Cancer Research, Lund University Cancer Center at Medicon Village, Lund University, Lund SE-221 85, Sweden.
476. Icelandic Cancer Registry, Icelandic Cancer Society, Reykjavik 125, Iceland.
477. Department of Medical Oncology, Josephine Nefkens Institute and Cancer Genomics Centre, Erasmus Medical Center, Rotterdam 3015 CN, The Netherlands.
478. National Genotyping Center, Institute of Biomedical Sciences, Academia Sinica, Taipei 115, Taiwan.
479. Department of Pathology, Oslo University Hospital Ulleval, Oslo 0450, Norway.
480. Faculty of Medicine and Institute of Clinical Medicine, University of Oslo, Oslo NO-0316, Norway.
481. Department of Pathology, Memorial Sloan Kettering Cancer Center, New York, NY 10065, USA.
482. Department of Pathology, Skåne University Hospital, Lund University, Lund SE-221 85, Sweden.
483. Department of Pathology, Academic Medical Center, Amsterdam 1105 AZ, The Netherlands.
484. Department of Pathology, College of Medicine, Hanyang University, Seoul 133-791, South Korea.
485. Department of Pathology, Asan Medical Center, College of Medicine, Ulsan University, Songpa-gu, Seoul 05505, South Korea.
486. The Netherlands Cancer Institute, Amsterdam 1066 CX, The Netherlands.
487. Breast Oncology Program, Dana-Farber/Brigham and Women's Cancer Center, Boston, MA 02115, USA.
488. Department of Surgery, Memorial Sloan-Kettering Cancer Center, New York, NY 10065, USA.
489. Division of Breast Surgery, Brigham and Women's Hospital, Boston, MA 02115, USA.
490. Department of Clinical Science, University of Bergen, Bergen 5020, Norway.
491. Morgan Welch Inflammatory Breast Cancer Research Program and Clinic, The University of Texas MD Anderson Cancer Center, Houston, TX 77030, USA.
492. The University of Queensland Centre for Clinical Research, Royal Brisbane and Women's Hospital, Herston, QLD 4029, Australia.
493. Department of Pathology, Institut Jules Bordet, Brussels 1000, Belgium.
494. Institute for Bioengineering and Biopharmaceutical Research (IBBR), Hanyang University, Seoul 133-791, South Korea.

495. University of Oslo, Oslo 0316, Norway.
496. Institut Bergonié, Bordeaux 33076, France.
497. Department of Research Oncology, Guy's Hospital, King's Health Partners AHSC, King's College London School of Medicine, London SE1 9RT, UK.
498. University Hospital of Minjoz, INSERM UMR 1098, Besançon 25000, France.
499. Cambridge Breast Unit, Addenbrooke's Hospital, Cambridge University Hospital NHS Foundation Trust and NIHR Cambridge Biomedical Research Centre, Cambridge CB2 2QQ, UK.
500. East of Scotland Breast Service, Ninewells Hospital, Aberdeen AB25 2XF, UK.
501. Department of Clinical Sciences, Lund, Division of Oncology and Pathology, Lund University, Lund 223 62, Sweden.
502. University of Copenhagen, Copenhagen 2200, Denmark.
503. Oncologie Sénologie, ICM Institut Régional du Cancer, Montpellier 34298, France.
504. Department of Radiation Oncology, Radboud University Nijmegen Medical Centre, Nijmegen 6525 GA, The Netherlands.
505. University of Iceland, Reykjavik 101, Iceland.
506. Dundee Cancer Centre, Ninewells Hospital, Dundee DD2 1SY, UK.
507. Institut Curie, INSERM Unit 830, Paris 75248, France.
508. Department of Laboratory Medicine, Radboud University Nijmegen Medical Centre, Nijmegen 6525 GA, The Netherlands.
509. Department of General Surgery, Singapore General Hospital, Singapore 169608, Singapore.
510. Universite Lyon, INCa-Synergie, Centre Léon Bérard, Lyon 69008, France.
511. Giovanni Paolo II / I.R.C.C.S. Cancer Institute, Bari BA 70124, Italy.
512. Department of Biopathology, Centre Léon Bérard, Lyon 69008, France.
513. Université Claude Bernard Lyon 1, Villeurbanne 69100, France.
514. Breast Medical Oncology, The University of Texas MD Anderson Cancer Center, Houston, TX 77030, USA.
515. NCCS-VARI Translational Research Laboratory, National Cancer Centre Singapore, Singapore 169610, Singapore.
516. Department of Pathology, Erasmus Medical Center Rotterdam, Rotterdam 3015 GD, The Netherlands.
517. Division of Molecular Carcinogenesis, The Netherlands Cancer Institute, Amsterdam 1066 CX, The Netherlands.
518. Institute of Human Genetics, Christian-Albrechts-University, Kiel 24118, Germany.
519. Institute of Human Genetics, Ulm University and Ulm University Medical Center of Ulm, Ulm 89081, Germany.
520. Hematopathology Section, Institute of Pathology, Christian-Albrechts-University, Kiel 24118, Germany.
521. Institute of Human Genetics, University of Ulm and University Hospital of Ulm, Ulm 89081, Germany.
522. Department of Human Genetics, Hannover Medical School, Hannover 30625, Germany.
523. Department of Pediatric Oncology, Hematology and Clinical Immunology, Heinrich-Heine-University, Düsseldorf 40225, Germany.
524. Department of Internal Medicine/Hematology, Friedrich-Ebert-Hospital, Neumünster 24534, Germany.
525. Pediatric Hematology and Oncology, University Hospital Muenster, Muenster 24534,

Germany.
526. Department of Pediatrics, University Hospital Schleswig-Holstein, Kiel 24105, Germany.
527. Department of Medicine II, University of Würzburg, Wuerzburg 97070, Germany.
528. Senckenberg Institute of Pathology, University of Frankfurt Medical School, Frankfurt 60596, Germany.
529. Institute of Pathology, Charité – University Medicine Berlin, Berlin 10117, Germany.
530. Department for Internal Medicine II, University Hospital Schleswig-Holstein, Kiel 24105, Germany.
531. Institute for Medical Informatics Statistics and Epidemiology, University of Leipzig, Leipzig 04109, Germany.
532. Department of Hematology and Oncology, Georg-Augusts-University of Göttingen, Göttingen 37073, Germany.
533. Institute of Cell Biology (Cancer Research), University of Duisburg-Essen, Essen D-45147, Germany.
534. MVZ Department of Oncology, PraxisClinic am Johannisplatz, Leipzig 04109, Germany.
535. Institute of Pathology, Ulm University and University Hospital of Ulm, Ulm 89081, Germany.
536. Department of Pathology, Robert-Bosch-Hospital, Stuttgart, Germany, Stuttgart 70376, Germany.
537. University Hospital Giessen, Pediatric Hematology and Oncology, Giessen 35392, Germany.
538. Institute of Clinical Molecular Biology, Christian-Albrechts-University, Kiel 24118, Germany.
539. Institute of Pathology, University of Wuerzburg, Wuerzburg 97070, Germany.
540. Department of General Internal Medicine, University Kiel, Kiel 24118, Germany.
541. Clinic for Hematology and Oncology, St.-Antonius-Hospital, Eschweiler D-52249, Germany.
542. Department for Internal Medicine III, University of Ulm and University Hospital of Ulm, Ulm 89081, Germany.
543. Neuroblastoma Genomics, German Cancer Research Center (DKFZ), Heidelberg 69120, Germany.
544. Department of Pediatric Oncology and Hematology, University of Cologne, Cologne 50937, Germany.
545. University of Düsseldorf, Düsseldorf 40225, Germany.
546. Department of Vertebrate Genomics/Otto Warburg Laboratory Gene Regulation and Systems Biology of Cancer, Max Planck Institute for Molecular Genetics, Berlin 14195, Germany.
547. St. Jude Children's Research Hospital, Memphis, TN 38105-3678, USA.
548. Heidelberg University Hospital, Heidelberg 69120, Germany.
549. Genomics and Proteomics Core Facility High Throughput Sequencing Unit, German Cancer Research Center (DKFZ), Heidelberg 69120, Germany.
550. Epigenomics and Cancer Risk Factors, German Cancer Research Center (DKFZ), Heidelberg 69120, Germany.
551. Institute of Pathology, University Medical Center Hamburg-Eppendorf, Hamburg 20246, Germany.
552. Institute of Pathology, University Medical Center Hamburg-Eppendorf, Hamburg 20251, Germany.
553. Martini-Clinic, Prostate Cancer Center, University Medical Center Hamburg-Eppendorf, Hamburg 20095, Germany.

554. Division of Cancer Genome Research, German Cancer Research Center (DKFZ), Heidelberg 69120, Germany.
555. National Institute of Biomedical Genomics, Kalyani 741235, West Bengal, India.
556. Advanced Centre for Treatment Research and Education in Cancer, Tata Memorial Centre, Navi Mumbai, Maharashtra 410210, India.
557. Department of Pathology, General Hospital of Treviso, Department of Medicine, University of Padua, Treviso 31100, Italy.
558. Department of Medicine (DIMED), Surgical Pathology Unit, University of Padua, Padua 35121, Italy.
559. Department of Pathology and Clinical Laboratory, National Cancer Center Hospital, Tokyo 104-0045, Japan.
560. Department of Pathology, Keio University School of Medicine, Tokyo 160-8582, Japan.
561. Department of Hepatobiliary and Pancreatic Oncology, National Cancer Center Hospital, Tokyo 104-0045, Japan.
562. Department of Pathology, Graduate School of Medicine, University of Tokyo, Tokyo 113-0033, Japan.
563. Preventive Medicine, Graduate School of Medicine, University of Tokyo, Tokyo 113-0033, Japan.
564. Department of Gastric Surgery, National Cancer Center Hospital, Tokyo 104-0045, Japan.
565. Department of Gastroenterology and Hepatology, Yokohama City University Graduate School of Medicine, Kanagawa 236-0004, Japan.
566. Department of Cancer Genome Informatics, Graduate School of Medicine, Osaka University, Osaka 565-0871, Japan.
567. Hiroshima University, Hiroshima 734-8553, Japan.
568. Tokyo Women's Medical University, Tokyo 162-8666, Japan.
569. Osaka International Cancer Center, Osaka 541-8567, Japan.
570. Wakayama Medical University, Wakayama 641-8509, Japan.
571. Hokkaido University, Sapporo 060-8648, Japan.
572. Department of Surgery, Yokohama City University Graduate School of Medicine, Kanagawa 236-0004, Japan.
573. Division of Medical Oncology, National Cancer Centre, Singapore 169610, Singapore.
574. Cholangiocarcinoma Screening and Care Program and Liver Fluke and Cholangiocarcinoma Research Centre, Faculty of Medicine, Khon Kaen University, Khon Kaen 40002, Thailand.
575. Lymphoma Genomic Translational Research Laboratory, National Cancer Centre, Singapore 169610, Singapore.
576. Center of Digestive Diseases and Liver Transplantation, Fundeni Clinical Institute, Bucharest 022328, Romania.
577. Department of Surgery, Division of Hepatobiliary and Pancreatic Surgery, School of Medicine, Keimyung University Dongsan Medical Center, Daegu 41931, South Korea.
578. Pathology, Hospital Clinic, Institut d'Investigacions Biomèdiques August Pi i Sunyer (IDIBAPS), University of Barcelona, Barcelona 8034, Spain.
579. Anatomia Patológica, Hospital Clinic, Institut d'Investigacions Biomèdiques August Pi i Sunyer (IDIBAPS), University of Barcelona, Barcelona 8036, Spain.
580. Spanish Ministry of Science and Innovation, Madrid 28046, Spain.
581. Hematology, Hospital Clinic, Institut d'Investigacions Biomèdiques August Pi i Sunyer

(IDIBAPS), University of Barcelona, Barcelona 8034, Spain.
582. Department of Biochemistry and Molecular Biology, Faculty of Medicine, University Institute of Oncology-IUOPA, Oviedo 33006, Spain.
583. Royal National Orthopaedic Hospital - Bolsover, London W1W 5AQ, UK.
584. Department of Pathology, Oslo University Hospital, The Norwegian Radium Hospital, Oslo O310, Norway.
585. Institute of Clinical Medicine and Institute of Oral Biology, University of Oslo, Oslo O310, Norway.
586. Department of Pathology (Research), University College London Cancer Institute, London WC1E 6BT, UK.
587. Research Department of Pathology, University College London Cancer Institute, London WC1E 6BT, UK.
588. East Anglian Medical Genetics Service, Cambridge University Hospitals NHS Foundation Trust, Cambridge CB2 0QQ, UK.
589. Royal National Orthopaedic Hospital - Stanmore, Stanmore, Middlesex HA7 4LP, UK.
590. Division of Orthopaedic Surgery, Oslo University Hospital, Oslo 0379, Norway.
591. Radcliffe Department of Medicine, University of Oxford, Oxford OX3 9DU, UK.
592. University of Pavia, Pavia 27100, Italy.
593. Karolinska Institute, Stockholm SE-171 76, Sweden.
594. University of Oxford, Oxford OX3 9DU, UK.
595. Salford Royal NHS Foundation Trust, Salford M6 8HD, UK.
596. Gloucester Royal Hospital, Gloucester GL1 3NL, UK.
597. Royal Stoke University Hospital, Stoke-on-Trent ST4 6QG, UK.
598. St Thomas's Hospital, London SE1 7EH, UK.
599. Imperial College NHS Trust, Imperial College, London W2 INY, UK.
600. Department of Histopathology, Salford Royal NHS Foundation Trust, Salford M6 8HD, UK.
601. Faculty of Biology, Medicine and Health, University of Manchester, Manchester M13 9PL, UK.
602. Edinburgh Royal Infirmary, Edinburgh EH16 4SA, UK.
603. Barking Havering and Redbridge University Hospitals NHS Trust, Romford RM7 0AG, UK.
604. King's College London and Guy's and St Thomas' NHS Foundation Trust, London SE1 7EH, UK.
605. Cambridge Oesophagogastric Centre, Cambridge University Hospitals NHS Foundation Trust, Cambridge CB2 0QQ, UK.
606. Nottingham University Hospitals NHS Trust, Nottingham NG7 2UH, UK.
607. St Luke's Cancer Centre, Royal Surrey County Hospital NHS Foundation Trust, Guildford GU2 7XX, UK.
608. University of North Carolina at Chapel Hill, Chapel Hill, NC 27599, USA.
609. Norfolk and Norwich University Hospital NHS Trust, Norwich NR4 7UY, UK.
610. University Hospitals Coventry and Warwickshire NHS Trust, Coventry CV2 2DX, UK.
611. University Hospitals Birmingham NHS Foundation Trust, Birmingham B15 2GW, UK.
612. Centre for Cancer Research and Cell Biology, Queen's University, Belfast BT9 7AB, UK.
613. School of Cancer Sciences, Faculty of Medicine, University of Southampton, Southampton SO17 1BJ, UK.
614. Wythenshawe Hospital, Manchester M23 9LT, UK.

615. Barts Cancer Institute, Barts and the London School of Medicine and Dentistry, Queen Mary University of London, London EC1M 6BQ, UK.
616. Royal Marsden NHS Foundation Trust, London and Sutton SW3 6JJ, UK.
617. University Hospital Southampton NHS Foundation Trust, Southampton SO16 6YD, UK.
618. HCA Laboratories, London W1G 8AQ, UK.
619. University of Liverpool, Liverpool L69 3BX, UK.
620. Department of Surgery, Academic Urology Group, University of Cambridge, Cambridge CB2 0QQ, UK.
621. Department of Urology, James Buchanan Brady Urological Institute, Johns Hopkins University School of Medicine, Baltimore, MD 21287, USA.
622. Second Military Medical University, Shanghai 200433, China.
623. Department of Surgery and Cancer, Imperial College, London W2 INY, UK.
624. The Chinese University of Hong Kong, Shatin, NT, Hong Kong, China.
625. Nuffield Department of Surgical Sciences, John Radcliffe Hospital, University of Oxford, Oxford OX3 9DU, UK.
626. Department of Histopathology, Cambridge University Hospitals NHS Foundation Trust, Cambridge CB2 0QQ, UK.
627. Department of Surgery, University of Chicago, Chicago, IL 60637, USA.
628. Laboratory of Pathology, Center for Cancer Research, National Cancer Institute, Bethesda, MD 20892, USA.
629. Canada's Michael Smith Genome Sciences Centre, BC Cancer Agency, Vancouver, BC V5Z 4S6, Canada.
630. Center for Molecular Oncology, Memorial Sloan Kettering Cancer Center, New York, NY 10065, USA.
631. Department of Pathology and Laboratory Medicine, School of Medicine, University of North Carolina at Chapel Hill, Chapel Hill, NC 27599, USA.
632. Department of Population and Quantitative Health Sciences, Case Western Reserve University School of Medicine, Cleveland, OH 44016, USA.
633. Research Health Analytics and Informatics, University Hospitals Cleveland Medical Center, Cleveland, OH 44106, USA.
634. Arnie Charbonneau Cancer Institute, University of Calgary, Calgary, AB T2N 4N2, Canada.
635. Departments of Surgery and Oncology, University of Calgary, Calgary, AB T2N 4N2, Canada.
636. Buck Institute for Research on Aging, Novato, CA 94945, USA.
637. Duke University Medical Center, Durham, NC 27710, USA.
638. USC Norris Comprehensive Cancer Center, University of Southern California, Los Angeles, CA 90033, USA.
639. The Preston Robert Tisch Brain Tumor Center, Duke University Medical Center, Durham, NC 27710, USA.
640. Departments of Dermatology and Pathology, Yale University, New Haven, CT 06510, USA.
641. Fox Chase Cancer Center, Philadelphia, PA 19111, USA.
642. Department of Surgery, Division of Thoracic Surgery, The Johns Hopkins University School of Medicine, Baltimore, MD 21287, USA.
643. University of Michigan Comprehensive Cancer Center, Ann Arbor, MI 48109, USA.
644. University of Alabama at Birmingham, Birmingham, AL 35294, USA.
645. Division of Anatomic Pathology, Mayo Clinic, Rochester, MN 55905, USA.

646. Division of Experimental Pathology, Mayo Clinic, Rochester, MN 55905, USA.
647. International Genomics Consortium, Phoenix, AZ 85004, USA.
648. Departments of Pediatrics and Genetics, University of North Carolina at Chapel Hill, Chapel Hill, NC 27599, USA.
649. Department of Pathology, UPMC Shadyside, Pittsburgh, PA 15232, USA.
650. Center for Cancer Genomics, National Cancer Institute, National Institutes of Health, Bethesda, MD 20892, USA.
651. Department of Neuro-Oncology, Istituto Neurologico Besta, Milano 20133, Italy.
652. The University of Queensland Thoracic Research Centre, The Prince Charles Hospital, Brisbane, QLD 4032, Australia.
653. Department of Neurosurgery, University of Florida, Gainesville, FL 32610, USA.
654. Center for Biomedical Informatics, Harvard Medical School, Boston, MA 02115, USA.
655. Department of Cancer Biology, The University of Texas MD Anderson Cancer Center, Houston, TX 77030, USA.
656. Department of Surgical Oncology, The University of Texas MD Anderson Cancer Center, Houston, TX 77030, USA.
657. Division of Gastroenterology and Hepatology, Mayo Clinic, Rochester, MN 55905, USA.
658. Sylvester Comprehensive Cancer Center, University of Miami, Miami, FL 33136, USA.
659. Department of Internal Medicine, Division of Medical Oncology, Lineberger Comprehensive Cancer Center, University of North Carolina at Chapel Hill, Chapel Hill, NC 27599, USA.
660. University of Tennessee Health Science Center for Cancer Research, Memphis, TN 38163, USA.
661. Centre for Translational and Applied Genomics, British Columbia Cancer Agency, Vancouver, BC V5Z 1L3, Canada.
662. Department of Molecular and Cellular Biology, Baylor College of Medicine, Houston, TX 77030, USA.
663. Department of Pathology and Immunology, Baylor College of Medicine, Houston, TX 77030, USA.
664. Michael E. DeBakey Veterans Affairs Medical Center, Houston, TX 77030, USA.
665. Carolina Center for Genome Sciences, University of North Carolina at Chapel Hill, Chapel Hill, NC 27599, USA.
666. Indivumed GmbH, Hamburg 20251, Germany.
667. Department of Surgery, Division of Hepatobiliary and Pancreatic Surgery, School of Medicine, Keimyung University Dong-san Medical Center, Daegu 41931, South Korea.
668. Women's Cancer Program at the Samuel Oschin Comprehensive Cancer Institute, Cedars-Sinai Medical Center, Los Angeles, CA 90048, USA.
669. Department of Surgery, The George Washington University, School of Medicine and Health Science, Washington, DC 20052, USA.
670. Endocrine Oncology Branch, Center for Cancer Research, National Cancer Institute, National Institutes of Health, Bethesda, MD 20892, USA.
671. National Cancer Center, Gyeonggi 10408, South Korea.
672. ILSbio, LLC Biobank, Chestertown, MD 21620, USA.
673. Canada's Michael Smith Genome Sciences Centre, BC Cancer Agency, Vancouver, BC V5Z 4S6, Canada.

674. Gynecologic Oncology, NYU Laura and Isaac Perlmutter Cancer Center, New York University, New York, NY 10016, USA.
675. Division of Oncology, Stem Cell Biology Section, Washington University School of Medicine, St. Louis, MO 63110, USA.
676. Urologic Oncology Branch, Center for Cancer Research, National Cancer Institute, National Institutes of Health, Bethesda, MD 20892, USA.
677. Institute for Systems Biology, Seattle, WA 98109, USA.
678. Department of Pathology and Laboratory Medicine, Center for Personalized Medicine, Children's Hospital Los Angeles, Los Angeles, CA 90027, USA.
679. Institute for Genomic Medicine, Nationwide Children's Hospital, Columbus, OH 43215, USA.
680. Department of Surgery, Duke University, Durham, NC 27710, USA.
681. Department of Obstetrics, Gynecology and Reproductive Services, University of California San Francisco, San Francisco, CA 94143, USA.
682. Departments of Neurology and Neurosurgery, Henry Ford Hospital, Detroit, MI 48202, USA.
683. Precision Oncology, OHSU Knight Cancer Institute, Oregon Health and Science University, Portland, OR 97239, USA.
684. Department of Pathology, Roswell Park Cancer Institute, Buffalo, NY 14263, USA.
685. Department of Obstetrics and Gynecology, Division of Gynecologic Oncology, Washington University School of Medicine, St. Louis, MO 63110, USA.
686. Penrose St. Francis Health Services, Colorado Springs, CO 80907, USA.
687. Department of Medicine, University of Chicago, Chicago, IL 60637, USA.
688. Department of Neurology, Mayo Clinic, Rochester, MN 55905, USA.
689. Center for Liver Cancer, Research Institute and Hospital, National Cancer Center, Gyeonggi 410-769, South Korea.
690. Department of Genetics, Lineberger Comprehensive Cancer Center, University of North Carolina at Chapel Hill, Chapel Hill, NC 27599, USA.
691. NYU Langone Medical Center, New York, NY 10016, USA.
692. Department of Hematology and Medical Oncology, Cleveland Clinic, Cleveland, OH 44195, USA.
693. Department of Pathology and Laboratory Medicine, University of North Carolina, Chapel Hill, NC 27599, USA.
694. Helen F. Graham Cancer Center at Christiana Care Health Systems, Newark, DE 19713, USA.
695. Cureline, Inc, South San Francisco, CA 94080, USA.
696. Department of Obstetrics and Gynecology, Medical College of Wisconsin, Milwaukee, WI 53226, USA.
697. Hematology and Medical Oncology, Winship Cancer Institute of Emory University, Atlanta, GA 30322, USA.
698. Vanderbilt Ingram Cancer Center, Vanderbilt University, Nashville, TN 37232, USA.
699. Ohio State University College of Medicine and Arthur G. James Comprehensive Cancer Center, Columbus, OH 43210, USA.
700. Research Computing Center, University of North Carolina at Chapel Hill, Chapel Hill, NC 27599, USA.

701. Analytical Biological Services, Inc, Wilmington, DE 19801, USA.
702. Department of Dermatology, University Hospital of Essen, Essen 45122, Germany.
703. University of Pittsburgh, Pittsburgh, PA 15213, USA.
704. Murtha Cancer Center, Walter Reed National Military Medical Center, Bethesda, MD 20889, USA.
705. Brigham and Women's Hospital, Harvard Medical School, Boston, MA 02115, USA.
706. Department of Surgery, Memorial Sloan Kettering Cancer Center, New York, NY 10065, USA.
707. Center for RNA Interference and Noncoding RNA, The University of Texas MD Anderson Cancer Center, Houston, TX 77030, USA.
708. Department of Experimental Therapeutics, The University of Texas MD Anderson Cancer Center, Houston, TX 77030, USA.
709. Department of Gynecologic Oncology and Reproductive Medicine, The University of Texas MD Anderson Cancer Center, Houston, TX 77030, USA.
710. Department of Urology, Mayo Clinic, Rochester, MN 55905, USA.
711. Department of Surgery, Johns Hopkins University School of Medicine, Baltimore, MD 21205, USA.
712. Departments of Neurosurgery and Hematology and Medical Oncology, Winship Cancer Institute and School of Medicine, Emory University, Atlanta, GA 30322, USA.
713. Georgia Regents University Cancer Center, Augusta, GA 30912, USA.
714. Thoracic Oncology Laboratory, Mayo Clinic, Rochester, MN 55905, USA.
715. Institute for Genomic Medicine, Nationwide Children’s Hospital, Columbus, OH 43205, USA.
716. Department of Obstetrics and Gynecology, Division of Gynecologic Oncology, Mayo Clinic, Rochester, MN 55905, USA.
717. International Institute for Molecular Oncology, Poznań 60-203, Poland.
718. Poznan University of Medical Sciences, Poznań 61-701, Poland.
719. Edison Family Center for Genome Sciences and Systems Biology, Washington University, St. Louis, MO 63110, USA.
